# Textural and geochemical constraints on andesitic plug emplacement prior to the 2004–2010 vulcanian explosions at Galeras volcano, Colombia

**DOI:** 10.1007/s00445-018-1260-y

**Published:** 2018-12-07

**Authors:** Amelia A. Bain, Eliza S. Calder, Joaquín A. Cortés, Gloria Patricia Cortés, Susan C. Loughlin

**Affiliations:** 1 Grant Institute, The University of Edinburgh, Edinburgh, UK; 2 Department of Geography, Edge Hill University, Ormskirk, UK; 3 Servicio Geológico Colombiano, Observatorio de Manizales, Manizales, Colombia; 4 The Lyell Centre, British Geological Survey, Edinburgh, UK

**Keywords:** Andesite, Volcanic plug, Vulcanian explosions, Crystallisation, Degassing, Microlite

## Abstract

Hazardous sequences of vulcanian explosions are thought to result from the repeated emplacement and destruction of degassed, highly crystalline magma plugs in the shallow conduit of arc volcanoes. The processes governing the timing and magnitude of these explosions are thought to be related to magma ascent rate and efficiency of degassing and crystallisation. We study a rare suite of time-constrained ballistic bombs from the 2004–2010 period of activity of Galeras volcano to reconstruct magma plug architecture prior to six individual vulcanian explosions. We find that each plug was vertically stratified with respect to crystallinity, vesicularity and melt volatile content, melt composition and viscosity. We interpret this structure as resulting from multiple bubble nucleation events and degassing-driven crystallisation during multi-step ascent of the magma forming the plug, followed by spatially variable crystallisation within the plug under contrasting conditions of effective undercooling created by degassing. We propose that the shallow conduit evolved from more open degassing conditions during 2004–2008 to more closed conditions during 2009–2010. This resulted in explosions becoming smaller and less frequent over time during 2004–2008, then larger and more frequent over time during 2009–2010. This evolution was controlled by changing average ascent rates and is recorded by systematic changes in plagioclase microlite textures. Our results suggest that small volume vulcanian explosions (~ 10^5^ m^3^) should generally be associated with longer repose times (hundreds of days) and produce ballistics characterised by small numbers of large, prismatic plagioclase microlites. Larger volume vulcanian explosions (~ 10^6^ m^3^) should be associated with shorter repose times (tens of days) and produce ballistics characterised by high numbers of small, more tabular plagioclase microlites.

**Electronic supplementary material:**

The online version of this article (10.1007/s00445-018-1260-y) contains supplementary material, which is available to authorized users.

## Introduction

Dense, degassed and highly crystalline plugs are often emplaced in the shallow conduit of arc volcanoes when intermediate magmas ascend at low rates, fostering extensive degassing and crystallisation (Stix et al. 1997b; Sparks 1997; Voight et al. 1999; Hammer et al. 1999; Wright et al. 2007; Clarke et al. 2007). The efficiency of magma outgassing during further crystallisation is thought to control the transition from effusive to explosive behaviour through the build-up of pore overpressure (Sparks 1997). In this paradigm, repetitive vulcanian explosions with numerous interspersed gas and ash venting events represent explosive activity that is embedded within the overarching process of extrusion of degassed, highly crystalline intermediate magmas. This process of extrusion often results in the construction of lava domes, hence the intimate association of lava dome construction and vulcanian activity.

However, due to differences in ascent rate, extrusion rate and composition, conduit processes preceding vulcanian explosions may differ at individual volcanoes and over time at a single volcano, producing observed differences in the repose time, the volume ejected during explosions and the nature of erupted products (Stix et al. 1997b; Hammer et al. 1999; Hammer et al. 2000; Bluth and Rose 2004; Preece et al. 2016). Building understanding of the processes that modulate the magnitude and timing of vulcanian explosions is necessary for successful eruption forecasting, emergency management and hazard mitigation during what are often prolonged volcanic crises. Galeras volcano, Colombia, is an andesitic stratovolcano that exemplifies the repeated development and rupture of volcanic plugs and the transition from effusive dome-building behaviour to explosive vulcanian activity with a range of repose times and ejected volumes (Cortés and Raigosa 1997). In this work, we examine a rare suite of time-constrained, texturally diverse ballistic bombs from the 2004–2010 activity to assess the textural and geochemical properties of andesitic plugs emplaced prior to and destroyed by vulcanian explosions and reconstruct the typical plug architecture.

Due to the hazardous nature of vulcanian explosions, often occurring on a background of relative seismic and outgassing quiescence (Stix et al. 1993), many efforts have been made to understand the emplacement and rupture of magma plugs (Sparks 1997; Voight et al. 1998; Voight 1999; Hammer et al. 1999; Hammer et al. 2000; Wright et al. 2007; Clarke et al. 2007; Giachetti et al. 2010; Burgisser et al. 2010; Burgisser et al. 2011). Plug evolution is thought to begin with the development of bubble networks during slow ascent as a result of decompression-induced degassing, followed by permeability development and outgassing (Clarke et al. 2007). Melt degassing raises the equilibrium liquidus temperature of anhydrous phases, notably that of plagioclase feldspar, and produces effective undercooling in the melt defined as the difference between the melt temperature and the liquidus temperature (Kirkpatrick 1981). This effective undercooling drives the crystallisation of microlites, concentrating volatiles in the residual melt and driving further degassing and crystallisation (Clarke 2013). Variations in the degree of effective undercooling control the resulting microlite textures (Hammer and Rutherford 2002) and these textures may be used to estimate magma ascent rates (Brugger and Hammer 2010b).

The formation of fractures in zones of high strain rate at conduit walls (Tuffen et al. 2003; Gonnermann and Manga 2003), the formation of tuffisite veins during local overpressure events (Kendrick et al. 2016) and the presence of permeable conduit walls (Eichelberger 1995) are also thought to be important contributors to the efficiency of magma outgassing. Under conditions of efficient outgassing and decreasing pore pressures, permeable bubble networks may viscously collapse, resulting in the formation of a dense, degassed, crystal-rich and vertically heterogeneous plug (Stix et al. 1997b; Sparks 1997; Hammer et al. 2000; Wright et al. 2007; Giachetti et al. 2010). Crucially, the porosity, permeability, crystallinity and strength of the magma and its response to decompression (fragmentation threshold and speed of the fragmentation front) are thought to be inter-related and depend on the ascent, degassing and strain history (Heap et al. 2014; Spieler et al. 2004). Accordingly, the processes and timescales involved in the development and destruction of outgassing pathways (bubble networks, fractures and tuffisite veins) have been the subject of much recent research (e.g. Kendrick et al. 2013; Lavallée et al. 2013; Vasseur et al. 2013; Ashwell et al. 2015; Heap et al. 2015; Farquharson et al. 2016; Kushnir et al. 2016a; Kendrick et al. 2016; Kennedy et al. 2016; Kushnir et al. 2016b; Wadsworth et al. 2016; Lamur et al. 2017; Gonnermann et al. 2017).

Detailed geochemical and micro-textural studies of erupted products have significantly enhanced our understanding of the conduit processes occurring prior to vulcanian eruptions of different intensities (Hammer et al. 1999; Hammer et al. 2000; Cashman and McConnell 2005; Clarke et al. 2007; Wright et al. 2007; Giachetti et al. 2010; Burgisser et al. 2010; Burgisser et al. 2011; Wright et al. 2012; Preece et al. 2013; Cassidy et al. 2015; Preece et al. 2016). In this work, we report glass volatile contents, glass and feldspar microlite compositions, and feldspar microlite textural characteristics that shed light on evolving conduit conditions and associated explosions during the 2004–2010 period of activity at Galeras volcano.

## 2004–2010 activity at Galeras volcano

**Fig. 1:**
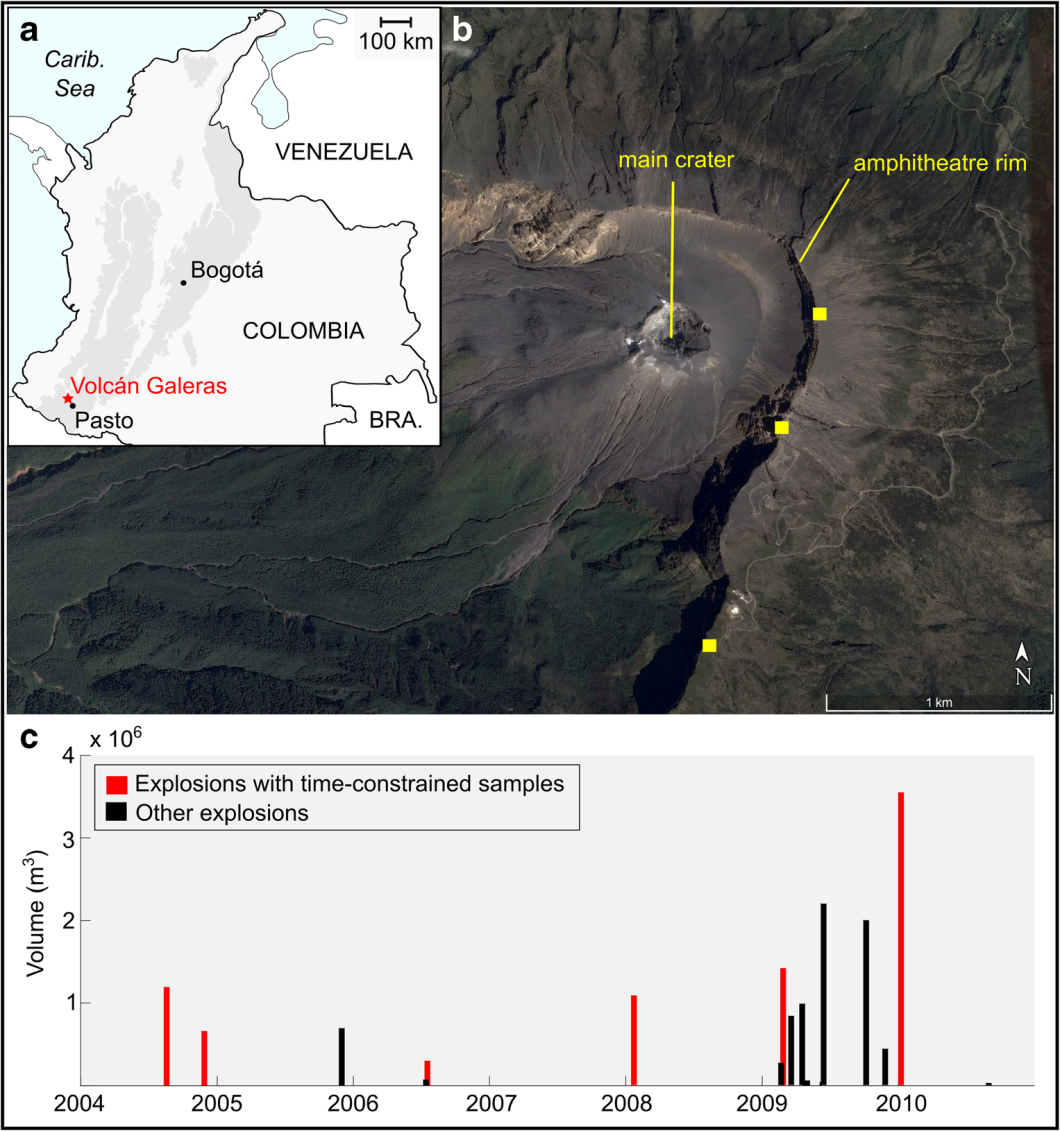
**a** Location of Galeras volcano in Colombia; cordilleras shown in dark grey. **b** Morphology of the Galeras crater area; image from Google Earth (Image © 2017 DigitalGlobe, Image © 2017 CNES/Airbus, Image Landsat/Copernicus). Yellow squares show locations of sample collection. **c** Timing and volume of 2004–2010 explosions. Red bars show explosions for which time-constrained samples were collected

Galeras volcano is a stratovolcano recording 1 Ma of activity, located 8 km northwest of the city of Pasto, Nariño, Colombia (Fig. 1a, b). The most recent phase of activity began 4500 years ago and built the Galeras cone within a horseshoe-shaped scar formed by older caldera-forming and sector collapse events (Calvache et al. 1997). Galeras has been active throughout the twentieth century, with activity characterised by repetitive explosions and dome building (Medina et al. 2017). Monitoring by the Colombian Geological Survey (Servicio Geológico Colombiano, SGC) began in 1988 and Galeras was the focus of intense interdisciplinary study following its designation as a Decade volcano during the United Nations international decade for natural disaster reduction. The 1988–1995 period of activity was well studied and the resultant work features in a special issue of the *Journal of Volcanology and Geothermal Research* (Stix et al. 1997a).

The 2004–2010 period of activity comprised 16 vulcanian explosions and two periods of dome-building activity, interspersed with numerous ash and gas venting events (Medina et al. 2017; Vargas and Torres 2015) (Fig. 1c). Plume heights reached up to 12 km, the volume of material ejected during each vulcanian explosion ranged between 3.5 × 10^4^–3.56 × 10^6^ m^3^ and the repose time between explosions ranged between 1 and 554 days (Vargas and Torres 2015). The total volume of material produced during this period was 21 × 10^6^ m^3^, with 16 × 10^6^ m^3^ ejected during vulcanian explosions and 5 × 10^6^ m^3^ erupted in the form of two domes (personal communication, D. Gómez, SGC, 27/07/2017). Dome growth was first observed on 13 January 2006. Subsequent explosions partially destroyed this dome and renewed dome growth was noted on 18 September 2008. No pyroclastic flows or associated deposits were observed during this period. Eruptive products were limited to fallout deposits and ballistics.

## Methods

### Samples

We examined 38 andesitic ballistic bombs or bomb fragments erupted from the central crater of Galeras volcano during 2004–2010 (Table 1). Twenty-two of these samples have known explosion dates and represent time-constrained samples from the SGC collection (Fig. 1c). The additional samples were collected from the crater rim (Fig. 1b) and correspond more generally to the 2004–2010 period of activity. Selected samples were fresh in appearance, are representative of the textural diversity among ejected ballistics as observed in the SGC collection and the field (from bread-crusted to dense blocks) and ranged in size 6–17 cm. No pumice clasts were produced by these explosions.

**Table 1:** List of samples, eruption dates (where known), bulk vesicularity and textural type

Sample	Eruption date	Bulk vesicularity (%)^a^	Textural type	Sample	Eruption date	Bulk vesicularity (%)^a^	Textural type
*Time-constrained samples*				AB20	2 Jan. 2010	0	Inflated
AB2^b^	11–12 Aug. 2004^c^	0	Dense	AB21^b^	2 Jan. 2010	0.5	Dense
AB3a	11–12 Aug. 2004	0.5	Dense	AB22^b^	2 Jan. 2010	0.5	Inflated
AB3b	11–12 Aug. 2004	0	Dense	*Other samples*			
AB4^b^	11–12 Aug. 2004	0	Inflated	AB23–3	2004–2010	12	Scoriaceous
AB5a	21 Nov. 2004	0.5	Inflated	AB24	2004–2010	3	Inflated
AB5b^b^	21 Nov. 2004	3	Scoriaceous	AB25	2004–2010	7	Scoriaceous
AB6^b^	21 Nov. 2004	0.5	Inflated	AB26	2004–2010	20	Scoriaceous
AB7	21 Nov. 2004	0.5	Inflated	AB27	2004–2010	0.5	Inflated
AB8^b^	12 Jul. 2006	20	Scoriaceous	AB28	2004–2010	0.5	Inflated
AB9^b^	12 Jul. 2006	15	Scoriaceous	AB29	2004–2010	0.5	Inflated
AB10^b^	17 Jan. 2008	0	Inflated	AB30	2004–2010	0	Inflated
AB11	17 Jan. 2008	7	Scoriaceous	AB31	2004–2010	0	Inflated
AB12	17 Jan. 2008	10	Scoriaceous	AB32	2004–2010	25	Scoriaceous
AB13	17 Jan. 2008	0.5	Inflated	AB33	2004–2010	1	Inflated
AB14^b^	17 Jan. 2008	10	Scoriaceous	AB34	2004–2010	0.5	Dense
AB15^b^	17 Jan. 2008	5	Scoriaceous	AB35	2004–2010	0.5	Inflated
AB16^b^	20 Feb. 2009	10	Inflated	AB37	2004–2010	0	Dense
AB17	20 Feb. 2009	0.5	Inflated	AB38^d^	2004–2010	0	Dense
AB18^b^	20 Feb. 2009	5	Dense	AB39	2004–2010	15	Scoriaceous

^a^Bulk vesicularity was estimated from thin sections and is indicated for the rind of inflated bombs only

^b^Time-constrained samples selected for textural study

^c^The 11 August 2004 eruption occurred shortly before midnight and lasted shortly into 12 August 2004

^d^Dense bomb hosting a cataclasite band (see text)

### Ion microprobe analyses

Groundmass glass volatiles were measured by secondary ion mass spectrometry (SIMS) at the Edinburgh Ion Microprobe Facility using a Cameca 1270 instrument, with a focussed beam of O^2−^ ions at 12 keV. Standards included a range of rhyolitic glasses. Species analysed included H, OH, O, ^14^C, S, Cl and F. Also included were ^30^Si, Mg^2+^ and Ca^2+^ to monitor whether microlites were intersected and diluted the volatiles signal due to a partial volume effect. This approach was taken in order to counteract the known difficulty associated with analysing small areas of glass in highly crystalline samples. Total count time was 65 s per cycle (5 s for H, S, Cl and F; 4 s for OH; 6 s for ^14^C; 2 s for Ca^2+^, Mg^2+^, ^30^Si and O) and 15 analytical cycles were collected. The first seven cycles were always discarded to prevent surface contamination. The full analytical methodology is provided in Online Resource 1.

### Electron microprobe analyses

Groundmass glass major element compositions and feldspar microlite major element compositions were analysed using a Cameca SX-100 electron microprobe (EPMA) at the University of Edinburgh with an accelerating voltage of 15 kV. A beam current of 1 nA and beam diameter of 5 μm were used for glass analyses and 2–4 nA and 1 μm diameter for feldspar analyses. Alkalis were always measured first to avoid loss by migration. A natural rhyolitic glass from Lipari and a natural Labradorite standard were used for initial calibration and were analysed on a daily basis to monitor precision and accuracy during the analyses. Analyses with totals outside the range 98–101 wt% oxide were excluded from the results. The stoichiometry of feldspars was checked and cation totals outside 4.8–5.1 were also excluded from the results. Detection limits and measurement standard deviations are reported in Online Resource 2.

Analyses of glass compositions were carried out for most time-constrained bombs in the sample suite (Table 1), with between three and five analyses per sample. Analyses of feldspar microlite compositions were also made for those samples, except in the case of two bombs (AB20 and AB22) where the small size of microlites precluded analyses. In general, analysed microlites measured less than 30 μm in length. Each analysis was performed on a different feldspar microlite, with an average of seven analyses per bomb sample.

### Feldspar microlite textures

Thirteen time-constrained samples were selected for textural study (Table 1), following the methods of Preece et al. (2013). High magnification (× 1500) back-scattered electron (BSE) images of a representative area of groundmass of each sample were collected using a Carl Zeiss SIGMA HD VP Field Emission SEM at the University of Edinburgh. Samples appeared uniform in their groundmass properties under examination with the SEM therefore the selection of the area of groundmass to be imaged was driven by the availability of a large enough area to provide sufficient microlites for statistical significance. Images were stitched using the Zeiss SmartTiffV3 software to produce one continuous area of groundmass per sample (Online Resource 3). Feldspar microlites were identified in the images and outlined manually using Adobe Illustrator (Online Resource 3). Polygons were drawn on traced images using ImageJ (Schneider et al. 2012) to select the groundmass area of interest. Whole microlites not touching the polygon sides were then counted and measured using the best-fitting ellipse tool in ImageJ. The smallest analysed crystals were 2–4 pixels in width, corresponding to sub-micron-sized microlites.

The reference area *A*_R_ for each sample is defined as the phenocryst-free and vesicle-free area available for late-stage crystallisation of groundmass crystals (Hammer et al. 2000). The feldspar microlite areal number density, *N*_A_, was then calculated by dividing the number of whole crystals by the reference area. The feldspar microlite crystallinity, *ɸ*, of the groundmass of each sample was calculated as the fraction of the selected groundmass area that is made up of feldspar, on a vesicle-free basis. CSDSlice (Morgan and Jerram 2006) was used to objectively assess crystal shape parameters in 3D and obtain short (S), intermediate (I) and long (L) axes.

CSDCorrections 1.50 (Higgins 2000) was used to perform stereological calculations on the set of 2D crystal intersections from each image and calculate corresponding 3D crystal size distributions (CSDs). The ImageJ ellipse minor axis measurements were used for the calculations, as these represent the most likely intersections for crystals with prismatic morphologies (Higgins 2000) as obtained from CSDSlice (Table 2). The area percentage of voids and micro-cracks were estimated with ImageJ and used as a correction for the area analysed in CSDCorrections. The resulting three-dimensional data were binned into five size bins per decade and bins containing less than five crystals were removed (Higgins 2000).

**Table 2:** Bomb type, eruption date, volume ejected, repose time prior to the explosion, textural results and calculated growth and nucleation rates

Sample	Type	Eruption date	Volume (× 10^6^ m^3^)	Repose time (days)	*N* ^a^	Area analysed (mm^2^)	*N*_A_ (mm^−2^)^b^	*ϕ* ^b^	Mean crystal area (μm^2^)^b^	S:I:L (*R*^2^)^c^	S/L	
AB2	Dense	11/12 Aug. 2004	1.20	46	1217	0.348	3670 (75)	0.552 (0.024)	115 (12)	1:1.25:5.5 (0.82)	0.18	
AB4	Inflated	11/12 Aug. 2004	1.20	46	1391	0.338	4364 (524)	0.324 (0.057)	56 (25)	1:1.25:7 (0.8)	0.14	
AB5b	Scoriaceous	21 Nov. 2004	0.665	101	1001	0.228	4887 (882)	0.403 (0.073)	54 (4)	1:1.25:7 (0.84)	0.14	
AB6	Inflated	21 Nov. 2004	0.665	101	1818	0.464	3981 (412)	0.342 (0.049)	69 (5)	1:1.25:5.5 (0.81)	0.18	
AB8	Scoriaceous	12 Jul. 2006	0.31	230	1080	0.649	1843 (148)	0.511 (0.049)	153 (36)	1:1.4:4.5 (0.79)	0.22	
AB9	Scoriaceous	12 Jul. 2006	0.31	230	1100	0.577	2187 (220)	0.375 (0.040)	142 (17)	1:1.15:8 (0.79)	0.13	
AB10	Inflated	17 Jan. 2008	1.10	554	3661	0.494	8439 (602)	0.334 (0.051)	31 (4)	1:1.25:2.1 (0.88)	0.48	
AB14	Scoriaceous	17 Jan. 2008	1.10	554	1443	0.505	3441 (359)	0.386 (0.050)	92 (7)	1:1.4:2.8 (0.86)	0.36	
AB15	Scoriaceous	17 Jan. 2008	1.10	554	828	0.178	4796 (328)	0.377 (0.029)	67 (9)	1:1.4:2.8 (0.83)	0.36	
AB16	Inflated	20 Feb. 2009	1.43	400	1433	0.29	5755 (395)	0.403 (0.020)	71(4)	1:1.3:2.1 (0.86)	0.48	
AB18	Dense	20 Feb. 2009	1.43	400	1778	0.379	5098 (355)	0.316 (0.056)	43 (9)	1:1.4:2.2 (0.87)	0.45	
AB21	Dense	2 Jan. 2009	3.56	43	2957	0.477	6437 (308)	0.451 (0.027)	61 (6)	1:1.3:2.1 (0.86)	0.48	
AB22	Inflated	2 Jan. 2009	3.56	43	3737	0.294	12,761 (551)	0.164 (0.038)	11 (1)	1:3:10 (0.68)	0.10	
Sample	ln (*n*_0_ (mm^−4^))	CSD slope α (mm^−1^)	*τ*_min_ (days)	*τ*_max_ (days)	*G*_min_ (mm s^−1^)	*G*_ave_ (mm s^−1^)	*G*_max_ (mm s^−1^)	*J*_min_ (mm^−3^ s^−1^)	*J*_ave_ (mm^−3^ s^−1^)	*J*_max_ (mm^−3^ s^−1^)	*N*_V_ (mm^3^)	*L*_c_ (μm)
AB2	18.17	− 111	46	46	2.27 × 10^−9^	2.27 × 10^−9^	2.27 × 10^−9^	0.177	0.177	0.177	703,202	9
AB4	18.49	− 122	46	46	2.06 × 10^−9^	2.06 × 10^−9^	2.06 × 10^−9^	0.221	0.221	0.221	877,900	8
AB5b	18.33	− 100	101	147	7.87 × 10^−10^	9.67 × 10^−10^	1.15 × 10^−9^	0.072	0.089	0.105	916,377	10
AB6	18.90	− 157	101	147	5.01 × 10^−10^	6.15 × 10^−10^	7.29 × 10^−10^	0.081	0.100	0.118	1,029,513	6
AB8	18.17	− 139	230	745	1.11 × 10^−10^	2.36 × 10^−10^	3.61 × 10^−10^	0.009	0.018	0.028	555,137	7
AB9	17.39	− 97	230	745	1.60 × 10^−10^	3.39 × 10^−10^	5.18 × 10^−10^	0.006	0.012	0.018	365,521	10
AB10	21.60	− 467	554	1299	1.91 × 10^−11^	3.19 × 10^−11^	4.47 × 10^−11^	0.046	0.076	0.107	5,117,795	2
AB14	18.95	− 178	554	1299	5.00 × 10^−11^	8.36 × 10^−11^	1.17 × 10^−10^	0.009	0.014	0.020	957,338	6
AB15	19.78	− 216	554	1299	4.13 × 10^−11^	6.90 × 10^−11^	9.68 × 10^−11^	0.016	0.027	0.038	1,812,551	5
AB16	20.61	− 345	400	1699	1.97 × 10^−11^	5.18 × 10^−11^	8.38 × 10^−11^	0.018	0.046	0.075	2,588,671	3
AB18	20.85	− 368	400	1699	1.85 × 10^−11^	4.86 × 10^−11^	7.86 × 10^−11^	0.021	0.055	0.089	3,069,947	3
AB21	20.95	− 377	43	2015	1.52 × 10^−11^	3.65 × 10^−10^	7.14 × 10^−10^	0.019	0.458	0.898	3,335,188	3
AB22	20.79	− 131	43	2015	4.39 × 10^−11^	1.05 × 10^−9^	2.06 × 10^−9^	0.047	1.126	2.204	8,188,227	8

^a^*N* is the number of whole crystals analysed for each sample (crystals touching edges were excluded)

^b^Error is given as the standard deviation of values for image analysis on the top, bottom, left and right halves of each image

^c^*R*^2^ value from CSDslice (Morgan and Jerram 2006)

## Results

### Petrology

The petrology of the sample suite is uniform overall. All samples consist of porphyritic andesite (SiO_2_ 58.13–59.21 wt%, K_2_O + Na_2_O 5.4–5.47 wt%; personal communication, G.P. Cortés, SGC, 21/10/2015), with phenocrysts of plagioclase (20–25%), clinopyroxene and orthopyroxene (7–10%) and Fe–Ti oxides (3–5%). Plagioclase phenocrysts frequently display sieve-textured zones. Glomerocrysts of the main phenocryst phases from several millimetres up to several centimetres are frequently present. Rare micro-phenocrysts of olivine and amphibole may be found, with amphibole consistently bearing an opaque reaction rim when present. Apatite occurs as an accessory phase. The dominant groundmass phase is plagioclase, although microlites of pyroxene and Fe–Ti oxides also occur. The proportion of groundmass glass and groundmass crystallinity vary widely. All glasses are rhyolitic in composition (SiO_2_ 74.5–77.7 wt%, K_2_O + Na_2_O 8.12–8.75 wt%).

### Morphological and textural description of bomb samples

**Fig. 2:**
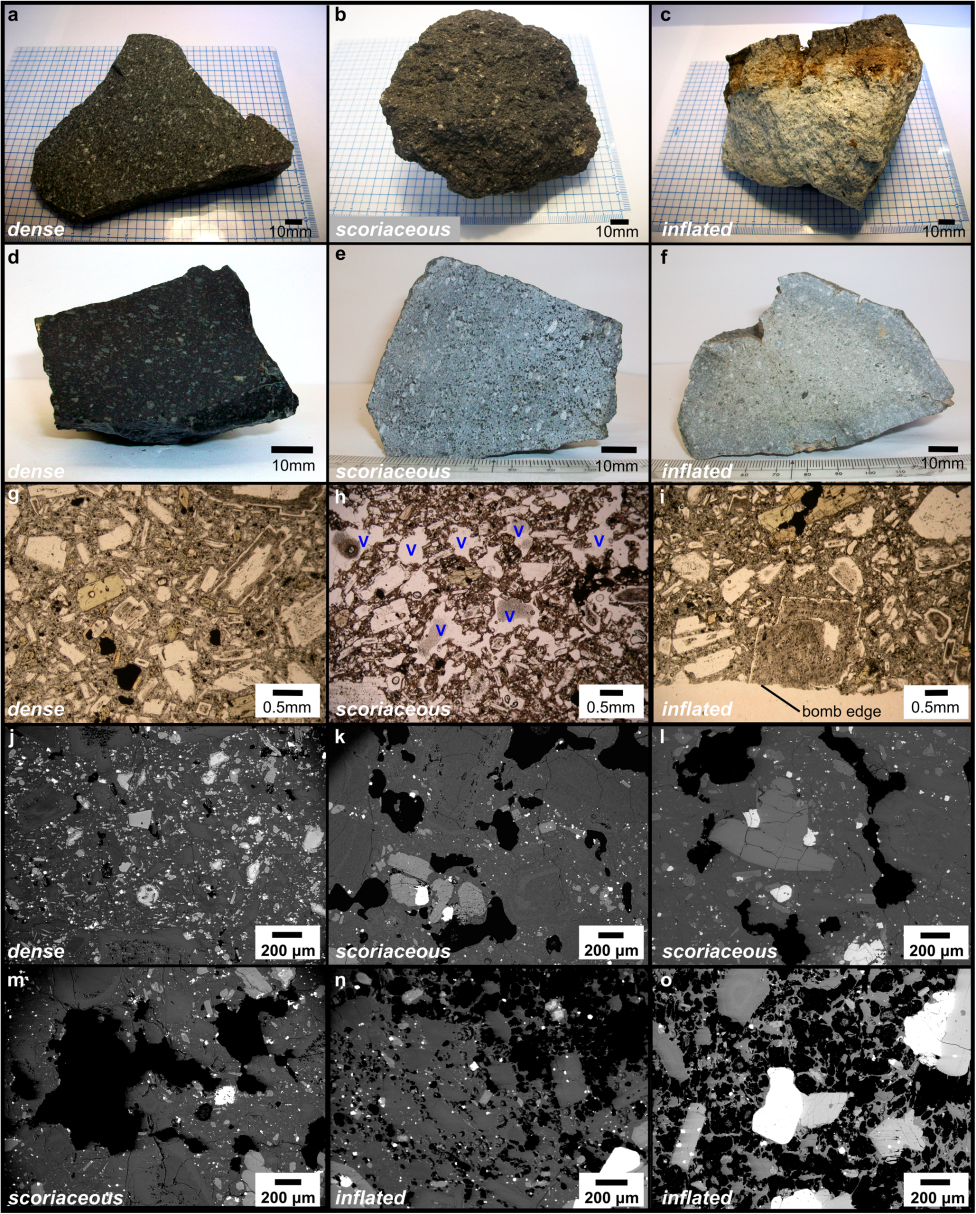
Representative hand samples and micro-textures of dense, scoriaceous and inflated bomb types. **a** Intact dense bomb. **b** Intact scoriaceous bomb. **c** Inflated bomb fragment. **d** Cross-sectional cut through a dense bomb. **e** Cross-sectional cut through a scoriaceous bomb. **f** Cross-sectional cut through an inflated bomb. **g** Plane polarised light thin section image of a dense bomb. **h** Plane polarised light image of a scoriaceous bomb (V indicates vesicles). **i** Plane polarised light image of an inflated bomb rind (no interior vesicular material shown). **j** BSE image of a dense bomb. **k**–**m** BSE images of scoriaceous bombs. **n** BSE image of the transition zone between the interior and the rind of an inflated bomb. **o** BSE image of the vesicular interior of an inflated bomb. In all BSE images, void space is black, glass and plagioclase crystals are dark grey, pyroxene crystals are light grey and Fe–Ti oxides are white

Despite the overall petrological homogeneity, bombs were classified into three distinct textural types on the basis of morphology and texture (Fig. 2 and Online Resource 4). These textural types are dense bombs, scoriaceous bombs and inflated bombs.

#### Dense bombs

Dense bombs display faceted morphologies in hand sample (Fig. 2a), with no textural contrast between the interior and exterior (Fig. 2d), except for a wafer-thin outer surface featuring a glassy lustre. The overall vesicularity is very low (0–5%) (Fig. 2g), ranging from a complete lack of vesicles visible in thin section to small numbers of vesicles up to 1 mm across that may be elliptical or polylobate in shape (Fig. 2j). Many samples host tuffisite veins on various scales, from several hundred microns to several millimetres wide. These consist of variably annealed andesite clasts that are separated from the homogeneous host andesite by sharp contacts with broken crystals present on both sides. Linear or irregular shaped voids or linear trains of voids may mark the boundary between clasts in the tuffisite veins and cristobalite is occasionally found infilling these voids (Online Resource 5, S1).

One dense bomb sample (AB38) hosts a cataclasite band 2–11 mm thick with irregular edges. In this zone, phenocrysts are shattered and crystal fragments are sheared (Online Resource 5, S2). Void spaces exist in the shadow of some phenocrysts and cristobalite fills many void spaces throughout this zone of deformation. Cross-cutting tuffisite veins have a markedly higher porosity than the cataclasite, with a distinct lack of sheared phenocrysts and with cristobalite often infilling voids.

#### Scoriaceous bombs

Scoriaceous bombs (Fig. 2b) typically have irregular, non-facetted shapes in hand sample and no textural contrast between the interior and exterior (Fig. 2e). The outer surface typically lacks the glassy lustre of dense bombs and features a higher number of vesicles. Scoriaceous bombs typically have a higher vesicularity than dense bombs (3–25%), but the vesicle shapes and range of vesicularity define a gradual textural transition in vesicularity between these bomb types (Online Resource 4). When viewed in thin section, larger vesicles (> 1 mm) in scoriaceous bombs tend to have polylobate or branching shapes (Fig. 2h, k–m). Smaller vesicles (generally < 1 mm) tend to be more elliptical in shape. Vesicle walls frequently host cristobalite crystals. In several samples, large vesicles are partially or completely in-filled with andesitic clasts, consisting of broken crystal phases and matrix glass. This clastic material also occurs in small fractures between vesicles, forming an irregular network akin to the tuffisite veins in dense bombs (Online Resource 5, S3).

#### Inflated bombs

Inflated bombs typically display dense or very poorly vesicular bread-crusted rinds (typically 0–0.5% but occasionally up to 10%) (Fig. 2c, i). Rinds range from a few millimetres to a few centimetres in thickness. Inflated bomb interiors are typically highly micro-vesicular to pumiceous (Fig. 2c, f and Online Resource 4). When viewed in thin section, vesicles in the rind are elliptical or polylobate where present. Vesicles in the interior are much more numerous, much smaller (on the order 1–10 μm) and spherical to elongated or coalesced (Fig. 2n, o). The transition between the interior and the rind is usually gradational (Fig. 2n). Many samples host tuffisite veins up to 0.5 cm in width that may traverse the bomb interior as well as the rind. The porosity of these veins is typically higher than that of the bomb rind and lower than the bomb interior when examined in BSE images (Online Resource 5, S4).

### Groundmass glass volatiles

**Fig. 3:**
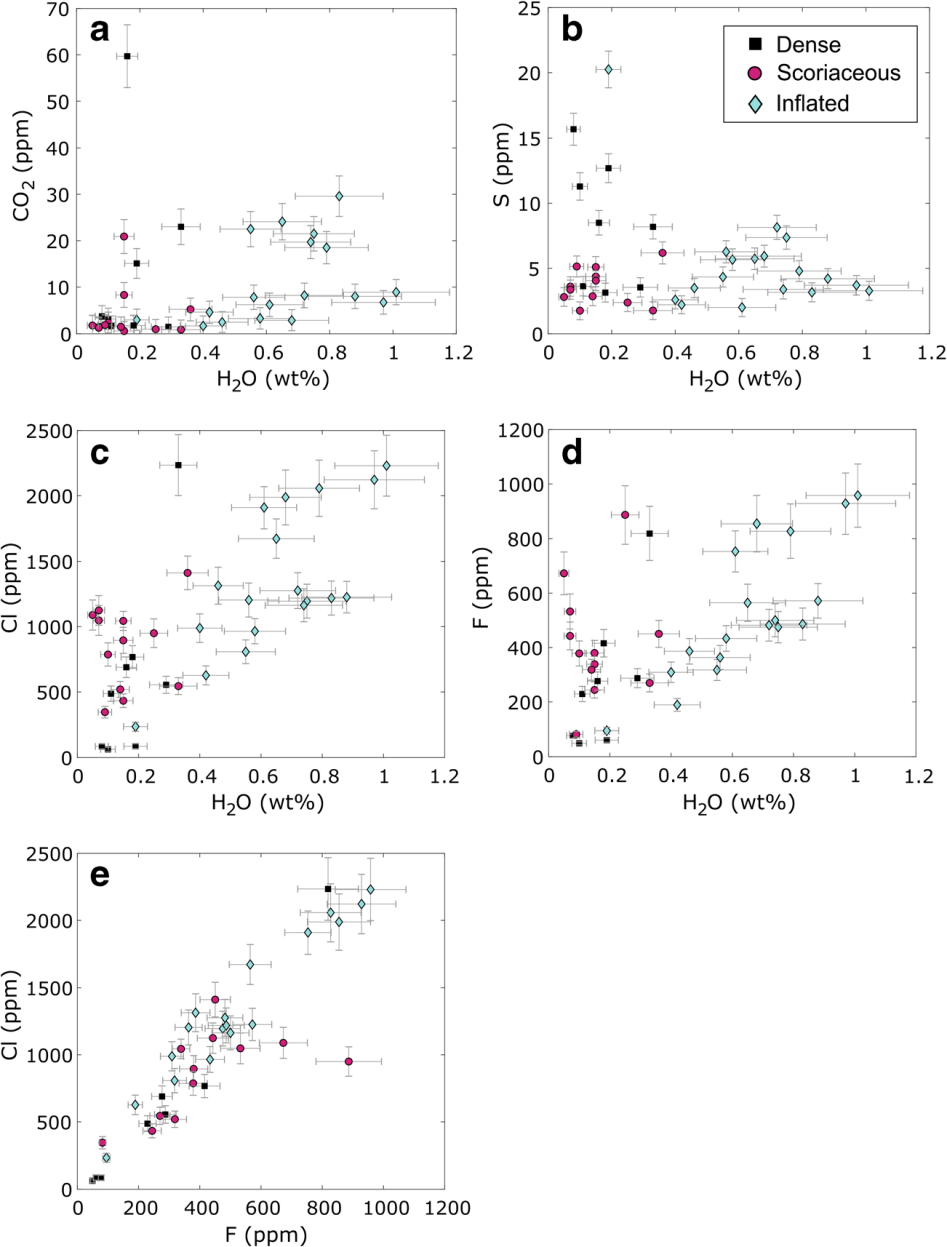
**a**–**f** Volatile content of groundmass glasses measured by SIMS. Each data point represents the median of analyses on one bomb sample. Error bars are given as two sigma calculated from repeatability on standards and a background correction. Different symbols represent different bomb types (see legend)

Median values of all volatile analyses carried out on the groundmass glass of each bomb sample are taken to represent the volatile content for that sample (Fig. 3, Online Resource 6). Only glasses from the rapidly quenched rinds of inflated bombs were analysed in order to avoid areas that experienced syn-eruptive volatile loss (Wright et al. 2007).

Water in groundmass glasses is in the range 0.05–1.01 wt% (Fig. 3a). Inflated bomb rinds have the highest water contents with > 0.4 wt%, except for one outlier with 0.19 wt% that corresponds to a bomb featuring evidence of a centimetre-scale tuffisite vein in the rind (AB28, Online Resource 5, S5). In contrast, the groundmass glasses of dense and scoriaceous bombs have lower H_2_O contents, with 0.08–0.33 wt% H_2_O and 0.05–0.36 wt% respectively.

Carbon dioxide is in the range 1–30 ppm (Fig. 3a), excluding the dense bomb that hosts the cataclasite vein (AB38) with 60 ppm, which constitutes a notable outlier. Inflated bomb rinds show a trend of decreasing CO_2_ content with decreasing H_2_O whereas dense and scoriaceous bombs typically have very low CO_2_ and H_2_O contents. Most inflated bombs have higher CO_2_ content than dense and scoriaceous bombs, except in the case of five dense/scoriaceous bombs that have higher CO_2_ contents than the others (5–23 ppm).

Sulphur is in the range 2–20 ppm (Fig. 3b). Inflated bomb rinds show a scattered trend of decreasing S with decreasing H_2_O. Scoriaceous bombs have a restricted, low range of S (2–6 ppm) whereas dense bombs have a higher range of S (3–16 ppm), with four bombs showing higher values than most others samples. F and Cl are in the range 49–958 ppm and 63–2234 ppm, respectively, and show a strong positive correlation (Fig. 3e). Inflated bomb rinds show a clear trend of decreasing F and Cl with decreasing H_2_O. In contrast, dense and scoriaceous bombs show a wide spread of F content with respect to their small and restricted range of H_2_O and cover broadly the same range of F content as inflated bombs. Dense bombs show overall low Cl whereas scoriaceous bombs have a larger range of Cl, with most scoriaceous bombs having higher Cl than most dense bombs. The inflated bomb rind with the distinctly low H_2_O content (AB28) records a higher S content and much lower F and Cl than other inflated bomb rinds (Fig. 3b–d).

### Groundmass glass compositions

**Fig. 4:**
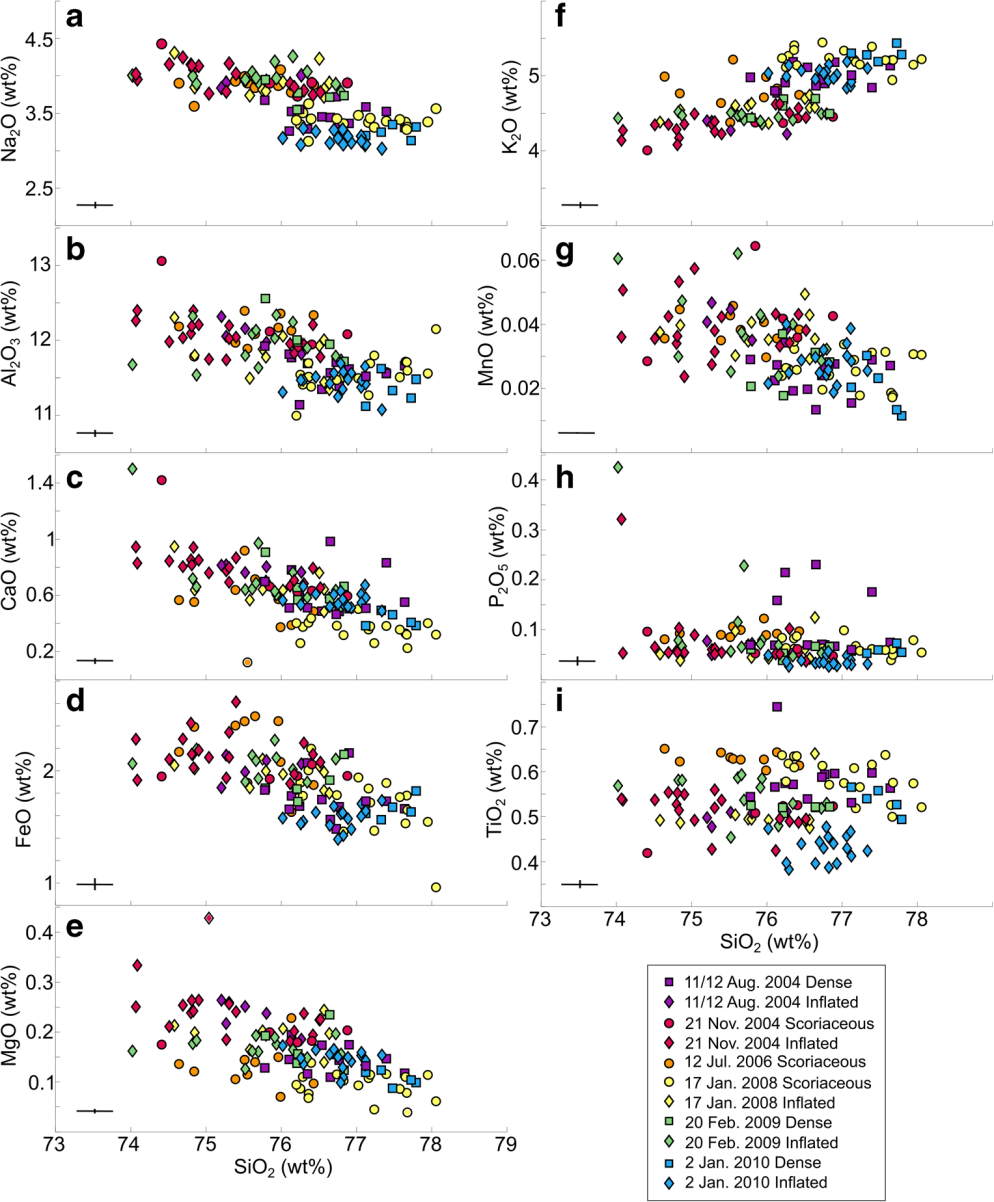
**a**–**i** Composition of groundmass glasses measured by EPMA. Each point represents one analysis. Squares correspond to analyses on dense bombs, circles on scoriaceous bombs and diamonds on inflated bombs. Colours correspond to different explosions. Symbols and colours are used consistently throughout subsequent figures. Error bars in each panel represent the average measurement standard deviation

In the sample suite as a whole, K_2_O wt% increases as SiO_2_ wt% increases in the rhyolitic glasses while Na_2_O, Al_2_O_3_, CaO, MgO, FeO* and MnO wt% decrease (Fig. 4). The oxide P_2_O_5_ remains approximately constant as SiO_2_ increases and there is no consistent relationship between TiO_2_ and SiO_2_ wt%. Among contemporaneous time-constrained samples produced in a single explosion (Table 1), glasses from dense or scoriaceous bombs are typically more SiO_2_ and K_2_O-rich and depleted in Al_2_O_3_, Na_2_O, CaO, MgO, FeO^*^ and MnO wt% compared to the associated inflated bombs. In addition, among samples produced in a single explosion, TiO_2_ wt% may either increase (e.g. 2 January 2010) or decrease (e.g. 20 February 2009) with SiO_2_ wt%. For example, time-constrained samples from the latest explosion (2 January 2010) in this study show increasing Na_2_O, FeO^*^ and TiO_2_ with increasing SiO_2_ wt%, whereas samples from earlier explosions show the inverse relationship.

### Feldspar microlite compositions

**Fig. 5:**
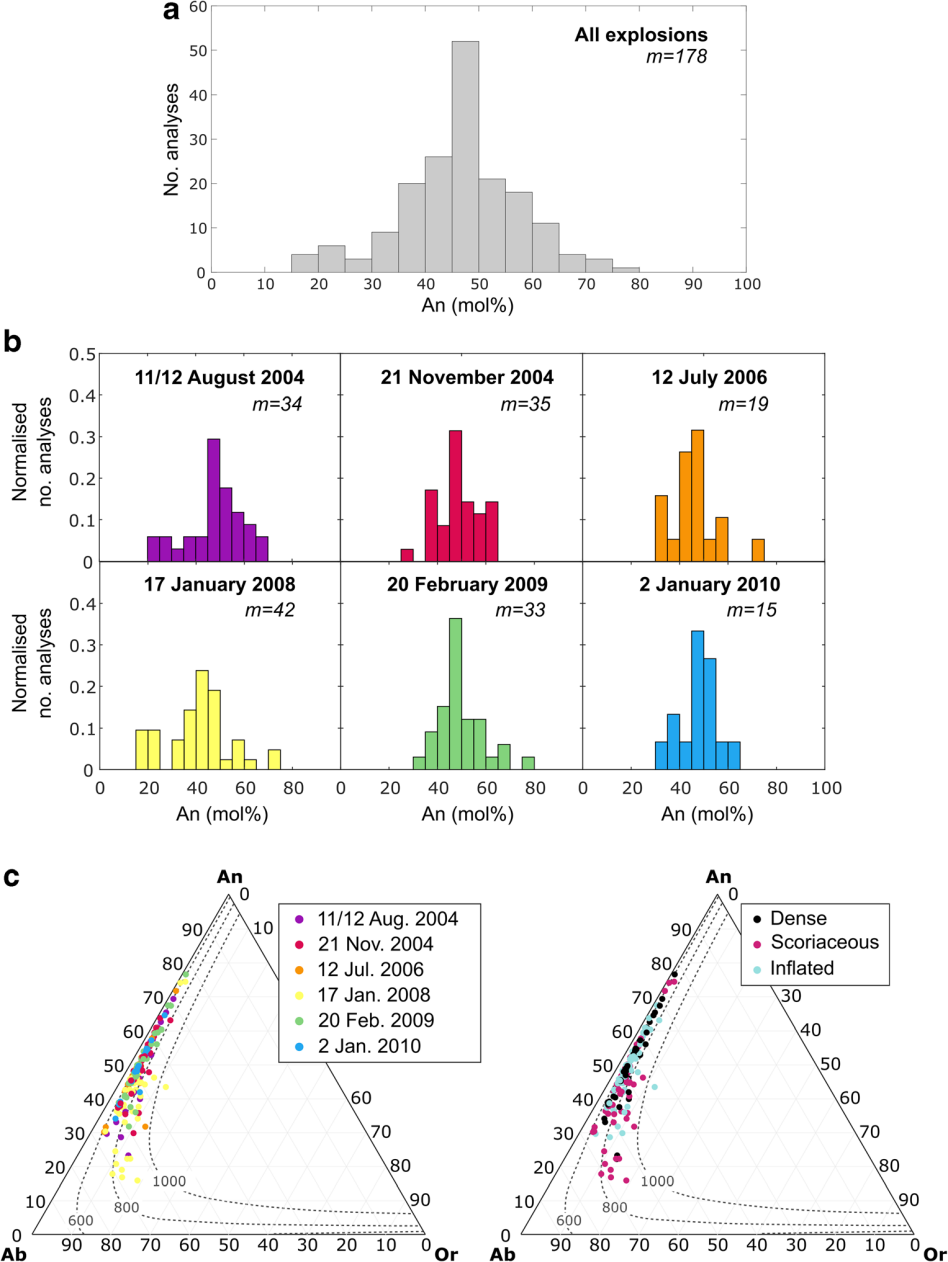
**a** Frequency distributions of feldspar microlite An content from EPMA analyses on samples from all explosions in this study; each analysis was performed on a different microlite. **b** Feldspar microlite An content frequency distributions for samples belonging to individual explosions, normalised by the number of analyses performed for samples from that explosion (*m* indicates the number of analyses). **c** Feldspar microlite ternary diagrams organised by explosion date and by bomb type. Also shown are isothermal sections of the dry ternary solvus over the range 1–10 MPa and at 600 °C, 800 °C and 1000 °C (Wen and Nekvasil 1994)

Feldspar microlite compositions were analysed in order to shed light on the range of crystallisation conditions. Anorthite (An) contents of feldspar microlites cover a large range (An_16_–An_77_), with a bimodal distribution where the dominant peak is in the range An_45_–An_50_ and a smaller mode exists in the range An_20_–An_25_ (Fig. 5a). Histograms of feldspar An content for groups of contemporaneous time-constrained samples from individual explosions (Table 1) also consistently show a dominant mode in the range An_40_–An_50_, typically with a secondary, more variable mode in the range An_15_–An_40_ (Fig. 5b). Although results for the latest explosion in this study (2 January 2010) must be treated with caution due to the impossibility of analysing very small microlites in the inflated bomb samples, this explosion shows the most restricted range of feldspar microlite An content (An_34_–An_64_) and the explosion that occurred on 17 January 2008 shows the largest range in An content (An_16_–An_74_). These explosions correspond to the shortest (43 days) and the longest (554 days) repose times, respectively.

**Fig. 6:**
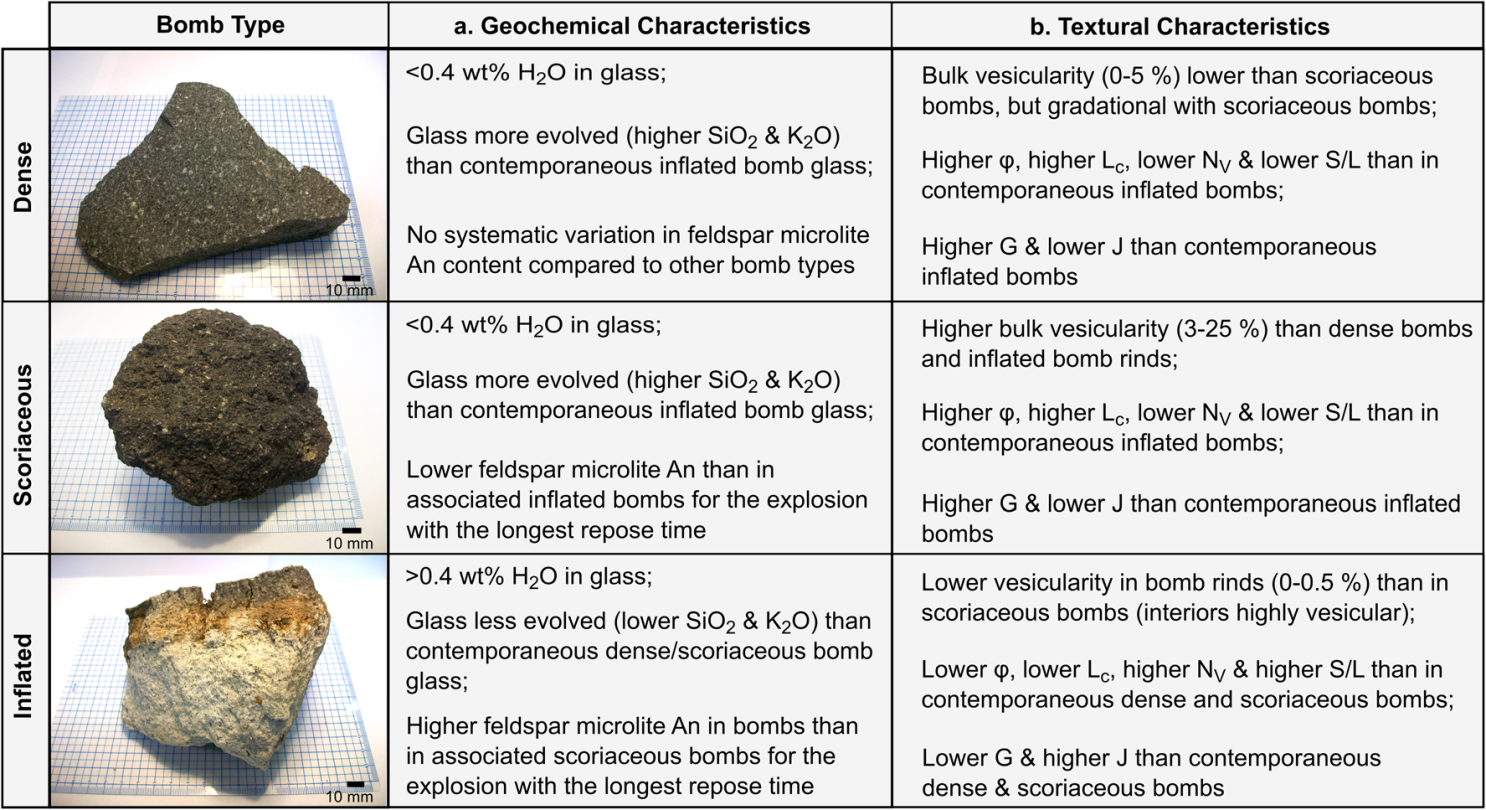
Summary of the typical geochemical and groundmass textural characteristics of each bomb type

Figure 5c shows feldspar microlite compositions in the An-Ab-Or ternary system along with isothermal sections of the dry ternary solvus over the pressure range 1–10 MPa (Wen and Nekvasil 1994). Most analysed microlites are plagioclase (bytownite to oligoclase), with just one microlite falling in the alkali feldspar-anorthoclase field. There is no clear pattern of feldspar composition according to bomb type and the ranges of compositions of feldspars found in different bomb types mostly overlap, except in the case of the explosion that occurred on 17 January 2008 where the range of compositions in the scoriaceous bombs is clearly shifted to lower An% compared to contemporaneous inflated bombs. Results of all EPMA glass and feldspar analyses are provided in Online Resource 2. Figure 6a summarises the key geochemical characteristics of each bomb type.

### Feldspar microlite textures

#### Batch textural parameters

**Fig. 7:**
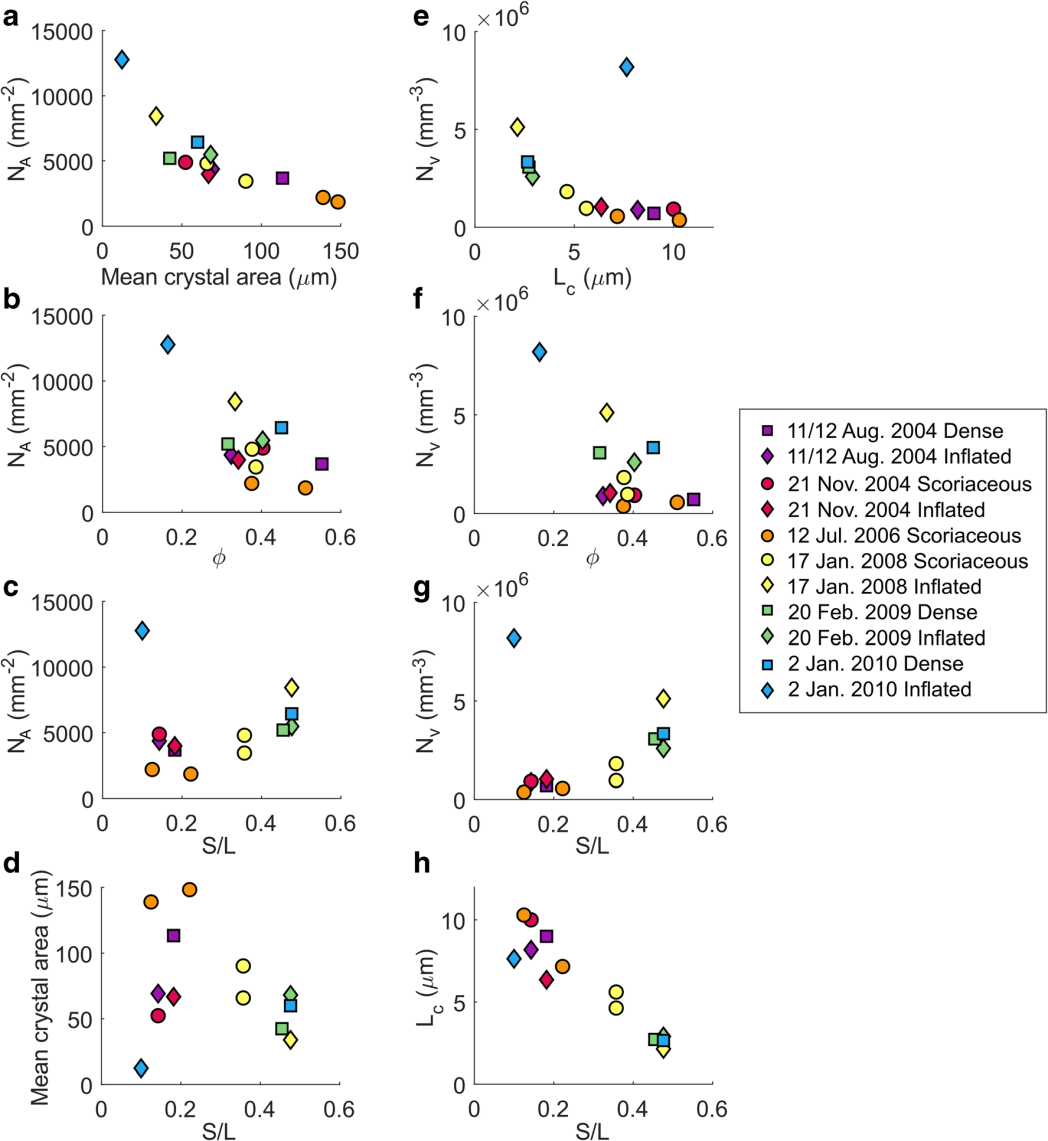
**a**–**d** Batch textural results following Preece et al. (2013, 2016). **e**–**h** Calculated three-dimensional textural results based on regression lines on the steepest portion of the CSD of each sample in the smallest size bins (dashed lines in Fig. 8). Symbols and colours represent bomb types and explosion dates respectively (see legend)

The areal feldspar number density (*N*_A_) in texturally characterised samples ranges from 1843 to 12,761 mm^−2^ and groundmass feldspar crystallinity (*ɸ*) ranges from 0.164 to 0.552. Mean crystal areas range from 11 to 153 μm^2^ and crystal aspect ratios (taken as S/L) obtained from CSDSlice shape parameters are in the range 0.1–0.48 (Table 2). The inflated bomb (AB22) from the latest explosion for which time-constrained samples are available (2 January 2010) constitutes a notable outlier with higher *N*_A_, lower *ɸ* and lower mean crystal area compared to other samples (Fig. 7a, b) and is the only bomb with skeletal microlites (Online Resource 3).

There is a strong inverse relationship between *N*_A_ and mean crystal area, indicating that samples have either a high number of small microlites or a lower number of larger microlites (Fig. 7a). There is also a generally inverse relationship between *N*_A_ and *ɸ* (Fig. 7b), indicating that samples with high crystal number densities are generally less crystalline and vice versa, although the relationship is poorly defined. *N*_A_ and crystal aspect ratio, S/L, are positively correlated (omitting sample AB22), indicating that samples with the lowest *N*_A_ have the most prismatic microlites and samples with the highest *N*_A_ have more tabular microlites (Fig. 7c). The relationship between mean crystal area and S/L is more complex, with a maximum mean crystal area at S/L of around 0.22 (Fig. 7d). Both *N*_A_ and the mean crystal area are two-dimensional results that must be treated with caution as *N*_A_ varies as a function of crystal size due to the intersection probability effect (large crystals are more likely to be intersected by the thin section plane than small crystals, producing a higher *N*_A_; Royet 1991) and mean crystal area depends on the size cut-offs of analysed crystals (Higgins 2006). These will be compared with the three-dimensional results from CSDs in the following section, to assess whether batch textural parameters capture the key textural information in these samples.

Overall, batch textural parameters indicate that groundmass textures range from more glassy with many small, more tabular crystals to more crystalline with fewer, larger and more prismatic crystals. AB22 stands out as having a very high areal number density and acicular microlites compared to other samples, which belong to the dominant group with lower *N*_A_ and larger mean crystal areas (Table 2). Inflated bombs tend to have higher *N*_A_, smaller mean crystal areas and lower crystallinities than contemporaneous scoriaceous or dense bombs. In addition, samples from the three earliest explosions for which time-constrained samples are available (11/12 August 2004, 21 November 2004, 12 July 2006) tend to have overall lower *N*_A_, higher *ɸ*, higher mean crystal areas and lower S/L than samples from the three latest explosions (17 January 2008, 20 February 2009, 2 January 2010).

#### Crystal size distributions

**Fig. 8:**
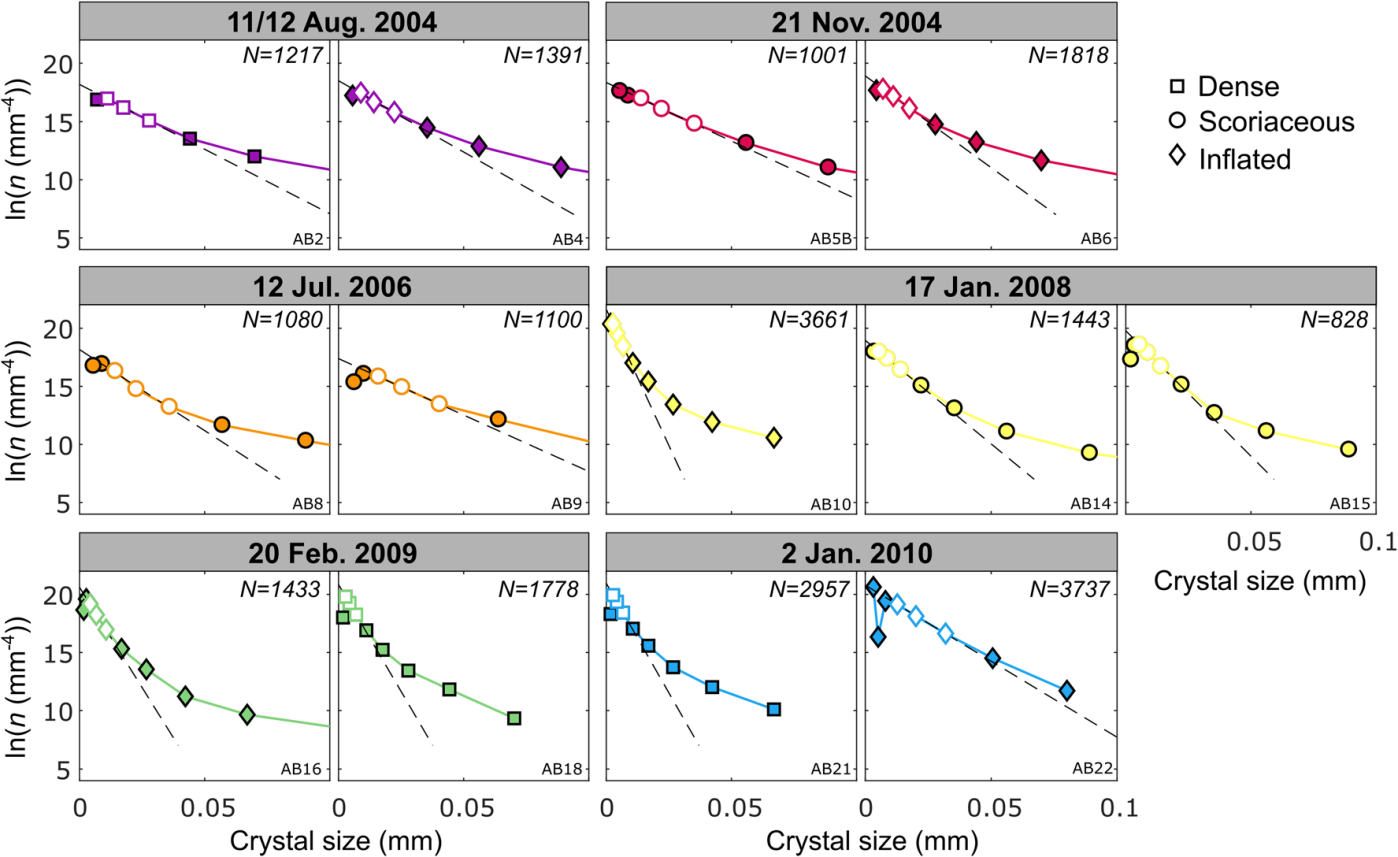
Three-dimensional crystal size distributions for the subset of time-constrained samples selected for textural study. The population density *n* corresponds to the number of crystals per volume per length. Data point symbols correspond to different bomb types (see legend). Dashed lines are regression lines using the three points on the steepest segment of each CSD (white symbols), omitting the downturn at small sizes. Minimum errors from counting statistics given by CSDCorrections (Higgins 2000) are smaller than the symbol size (see Online Resource 7). The number of crystals measured in each sample is indicated by *N*

Crystal size distributions (CSDs) provide stereologically corrected, three-dimensional textural information that does not depend on the size cut-off of the analysed crystals (Higgins 2006). The CSDs calculated for feldspar microlites in the selected samples are concave-up in form (Fig. 8). Sample AB22 is the least concave and closest to a constant-slope (straight) CSD. The downturn observed in the smallest size bins in each CSD is attributed to incomplete compensation in the stereological calculations for the intersection probability effect for the smallest crystals (Brugger and Hammer 2010a). Following Brugger and Hammer (2010a) and Preece et al. (2016), the data in these bins are discounted from further analysis.

The slope of a straight CSD, *α* (in mm^−1^), and the *y* intercept *n*_0_, where *n*_0_ is the final nuclei population density in mm^−4^, are related to the average crystal growth rate *G* (in mm s^−1^), the crystallisation time *τ* (in s) and the average nucleation rate *J* (in mm^−3^ s^−1^) (Cashman 1988; Marsh 1988) as *α* = − 1/*Gτ* and *J* = *n*_0_*G*. In the case of a concave-up CSD, the final growth rate *G*_f_ within a magma parcel stored in the conduit prior to an explosion may be determined from a regression line taken on the steepest portion of the CSD in the smallest size bins, using the three points immediately following the artificial downturn (Brugger and Hammer 2010a; Preece et al. 2016) (Fig. 8). In order for *G*_f_ to be determined, an appropriate crystallisation time *τ* must be selected. As crystallisation times are unknown, two estimates of *τ* were used to bracket growth rates prior to each explosion. A maximum residence time *τ*_max_ for microlites growing as a result of decompression-driven degassing can be obtained by using the first increase in seismicity above background rates detected in monitoring data (personal communication, D. Gómez, SGC, 27/07/2017) as an indication of the onset of magma ascent from a shallow crustal storage region. Conversely, by assuming that the initial ascent was rapid enough that all crystallisation occurred at shallow levels, a minimum crystallisation time *τ*_min_ can be defined as the time between an explosion and the onset of the previous explosion in the sequence. For the explosions that occurred on 12 July 2006 and 20 February 2009, small volume explosions (8 × 10^4^ m^3^ and 3.1 × 10^5^ m^3^ respectively) that occurred shortly before were omitted from the calculation of *τ*_min_ as this would lead to unrealistically short crystallisation times (4 and 6 days respectively). The maximum residence time *τ*_max_ determined in this way ranges from 46 to 2015 days and *τ*_min_ ranges from 43 to 554 days. Minimum crystal growth rates *G*_min_ and maximum growth rates *G*_max_ for the three smallest size bins in each CSD are in the range 10^−11^–10^−9^ mm s^−1^ (Table 2).

**Fig. 9:**
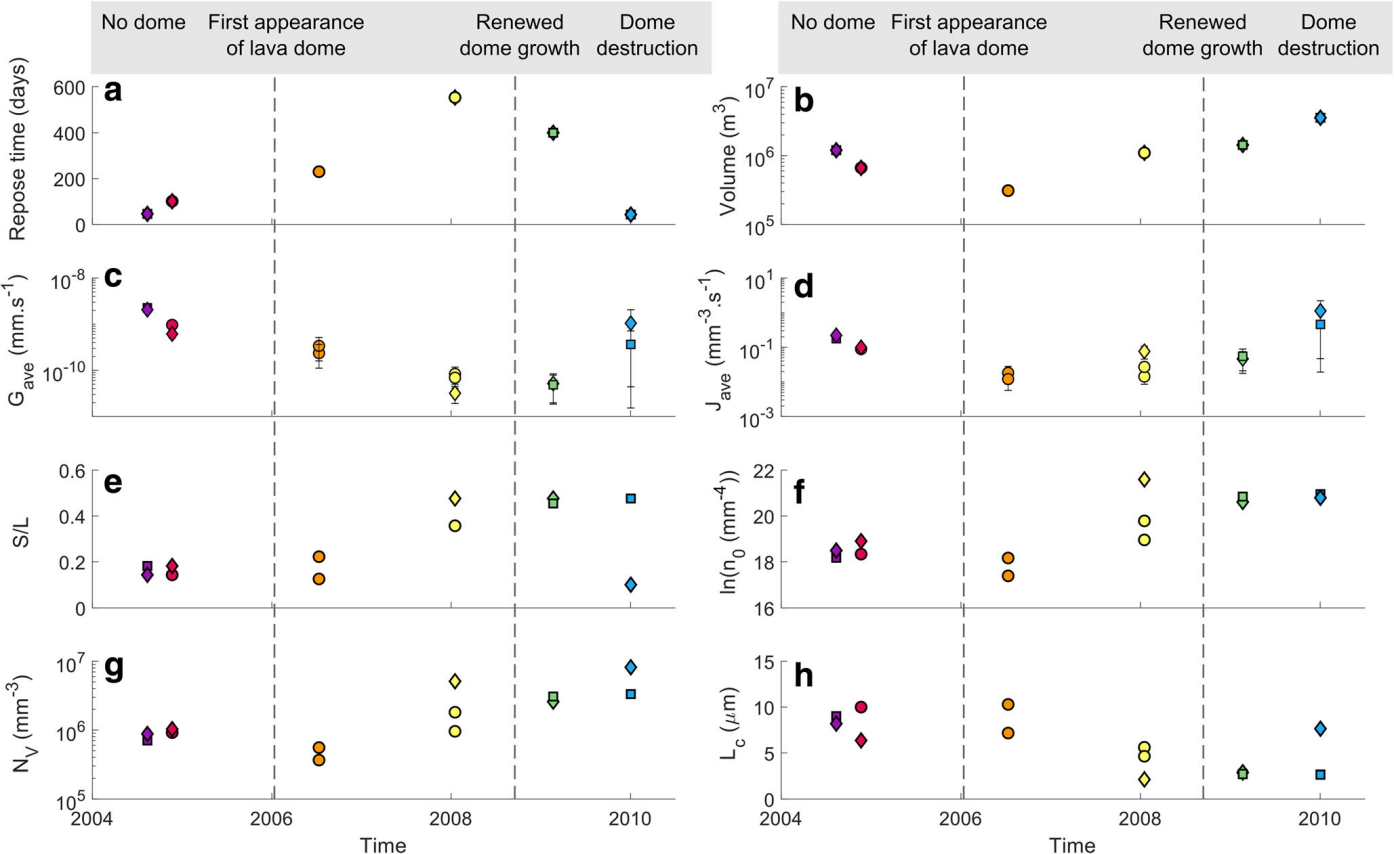
Evolution of repose time prior to explosions, erupted volume, growth and nucleation rate, and textural characteristics over the course of the eruption sequence for the six explosions in this study. Also indicated are the dates when dome growth was observed. Bars in **c** and **d** represent minimum and maximum rates (*G*_min_, *G*_max_ and *J*_min_, *J*_max_)

We use G_ave_, calculated as the average of *G*_min_ and *G*_max_, as an estimate for the final feldspar microlite growth rate in each sample immediately prior to its eruption. We also estimate average nucleation rates, *J*_ave_, in each sample immediately prior to eruption from *G*_min_, *G*_max_ and *n*_0_, and we use *G*_ave_ and *J*_ave_ to track changing crystallisation conditions in individual batches of magma arriving in the shallow conduit, forming plugs and feeding explosions between 2004 and 2010. These results show that the final crystal growth rate recorded in each magma plug prior to the vulcanian explosions decreased over time in the period during which the first four explosions of the studied sequence occurred (August 2004–January 2008) (Fig. 9c). After this period, crystal growth rates decreased slightly. The uncertainty for the latest explosion for which time-constrained samples are available (2 January 2010) is large as *τ*_max_ is large, but the small mean crystal area and high S/L in the dense bomb from this explosion (Fig. 7d) suggest that growth rates were low. Growth rates were typically higher in dense and scoriaceous bombs than in inflated bombs produced in the same explosion, except in the latest two explosions in the sequence.

Average nucleation rates (*J*_ave_) also decreased over time in the period during which the first three explosions in the studied sequence occurred (August 2004–July 2006) (Fig. 9d). Nucleation rates then increased over time following this period. The uncertainty in nucleation rates for the latest explosion in the sequence is again relatively large, but nucleation rates are likely to be high as *N*_A_ is high in both samples from this explosion (Fig. 7a). The final nuclei population density *n*_0_ also decreased in the period during which the first three explosions occurred and increased thereafter (Fig. 9f). The initial decrease in nucleation rate coincided with a decrease in erupted volumes during the period August 2004–July 2006 and the subsequent increase in nucleation rates coincided with an increase in erupted volumes (Fig. 9b). In contrast, the initial decrease in growth rates during the period August 2004–January 2008 coincided with an increase in repose times between explosions and the subsequent slight decrease in growth rates coincided with decreasing repose times thereafter (Fig. 9a).

**Fig. 10:**
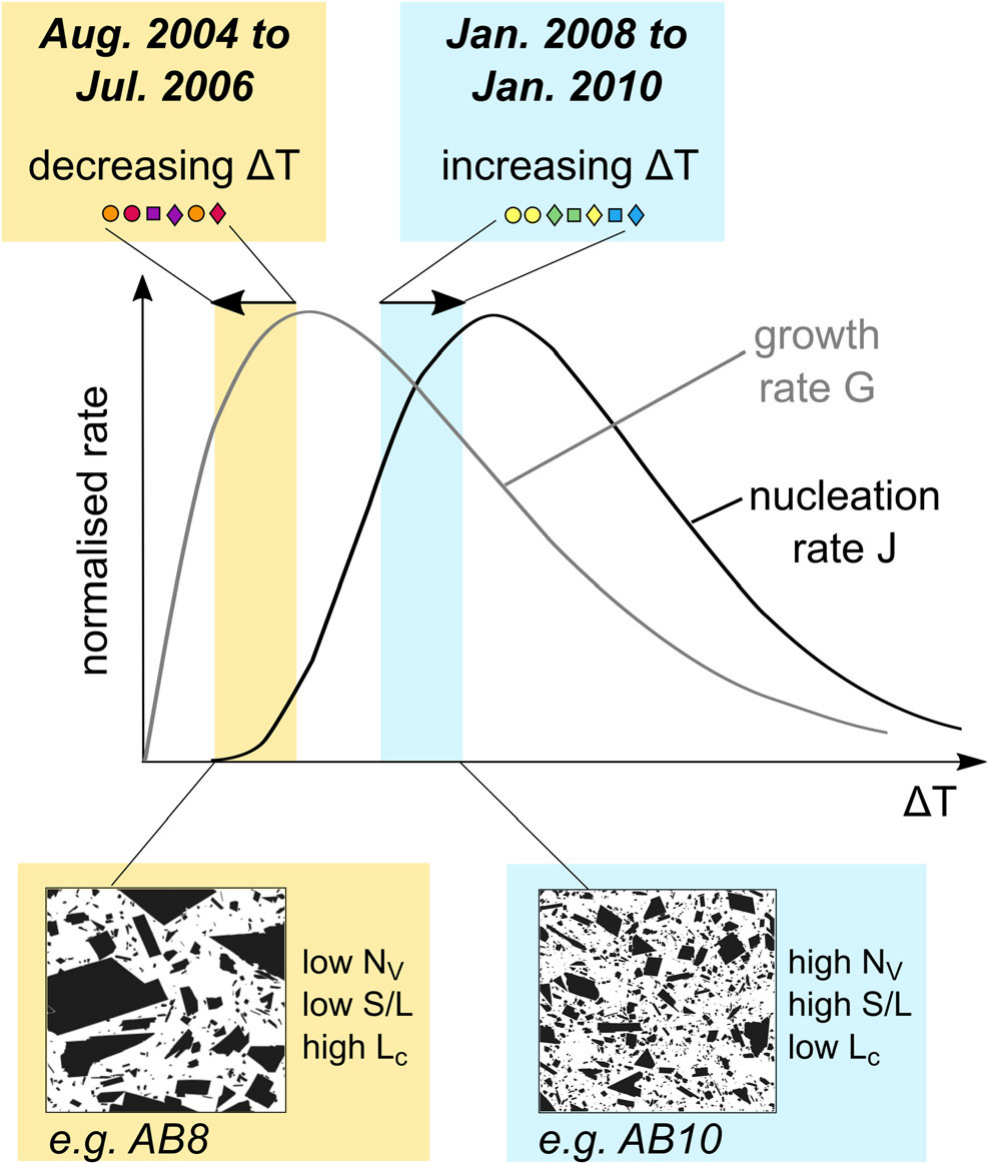
Schematic interpretation of the crystal micro-texture characteristics of Galeras samples in the context of evolving growth and nucleation rates with changing undercooling driven by degassing. Diagram adapted from Higgins (2006)

As previously noted by Zieg and Marsh (2002), the final nuclei population density (*n*_0_) and CSD slopes (*α*) correlate with the degree of effective undercooling (Δ*T*) in the melt phase (Online Resource 5, S6), and crystal growth and nucleation rates are a function of Δ*T* (Williams et al. 1954; Hammer and Rutherford 2002) (Fig. 10). The parameters *n*_0_, *G*_ave_ and *J*_ave_ therefore reflect the degree of undercooling in our samples. As *n*_0_, *G*_ave_ and *J*_ave_ all decreased during the period August 2004–July 2006 (Fig. 9c, d, f), Δ*T* must have decreased during this period (Fig. 10). In contrast, during the period January 2008–January 2010, *n*_0_ and *J*_ave_ increased and *G*_ave_ decreased slightly. The driving force for crystallisation (effective undercooling) Δ*T* therefore must have increased during this period (Fig. 10). A linear regression performed on the shallowest portion of each CSD confirmed that the patterns of variation in *n*_0_ over the eruption sequence are maintained in higher size bins (Online Resource 5, S7); therefore, these patterns affected all crystals in the groundmass population of these samples.

Inflated bombs typically show higher *n*_0_ and higher *J*_ave_ than dense and scoriaceous bombs produced in the same explosion (Fig. 9d, f). Sample AB22, the only inflated bomb with skeletal feldspar microlites (Online Resource 3), is distinct in showing a high *n*_0_ along with a relatively shallow CSD slope in the smallest size bins (Fig. 8 and Table 2). The final nuclei population density *n*_0_ is also related to batch textural parameters. As *n*_0_ increases, *N*_A_ and S/L increase (crystals become more tabular) and mean crystal areas decrease, and vice versa. The relationship between *n*_0_ and *ɸ* is less clear, similar to the relationship between *N*_A_ and *ɸ*, but the highest crystallinity samples tend to have low *n*_0_ and shallow CSD slopes. In order to compare two-dimensional textural parameters *N*_A_ and mean crystal area with three-dimensional textural parameters, the volumetric feldspar number density, *N*_V_, was calculated as *N*_V_ = ${n}_0\times \frac{-1}{\alpha }$ and a characteristic crystal size, *L*_c_, was calculated as *L*_c_ = $\frac{-1}{\alpha }$ (Blundy and Cashman 2008) (Table 2). Samples with low *N*_V_, corresponding to explosions that occurred in 2004–2006, have larger characteristic crystal sizes, *L*_c_, and more prismatic microlites whereas samples with high *N*_V_, corresponding to explosions that occurred in 2008–2010, have smaller characteristic crystal sizes and more tabular microlites (Fig. 7e, g). The relationship between *N*_V_ and *ɸ* is poorly defined (Fig. 7f). Comparing Fig. 7d, h shows that the complex relationship between mean crystal area and S/L reflects both the characteristic size of the microlites (microlites with large characteristic sizes result in a large mean crystal area, for example samples from the 12 July 2006 explosion) and the cut-section effect (the most likely intersection length of parallelepipeds such as prisms and tablets is close to the intermediate dimension I and the most likely intersection width is close to the short dimension S; therefore, intersections of more prismatic crystal shapes will result in smaller mean crystal areas even if their characteristic size is large, for example samples from the 2004 explosions). We conclude that the two-dimensional textural parameters adequately record the key textural information extracted from three-dimensional CSDs for this set of samples, despite the errors introduced by the intersection probability and cut-section effects. Figure 6b summarises the key textural characteristics of the different bomb types. All batch textural and CSD results are provided in Online Resource 7.

## Discussion

### Melt volatile content

Groundmass glass volatile analyses evince very low volatile contents in the interstitial melt of the magma plugs, with a higher volatile content in the magma that gave rise to inflated bombs than in the magma that gave rise to scoriaceous and dense bombs. The correlation of glass volatile content with bomb morphology and texture is consistent with the findings of Hoblitt and Harmon (1993) at Mount St Helens, who found that pyroclasts of 1980 blast dacite containing > 0.4 wt% H_2_O in the groundmass glass vesiculated on eruption timescales, whereas pyroclasts containing < 0.4 wt% H_2_O showed no evidence of syn-eruptive vesiculation and remained dense. They attributed this effect to the speciation of water in rhyolitic melt at very low water contents. Wright et al. (2007) also studied the H_2_O content of groundmass glasses in dense bombs and bread-crust bomb rinds from Guagua Pichincha volcano, Ecuador, and attributed the existence of dense bombs to a syn-eruptive bubble nucleation delay. Wright et al. (2007) suggested a higher threshold for syn-eruptive vesiculation to occur on eruptive timescales (0.9 wt% H_2_O), but noted that they found no dense bombs at > 0.4 wt% H_2_O. The morphological types of bombs found at Galeras volcano are therefore consistent with these interpretations. The pre-eruptive nature of vesicles in dense and scoriaceous bombs is also supported by the frequent presence of vapour-phase cristobalite growing on vesicle walls and the presence of tuffisite material infilling vesicles in scoriaceous bombs, as the growth of vapour-phase cristobalite occurs within magma plugs and domes (Horwell et al. 2013) and tuffisite veins form due to localised overpressure events within magma plugs and domes (Kendrick et al. 2016).

The only inflated bomb (AB28) with groundmass glass water content < 0.4 wt% H_2_O also features low CO_2_ content, the lowest F and Cl of all inflated bombs, elevated S and textural evidence of a cm-scale tuffisite vein in the rind (Online Resource 5, S5). The three dense bombs in which tuffisite veins were identified show similar volatile patterns, with low H_2_O (0.08–0.2 wt%) and CO_2_ (2–4 ppm), the lowest F (49–78 ppm) and Cl (63–87 ppm) contents in the sample suite and elevated S (11–16 ppm). These observations suggest an efficient, localised degassing mechanism associated with tuffisite veins, allowing H_2_O, CO_2_, F and Cl to become depleted in the interstitial melt surrounding the vein. In contrast, S is apparently re-absorbed into the adjacent melt, possibly due to increased oxygen fugacity and/or temperature (Wallace et al. 2015) and the advection of a S-rich gas phase through the tuffisite vein. As tuffisite veins locally increase permeability until sintering reduces it back to lower values (Heap et al. 2015), a tuffisite vein is likely to remain a preferential gas transfer pathway on a longer timescale than the short time required to create the tuffisite vein by brittle failure, enhancing the opportunity for localised re-equilibration of the melt to occur. The preservation of tuffisite material and this distinctive volatile signature in the rind of an inflated bomb (AB28) suggests that, while the localised degassing effect of tuffisite veins is significant, it remains a local effect that did not impede the ability of this parcel of magma to vesiculate upon eruptive decompression. Hence, the interstitial melt at a distance of a few millimetres from the tuffisite vein must have retained > 0.4 wt% H_2_O in order for the bomb to inflate. That the effect is localised is also supported by the absence of this signature in other inflated bombs that show preservation of tuffisite veins on a smaller scale, implying that larger-scale tuffisite veins affect a greater volume of adjacent interstitial melt. This is consistent with the findings of Castro et al. (2012), who investigated the effect of tuffisite veins on degassing rhyolitic magma erupted at Chaitén volcano, Chile, and concluded that their effect was limited. Castro et al. (2012) noted that the conditions where tuffisite veins may play a significant role in degassing magma on a large scale probably occur where they intersect existing porous, permeable networks. Tuffisite veins at Galeras volcano may therefore have contributed most to the overall degassing of the magma when they intersected the region of each magma plug that gave rise to scoriaceous bombs.

The only dense bomb that shows evidence of brittle deformation and grain comminution in the form of a cataclasite band (AB38) records a significantly higher CO_2_ content (60 ppm) in the groundmass glass of the adjacent homogeneous andesite. Cataclasite bands are thought to occur primarily at conduit margins and represent highly anisotropic high permeability zones channelling gas towards the surface (Gaunt et al. 2014). The cataclasite band in this dense bomb may therefore have acted as a permeable pathway for gas fluxing from deeper degassing magma towards the surface. The interstitial melt may have become enriched in CO_2_ as a result of this gas flux, as CO_2_ is soluble in water-poor melt at conduit pressures (Wright et al. 2007). Sulphur is also slightly enriched in this bomb sample (9 ppm), suggesting the flux of an S-bearing gas phase through the cataclasite zone.

These observations suggest that tuffisite veins act as degassing pathways advecting locally sourced, S-rich gas, whereas cataclasite veins act as pathways for deeper, more CO_2_-rich gas. The pre-eruptive porous network identified in scoriaceous bombs is likely to have provided permeable pathways that allowed relatively “volatile-rich” magma akin to that which gave rise to inflated bombs to become almost completely degassed. This degassed magma was the source of scoriaceous bombs, and porous network collapse may have allowed the densification of this magma, giving rise to dense bombs.

### Storage pressures and depth

**Fig. 11:**
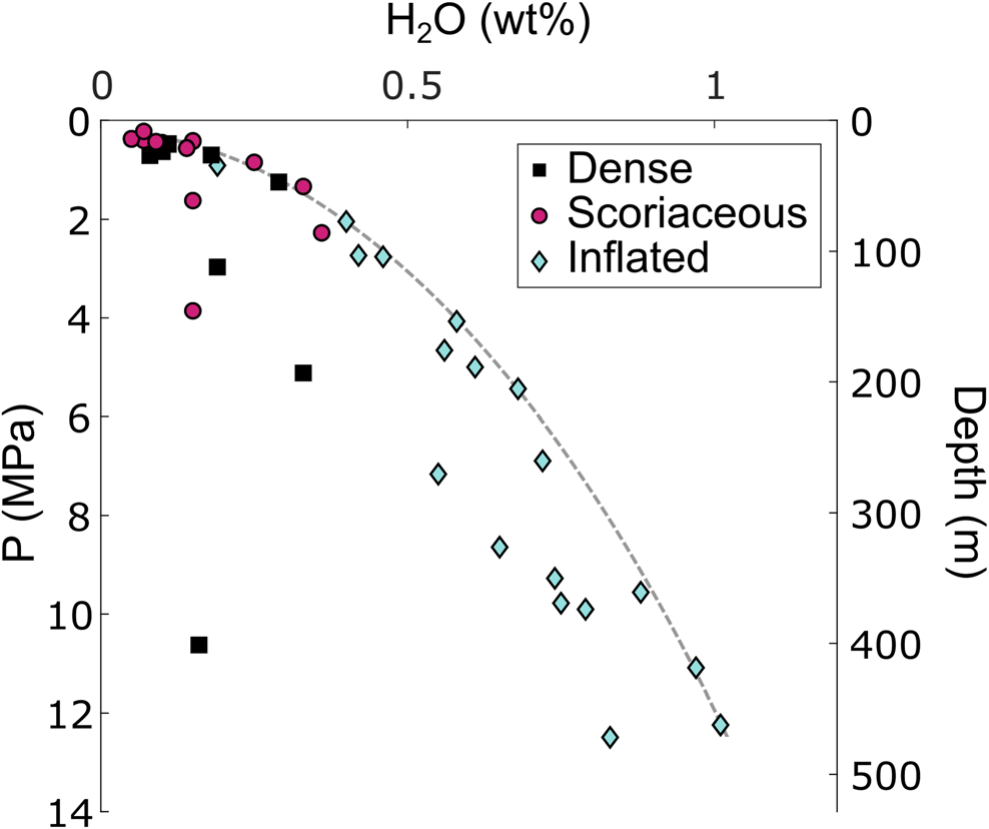
Minimum storage pressures and depths prior to vulcanian explosions from glass H_2_O and CO_2_ analyses using VolatileCalc (Newman and Lowenstern 2002). Each point represents one bomb sample. Samples that do not follow the overall degassing trend (grey dashed line) contain excess CO_2_ resulting in excessively high pressure estimates (see text)

The H_2_O and CO_2_ content of groundmass glasses were used in the solubility model of Newman and Lowenstern (2002) for rhyolitic melts to estimate the final storage pressure of each parcel of magma prior to explosion. The best temperature constraint available was obtained by two-pyroxene thermometry from products erupted during 2004–2006 (personal communication, G.P. Cortés, SGC, 21/10/2015). The temperatures indicated by touching pyroxene pair rims were higher than those indicated by the cores and averaged 980 °C. Assuming isothermal ascent and a temperature of 980 °C, the calculated storage pressures range from 0.2 to 12.5 MPa (Fig. 11). Estimates of storage pressure are excessively high for some samples containing excess CO_2_ interpreted to originate from vapour fluxing (Wright et al. 2007). These samples lie above the overall degassing trend fitted by eye in Fig. 11, but probably degassed along this trend with other samples prior to re-absorption of CO_2_.

The magma that gave rise to dense and scoriaceous bomb types was stored at the lowest pressures and over a similar range of pressures (Fig. 11): 0.2–3.9 MPa for scoriaceous bombs and 0.5–5.1 MPa for dense bombs (omitting sample AB38 that hosts the cataclasite vein). The magma that gave rise to inflated bombs was typically stored at higher pressures in the range 2–12.5 MPa (omitting sample AB28 that hosts a cm-scale tuffisite vein in the rind). Taking a magmatic temperature of 880 °C reduces these pressure estimates by 14.5–17%.

Assuming a uniform magma density of 2700 kg m^−3^, calculated storage depths are in the range 9–472 m (Fig. 11). Conversely, taking the maximum observed porosity for scoriaceous bombs (25%), assuming a uniform magma porosity and a gas density of 21 kg m^−3^ at 980 °C and 12 MPa (assuming pure water vapour and treating it as an ideal gas (Clarke et al. 2002)) yields a bulk density of 2030 kg m^−3^ and a range of depths of 11–627 m. As the uncertainty in magma density does not significantly affect our conclusions, for the purposes of the following discussion, we proceed with the depth estimates assuming a uniform magma density of 2700 kg m^−3^, which represent minimum storage depths.

Magma that gave rise to dense, scoriaceous and inflated bombs was stored in the ranges 18–193 m, 9–146 m and 77–472 m respectively. These results show that the magma that gave rise to inflated bombs upon eruption contained more dissolved water in the melt phase and was stored deeper than the magma that gave rise to contemporaneous dense and scoriaceous bombs. In addition, no more than approximately 500 m of stratified magma were ejected in any single explosion.

Storage pressures alone do not demonstrate whether the porous magma that gave rise to scoriaceous bombs underlay or overlay the degassed, dense magma that gave rise to dense bombs. However, most scoriaceous bombs record higher F and Cl content in the groundmass glass than most dense bombs, whereas dense bombs tend to record higher S and somewhat higher CO_2_ than scoriaceous bombs. In other words, the glasses in scoriaceous bombs contain higher concentrations of the volatile species that are expected to follow a degassing trend and that display this behaviour within the inflated bomb suite. In contrast, glasses in dense bombs contain higher concentrations of the volatile species that are expected to become re-dissolved in highly degassed magma at conduit pressures and that display this behaviour in samples containing remnant degassing pathways (tuffisite and cataclasite veins). Finally, the lower vesicularity of dense bombs, the polylobate shapes of vesicles in dense bombs when they are found and the gradual transition in vesicularity that exists between scoriaceous and dense bombs all support the idea that dense magma formed from densification of porous magma by viscous collapse of the porous network.

**Fig. 12:**
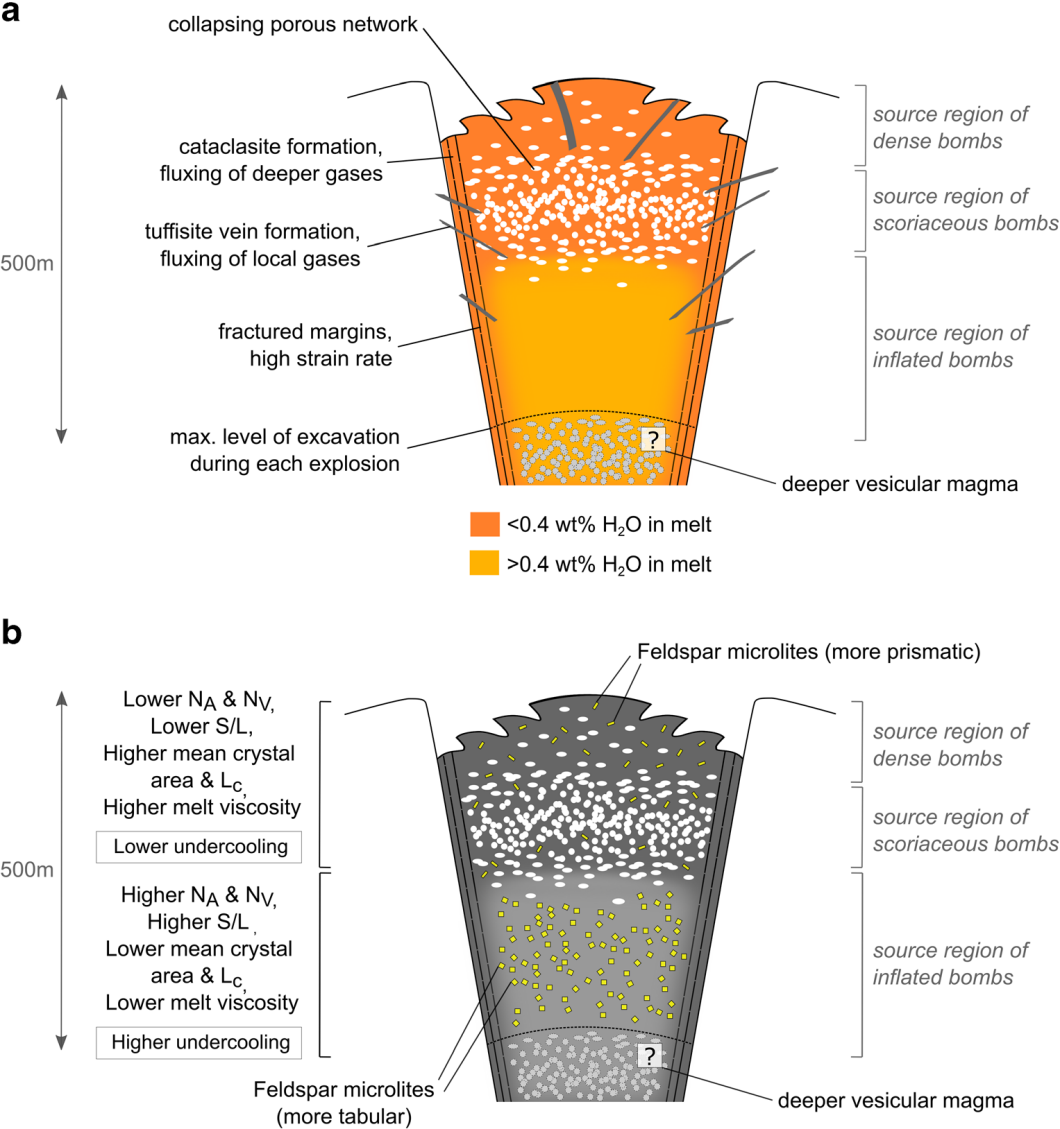
**a** Conceptual model of the typical magma plug architecture, with source regions for different bomb types. The degassed source region of dense/scoriaceous bombs has been enlarged to show detail, but extends to approximately 100 m depth (c.f. Fig. 11). Also shown is a hypothesised deeper vesicular magma that may have provided the source of overpressure needed to drive explosions. **b** Summary diagram of groundmass texture and melt viscosity characteristics of magma plugs at Galeras volcano. Phenocrysts not shown for clarity

The analyses of volatiles in groundmass glasses therefore support a conceptual model of repeated development and destruction of shallow, stratified plugs of magma in the conduit prior to vulcanian explosions. These volcanic plugs consisted of a deeper, dense and relatively water-rich magma that gave rise to inflated bombs, overlain by a largely degassed magma hosting a permeable, porous network that gave rise to scoriaceous bombs and capped by degassed, dense magma that gave rise to dense bombs (Fig. 12a). Prior to the onset of a vulcanian explosion, the permeable, porous network present in the porous magma had efficiently degassed the melt in the shallowest region of the plug and was in the process of viscously collapsing to form the dense magma cap. This process proceeded to various extents prior to individual explosions. This model implies that a pulse of vesiculation, bubble growth and coalescence occurred at a relatively consistent depth in the shallow conduit prior to each explosion. It also implies that the process of porous network closure and collapse in highly crystalline intermediate composition magmas is typically less efficient than the degassing process that the porous network effects, as pore pressures must have been decreasing for bubble collapse to occur.

The development of the overpressure that drove vulcanian explosions is therefore unlikely to have developed in the porous magma region that gave rise to scoriaceous bombs. The necessary gas overpressure is also unlikely to have developed in the magma that gave rise to inflated bombs, as this magma was dense (typically 0–0.5% vesicularity) rather than porous. It is most likely to have developed in a water-rich, porous zone underlying the magma that gave rise to inflated bombs, which was not preserved as ballistic bombs (Fig. 12a). Given the dense nature of inflated bomb rinds, these observations suggest that magma degassing during ascent to shallow levels was accomplished by multiple pulses of bubble nucleation, growth, coalescence and collapse, as deeper porous networks that degassed the magma from typical andesitic arc magma water contents (3–6 wt% H_2_O, Wallace et al. 2015) at depth to inflated bomb rind water contents (0.4–1 wt% H_2_O) must have collapsed prior to magma emplacement at the level of inflated bomb storage (< 500 m). The preservation of tuffisite veins in all bomb types suggests that this degassing mechanism was also operating and may have been a significant contributor over time. Given the anticipated impact of the development of porous permeable networks (accompanied by concomitant degassing and crystallisation) on magma buoyancy and rheology, the repeated development and collapse of porous networks is likely to be closely related to the occurrence of multiple cycles of magma ascent and stalling in the conduit, contributing to low average ascent rates.

### Melt compositions

Residual melt in the shallowest, most degassed region of each magma plug was generally more evolved than residual melt in the deeper, less degassed region. Assuming the magma that fed each plug was initially relatively compositionally homogeneous, the development of each stratified plug must have resulted from contrasting extents of crystallisation of the magma that gave rise to inflated and scoriaceous/dense bombs. Furthermore, variations from one explosion to the next imply that this process occurred repeatedly to form the sequential plugs but with slight variations over the course of the eruption sequence.

Glasses from the latest explosion for which time-constrained samples are available (2 January 2010) cover a more restricted range for all analysed oxides. Contrary to time-constrained samples from other explosions, these samples also feature increasing FeO*, TiO_2_ and Na_2_O wt% with increasing SiO_2_ wt%, and analyses of SiO_2_ in all samples from this explosion are among the highest in the sample suite (Fig. 4). Inflated bombs from this explosion are texturally unique in that the groundmass contains a higher proportion of glass and feldspar microlites are much smaller with skeletal morphologies. The unique compositional characteristics of the melt phase within this magma plug suggest nucleation-dominated crystallisation at higher degrees of Δ*T*. This implies that the magma involved in this explosion ascended at a faster rate and that a burst of crystal nucleation produced rapid evolution and degassing of the melt, resulting in magma arrest and rapid plug development in the shallow conduit. The repose time prior to this explosion was 43 days and is the shortest in the studied sequence.

**Fig. 13:**
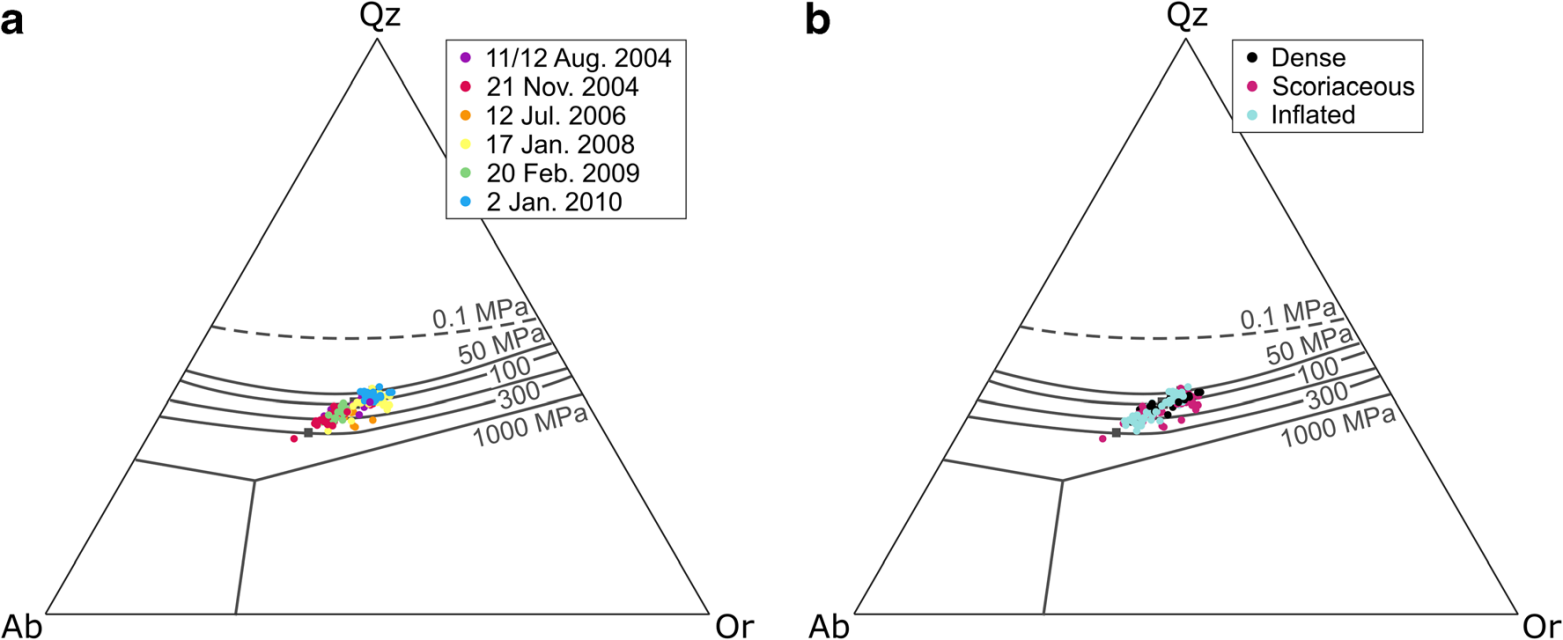
Glass analyses presented in the haplogranite ternary system (Cashman and Blundy 2000). Colours in **a** represent individual explosion dates, and colours in **b** represent bomb types

In the haplogranite ternary system, An-corrected normative Qz-Ab-Or compositions track the shift in the feldspar-quartz minimum with decreasing water pressure (Fig. 13) (Cashman and Blundy 2000). As the kinetics of plagioclase crystallisation are likely to be sluggish in high-viscosity, degassed rhyolitic melt due to low rates of diffusion, the high pressures indicated in the haplogranite ternary represent closure pressures where kinetic effects effectively limited further melt evolution and prevented the system from reaching equilibrium, rather than a true storage pressure (Cashman and Blundy 2000).

### Melt viscosity

Glass composition and volatile measurements were used to calculate the dynamic viscosity (*μ*) of the melt phase using the model of Giordano et al. (2008). Caution must be exercised with respect to the absolute values of viscosity as a result of the uncertainty in melt temperature. However, assuming that temperature gradients between the dense and volatile-rich portions of each plug are small, variations in viscosity may be used to assess the properties of the melt in the stratified magma plugs. Assuming a temperature of 980 °C, *μ* is in the range 10^5.9^–10^7.5^ Pa.s. *μ* is consistently lower for inflated bombs (10^5.9^–10^6.5^ Pa.s) than for dense and scoriaceous bombs (10^6.5^–10^7.5^ Pa.s), illustrating the compositional and volatile effects on melt viscosity. The degassed regions of the plugs therefore contained melt that was 1–1.5 log units more viscous than the more volatile-rich region of the plugs.

### Feldspar microlite compositions

As noted by Hammer et al. (2000) and Preece et al. (2016) at Merapi volcano, the compositions of feldspar microlites span a large range of An content. As plagioclase microlites evolved towards more sodic compositions, they record an apparent increase in temperatures with respect to isothermal sections of the dry ternary solvus at low pressures (Fig. 5c). Following Hammer et al. (2000) and Preece et al. (2016), we attribute this observation not to an actual increase in temperature but to the changing H_2_O activity in the melt in response to decompression, the resultant shift in the liquidus position and the compositional evolution of the melt phase due to ongoing crystallisation. Feldspar microlite compositions therefore act as a record of the extent of melt degassing and evolution prior to each explosion. At Galeras, they attest that the magma plug that was emplaced prior to the explosion of 17 January 2008 that experienced the longest repose time (554 days) produced the most “evolved” feldspar compositions, including the only observed alkali feldspar crystal. Unsurprisingly, these most evolved microlites were found in the more degassed scoriaceous bombs from that explosion rather than the contemporaneous inflated bombs (Fig. 5). However, in the sample suite as a whole, the lack of correlation between feldspar microlite composition and bomb type implies that the compositional range down to approximately An_30_ was achieved through degassing-driven crystallisation deeper in the conduit, during ascent prior to plug emplacement at the shallowest levels (< 500 m).

### Feldspar microlite textures

The final groundmass textures of volcanic rocks reflect the relative importance of microlite nucleation and growth rates (Williams et al. 1954; Hammer and Rutherford 2002), which are both functions of Δ*T* (Fig. 10). Effective undercooling Δ*T* in the context of ballistics produced by vulcanian explosions such as Galeras samples is controlled by melt degassing (rather than cooling), which depends on average decompression rate and decompression style (single-step or multiple-step decompression, Hammer and Rutherford 2002). Textures characterised by high *N*_A_ and low *ɸ* are generally thought to result from nucleation-dominated crystallisation prevailing during higher intensity vulcanian eruptions (higher Δ*T*), whereas textures characterised by lower *N*_A_ and higher *ɸ* are likely to result from growth-dominated crystallisation prevailing during lower intensity eruptions (lower Δ*T*) (Hammer et al. 2000; Brugger and Hammer 2010b; Preece et al. 2016). Feldspar crystal micro-textures in ballistic samples from Galeras volcano show systematic variations in batch textural parameters *N*_A_, mean crystal area and S/L over the course of the eruptive period (Fig. 7a–d), as well as variations in three-dimensional textural parameters *n*_0_, *N*_v_ and *L*_c_ (Figs. 7e–h and 9f–h), which must be driven by variations in Δ*T*. These textural variations coincided with systematic changes in repose time and ejected volume (Fig. 9), suggesting that variations in average ascent rate controlled the observed changes in ejected volume and repose time.

**Fig. 14:**
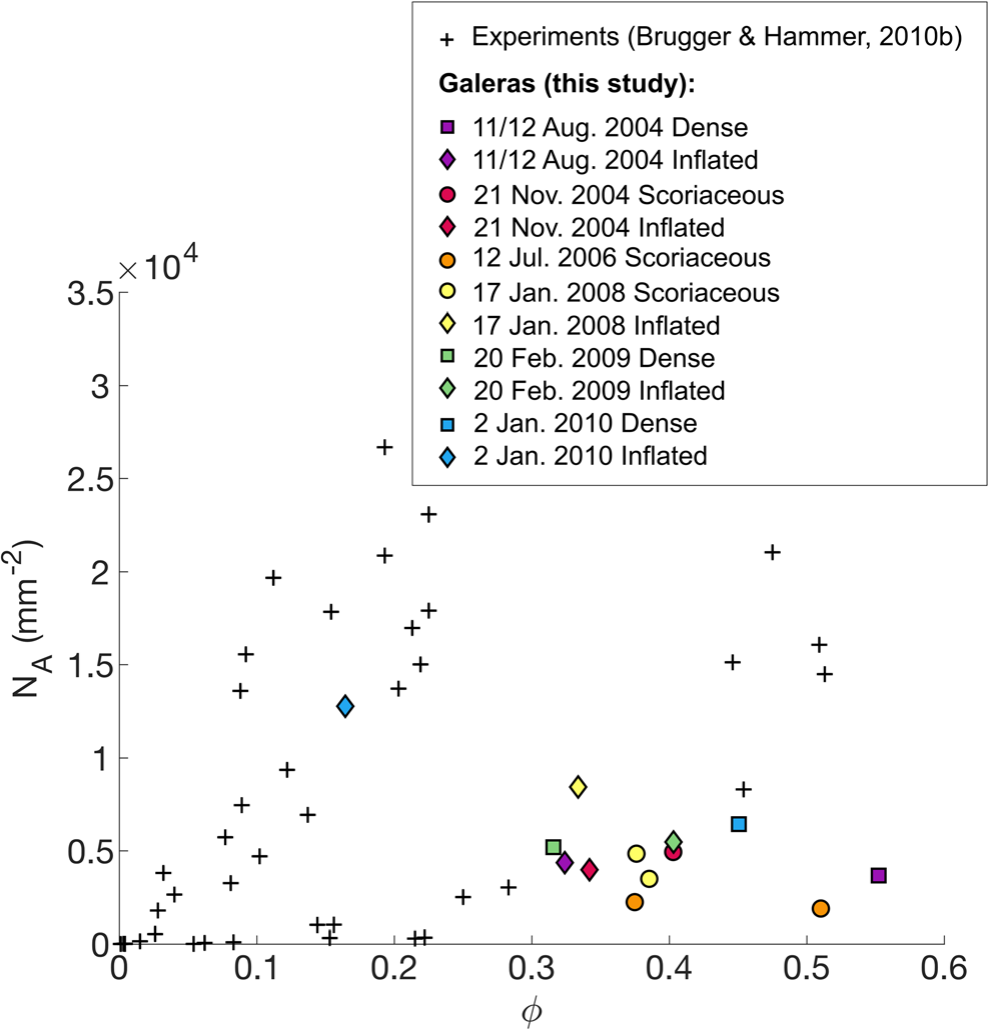
Comparison of Galeras feldspar microlite textural data with data from continuous decompression experiments by Brugger and Hammer (2010b)

We compare our textural results with the results of continuous isothermal decompression experiments conducted on hydrous rhyodacite by Brugger and Hammer (2010b) in order to estimate average magma decompression rates during this period. The decompressed samples of Brugger and Hammer (2010b) do not generally cover the same *N*_A_-*ɸ* space as most Galeras samples (Fig. 14). However, one experimental charge held at 880 °C and decompressed continuously at a rate of 1 MPa h^−1^ from 130 to 5 MPa, then held at the final pressure for 915 h (approx. 38 days) matches Galeras samples more closely (Fig. 14). Continuous decompression experiments approximate multi-step conditions with many small steps, relatively small degrees of Δ*T* at each step (compared to single-step decompression) and long-time steps between each decompression increment. This ascent path tends to produce melts that are far from equilibrium conditions and consequently record unrealistically high closure pressures, as observed in Galeras samples. The final annealing step reflects a possible stagnation period in a magma plug. The compact/euhedral (rather than skeletal) habit of crystals in most Galeras samples also supports slow decompression under modest degrees of Δ*T* (Lofgren 1974; Couch et al. 2003). The two continuous experiments that plot closest to sample AB22 along a line of constant mean crystal size through the origin were decompressed from 130 to 5 MPa and from 130 to 45 MPa respectively, at an average decompression rate of 10 MPa h^−1^ with no annealing time. This sample therefore appears to have experienced a decompression history that is distinct from other samples.

Inflated bombs typically show higher *N*_A_ and *N*_V_, smaller mean crystal area and *L*_c_ and lower *ɸ* than dense or scoriaceous samples produced in the same explosion (Fig. 7). This indicates that spatially variable degrees of Δ*T* existed within the stratified magma plugs and suggests that textural parameters may be used to compare the crystallisation regimes and degree of Δ*T* prevailing at different depths, as well as in plugs emplaced prior to different explosions. These textural trends indicate higher degrees of Δ*T* in the magma that gave rise to inflated bombs than in magma that gave rise to contemporaneous dense and scoriaceous bombs. This is in agreement with the higher extent of melt evolution in the degassed region of the plugs (Fig. 4), as high growth rates under moderate degrees of Δ*T* are expected to produce the most efficient crystallisation regime. High growth rates are also likely to result in crystals with higher aspect ratios (Lofgren 1974; Holness 2014), which are typically observed in dense and scoriaceous samples compared to contemporaneous inflated bombs (Fig. 9e). Textural trends also indicate higher degrees of Δ*T* overall in magma plugs emplaced from 2008 onwards, prior to the three latest explosions studied here. The average decompression rates estimated in this section suggest that an increase in average magma ascent rate corresponding to a shift from average decompression rates of 1–10 MPa h^−1^ was responsible for the changing Δ*T* and resulting crystallisation conditions.

The concave-upwards shapes of CSDs indicate slowing crystal growth rates prior to vulcanian explosions, leading to a steepening of the CSDs at small sizes. This could result from decreasing Δ*T* in each magma parcel prior to the explosions and associated microlite coarsening or from viscous limitation of crystal growth accompanying increasing Δ*T*. Decreasing Δ*T* could result from equilibrium being approached and a decrease in the driving force for crystallisation. However, the disagreement between the closure pressures indicated in the haplogranite ternary (Fig. 13) and the pressures indicated by the volatile contents of groundmass glasses imply that equilibrium was not reached. Rather, the increase in final nuclei population density *n*_0_ and steepening CSD slopes (Table 2, Online Resource 5) strongly argues for an increase in Δ*T* (Zieg and Marsh 2002) associated with slowing growth rates due to increasing melt viscosity. In general, higher Δ*T* is expressed texturally as higher *N*_A_ and *N*_V_ and more tabular microlites with lower mean crystal areas and *L*_c_ as a result of increasing nucleation rates and decreasing growth rates (Fig. 10). *ɸ* also tends to be lower under conditions of higher Δ*T* but *ɸ* is not the best criterion to evaluate Δ*T* as *ɸ* can increase under conditions of both high growth rates and high nucleation rates and thus is not linearly related to Δ*T*. This results in the scattered relationship between *ɸ* and other textural parameters (e.g. Fig. 7b, f).

Sample AB22 is distinct in that its CSD is only slightly concave and closer to a straight CSD with constant growth rate. This sample hosts large numbers of small skeletal microlites and has high *n*_0_ with a shallow CSD slope (Table 2). We interpret the CSD of this sample as reflecting a more rapid rate of ascent from the shallow magma storage region, with a more constant microlite growth rate over the timescale of ascent. This suggests a contrasting ascent path characterised by shorter stagnation periods during multi-step ascent in the conduit, producing a CSD that is closer to a straight CSD that might be expected from a continuous, steady ascent rate. This is consistent with the shorter repose periods between explosions prior to the eruption of this sample on 2 January 2010. We interpret the slight curvature of the CSD as a result of the short stagnation period prior to this explosion during which Δ*T* increased and growth rates decreased. This contrasts with the dense bomb ejected in the same explosion (AB21), which features a more curved CSD as a result of more prolonged multi-step ascent and a stagnation period in the most shallow, degassed part of the magma plug under lower degrees of Δ*T*.

*n*_0_ is higher and CSD slopes are steeper in samples from the latest three explosions studied here (17 January 2008, 20 February 2009, 02 January 2010), supporting the suggestion that Δ*T* was higher during this period than in samples from the earliest three explosions (11/12 August 2004, 21 November 2004, 12 July 2006) (Table 2). The volume ejected during an explosion generally increased as *n*_0_ in dense and scoriaceous bombs increased (Fig. 9b, f). The largest explosion (2 January 2010) ejected material with high *n*_0_ in both dense and inflated bombs, with a steep CSD slope in the dense bomb (AB21) and a relatively shallow CSD slope in the inflated bomb (AB22). This suggests that the largest explosions occur when there is a high nucleation rate throughout the plug under conditions of high Δ*T*, with a steep viscosity gradient in the interstitial melt. This viscosity gradient attests to the rapid formation of a densified plug at shallow levels with relatively low-crystallinity magma below. This could arise in the case of rapid gas loss from the top of the magma column following the previous explosion, followed by rapid, efficient sealing of the conduit. These conjectures are supported by the short repose time prior to this explosion.

Larger volume explosions typically produced textures characterised by lower S/L (more tabular crystals), lower mean crystal area and *L*_c_ and higher *N*_A_ and *N*_V_ (Fig. 9). Groundmass plagioclase crystallinity *ɸ* is more variable for the reasons mentioned previously, but larger explosions tend to eject inflated bombs with relatively low *ɸ* and dense/scoriaceous bombs with relatively high *ɸ*. Conversely, the smallest explosions are those characterised by modest *ɸ*, low *N*_A_ and *N*_V_, low S/L, high mean crystal area and *L*_c_ and low *n*_0_ with shallower CSD slopes. This implies that small explosions occur when Δ*T* is low (high growth rates prevail overall) and large explosions occur when Δ*T* is high (high nucleation rates prevail overall). Whereas ejected volumes vary with *n*_0_ in dense and scoriaceous bombs, repose times typically vary with *n*_0_ in inflated bombs (Fig. 9a, b, f). We interpret this as evidence that processes in the most degassed region of the magma plugs (e.g. densification) control the ejected volume whereas processes in the deeper, more volatile-rich region of the magma plugs (e.g. outgassing efficiency) control the repose time between explosions.

### Implications for magma decompression, conduit processes and eruption dynamics

The magma erupted during vulcanian explosions at Galeras volcano was decompressed in a step-wise fashion, at an average rate of 1 MPa h^−1^, accelerating to 10 MPa h^−1^ towards the end of 2009. Crystal nucleation began somewhere between a shallow crustal storage area and the level of shallow emplacement (< 500 m). Spatially variable crystallisation driven by differences in Δ*T* then proceeded within each magma plug. Crystal growth rates were typically rate limited by increasing residual melt viscosities under conditions of increasing Δ*T* prior to each explosion, leading to concave-upwards CSDs and increasing *n*_0_ over time. Higher volume explosions ejected material with a higher range of melt water content, interstitial melt viscosity, *ɸ*, mean crystal area, *N*_A_, *N*_V_ and *n*_0_, consistent with the ejection of magma that was stratified with respect to these properties.

The higher crystal nucleation rates and lower growth rates apparent in inflated bomb samples must reflect crystallisation conditions triggered by a final outgassing step that was greater in magnitude and produced a higher degree of Δ*T* than the outgassing experienced by scoriaceous and dense samples. To explain this, we invoke a vesiculation event that allowed a greater amount of degassing to occur in this region, followed by densification to produce the dense magma evinced by inflated bomb rinds (Fig. 12a, b). We envision that the magma arriving at the top of the Galeras conduit experiences multiple vesiculation events of different magnitudes on its path to the surface. These multiple vesiculation events accomplish the process of degassing the melt and create notably different final crystallisation regimes within 500 m of the surface that are recorded in feldspar microlite textures. We have direct evidence of one vesiculation event in the form of the pre-eruptive porous network preserved in scoriaceous bombs, and we hypothesise that the ash fraction produced by these vulcanian explosions may hold key information regarding vesiculation events that occurred at greater depth and that may represent the source of overpressure that eventually drove these explosions. Exceptions to the general pattern of higher Δ*T* in inflated bombs than in dense/scoriaceous bombs may be explained by lateral variations within the magma plugs or by highly variable ascent steps.

The overall higher rates of Δ*T* prevailing in the three latest explosions studied here likely result from a higher average magma ascent rate and hence a higher average decompression rate during this period. In the 2004–2006 period leading up to the extrusion of the first dome, repose times became longer and the volume ejected decreased, whereas in the 2008–2010 period, repose times became shorter and ejected volumes increased (Fig. 9a, b). We propose that magma degassing became more efficient throughout the first stage of the eruptive period, with accompanying longer repose times between explosions and smaller volumes ejected. This culminated in the extrusion of the first dome in January 2006. Following this, degassing became less efficient and repose times became shorter with larger amounts of material ejected in each explosion. The lack of dense bombs in the second and third explosions of the sequence (21 November 2004 and 12 July 2006) also supports the suggestion that the conduit was in a comparatively open state during this time. The fourth explosion in this study (17 January 2008) also lacked the presence of dense bombs, whereas the fifth and sixth explosions (20 February 2009 and 2 January 2010) produced dense bombs.

These observations suggest that variations in densification rate and magma ascent rate may be responsible for the shift in behaviour observed over the course of the eruption sequence. For example, a low densification rate during the early part of the eruptive period while magma ascent rates were also low could have allowed the porous network to remain open and the magma to successfully outgas, resulting in the extrusion of a dome and vulcanian explosions that became infrequent and smaller. No dense bombs were ejected, only scoriaceous and inflated bombs were produced. Conversely, higher densification rates in the latter part of the eruptive sequence while magma ascent rate was also high may have reduced degassing efficiency, leading to more frequent vulcanian explosions that ejected larger volumes, with a higher proportion of inflated bombs. Further investigation of this densification mechanism is beyond the scope of this paper, but we surmise that the systematic differences in crystal micro-textures resulting from variations in ascent rate may be responsible for rheological differences that control densification rate in the shallow conduit. For example, large, prismatic microlites that are characteristic of low ascent rates at Galeras may be expected to increase magma bulk viscosity by increasing particle-particle interactions (Mueller et al. 2011) and reduce densification rate, leading to more efficient outgassing. Conversely, small tabular microlites characteristic of higher ascent rates may produce a comparatively lower bulk viscosity that promotes rapid densification. This mechanism will be explored in future work. In terms of broader implications for vulcanian explosion dynamics and products at other arc volcanoes, we would expect small volume explosions (~ 10^5^ m^3^) to be generally associated with long repose times (hundreds of days) and produce mostly scoriaceous-type bombs, often in the presence of an extruded lava dome. Larger volume explosions (~ 10^6^ m^3^) may destroy existing lava domes and favour rapid plug formation and should be associated with generally short repose times (tens of days) and larger proportions of dense and inflated bombs.

Finally, the unique geochemical and textural characteristics of samples from the explosion that occurred on 2 January 2010 suggest that it represents a different type of explosion related to higher ascent rates that should be considered separately from the others at Galeras and possibly represents a regime shift that contributed to ending the eruptive sequence. This implies that results from the typical explosions of Galeras volcano may not be transferable to all types of vulcanian explosions at other volcanoes if different regimes exist. For example, the lack of pumice clasts produced by explosions at Galeras volcano contrasts with the presence of these products in vulcanian explosions at other volcanoes (e.g. Soufrière Hills, Clarke et al. 2007; Giachetti et al. 2010). The reason for this is not clear and may be related to syn-eruptive processes that might be recorded in the interiors of inflated bombs or in the ash fraction of erupted products. We suggest that the different regimes of vulcanian activity need to be carefully identified to build further understanding of this eruption style.

## Conclusions

Our textural and geochemical constraints on a rare set of time-constrained, texturally diverse ballistic samples from Galeras volcano suggest that similar, vertically stratified magma plugs developed prior to vulcanian explosions. Second-order variations between plugs are associated with evolving eruption dynamics throughout the 2004–2010 sequence of vulcanian explosions. The main conclusions from our study are as follows:

- Plugs were stratified with respect to melt volatile content, melt composition, melt viscosity, feldspar microlite characteristics and vesicularity.
- Degassing-driven effective undercooling was increasing and viscosity-limited crystallisation was ongoing in all regions of the plugs prior to each explosion.
- During 2004–2008, shallow conduit conditions became progressively more open for degassing, with explosions becoming smaller and less frequent, culminating in the construction of two lava domes.
- During 2008–2010, the shallow conduit became more closed for degassing as a result of increased ascent rates, and explosions became more frequent and larger.
- The region where pore overpressure increased sufficiently to initiate vulcanian explosions was not preserved as ballistics, suggesting that the ash fraction of eruption products may hold key additional information.

Finally, this study of time-constrained samples suggests that variable magma ascent rates produce differences in plug densification rate and gas permeability, possibly through variations in crystal micro-textures that impact magma rheology. These variations in magma densification rate and permeability merit further study and may represent key processes in explaining the observed variations in magnitude and timing of vulcanian explosions at Galeras and other arc volcanoes.

## Electronic supplementary material

Online Resource 1 Full SIMS methods (PDF 416 kb)

Online Resource 2 EPMA analyses of groundmass glass and feldspar microlites (XLSX 55 kb)

Online Resource 3 BSE images and feldspar microlite tracings used for textural analysis (PDF 65497 kb)

Online Resource 4 Photo-micrographs of bomb sample textures (PDF 182693 kb)

Online Resource 5 Supplemental figures (PDF 4.62 MB)

Online Resource 6 SIMS analyses of groundmass glass volatiles (XLSX 132 kb)

Online Resource 7 Textural (batch and CSD) and melt viscosity results (XLSX 86 kb)
