# Supplementary material for: Textural and geochemical constraints on andesitic plug emplacement prior to the 2004–2010 vulcanian explosions at Galeras volcano, Colombia
Source: Bull Volcanol. 2018 Dec 7;81(1):1. doi: 10.1007/s00445-018-1260-y (PMC6383983; doi:10.1007/s00445-018-1260-y)
Supplement: Supplementary file 1 — Full SIMS methods (PDF 416 kb) [file 445_2018_1260_MOESM1_ESM.pdf]

Online Resource 1: Full SIMS Methods

Textural and geochemical constraints on andesitic plug emplacement prior to the 2004-2010 vulcanian explosions at Galeras volcano, Colombia, Bulletin of Volcanology

Amelia A. Bain[1]*, Eliza S. Calder[1], Joaquín A. Cortés[2], Gloria Patricia Cortés J.[3], Susan C. Loughlin[4]

[1]Grant Institute, The University of Edinburgh, Edinburgh, UK

[2]Department of Geography, Edge Hill University, Ormskirk, UK

[3]Servicio Geológico Colombiano, Observatorio de Manizales, Manizales, Colombia

[4]The Lyell Centre, British Geological Survey, UK

*Corresponding author: Amelia.Bain@ed.ac.uk

Sample preparation

Samples were selected from the groundmass of dense bombs, scoriaceous bombs and the rapidly quenched rinds of inflated bombs. The highly vesicular interiors of inflated bombs were excluded from analyses due to the likelihood of syn-eruptive volatile loss from the melt. Due to the overall textural homogeneity of dense and scoriaceous bombs, samples were taken from any part of these samples. To sample the rinds of inflated bombs, cores were drilled into the dense, quenched rind. In the case of inflated bombs with relatively thin rinds, the cores were then ground down from the interior side to remove as much lighter-coloured vesicular glass as possible, in order to eliminate any material likely to have suffered syn-eruptive volatile loss.

Once samples were obtained from each bomb in this way, each sample was crushed with a hammer to produce grains 0.5-1mm in diameter. Eight glassy grains from each sample were selected and mounted in epoxy blocks. Grains were clearly separated from each other by dividers and labelled by sample number. Epoxy blocks were then ground and double polished in order to intersect all grains. Blocks were gold-coated in preparation for ion microprobe analysis.

Analyses

Goundmass glass volatile contents were measured by secondary ion mass spectrometry (SIMS) at the EIMF (Edinburgh Ion Microprobe Facility) using a Cameca 1270 instrument, with an oxygen source in the form of a focussed beam of O^{2-} ions at 12keV. After pre-rastering a larger area to sputter off any surface contamination, the incident ion beam diameter was 10-

15µm in diameter and ejected secondary ions from a volume of glass on the scale of that beam.

Species analysed included H, OH, C, S, Cl and Fl. Also included were Si, Mg and Ca, in order to judge whether any crystals were intersected below the surface during the analysis and may have diluted the volatiles signal from the interstitial glass, due to a partial volume effect. This approach was taken in order to counteract the known difficulty associated with analysing small areas of glass in highly-crystalline samples. Secondary ion counts were recorded using an electron multiplier.

Five to seven spots were analysed on the groundmass glass of one or more grains belonging to each ballistic bomb, taking care to choose areas that were the most crystal free and away from vesicles, cracks or sample edges in order to avoid contamination from polishing residue. Where insufficient high-quality data was collected due to matrix effects dominating the volatiles signal or due to intersection of crystals, point analyses were checked and others carried out on the Cameca 4f instrument.

Secondary ion counts for the elements of interest were corrected for background counts, normalised by the Si counts and calibrated using the known compositions of a variety of rhyolitic glass standards. Separate calibrations were performed for results from the 1270 and 4f instruments. A correction of $8*10^{-9}$cps (counts per second) was applied to correct for the potential mass overlap of CaMg compounds with S.

The Cameca 1270 instrument used for the majority of analyses collected 15 cycles of measurements of each species of interest. The first 7 cycles were discarded as per the usual practice, in order to avoid surface contamination. Due to the difficulty of sampling very small areas of glass in very crystalline samples, Mg and Ca counts were monitored through the remaining 8 cycles in order to assess whether the material sputtered off by the incident beam was likely to have changed from groundmass glass to a crystal phase such as plagioclase or pyroxene. Accordingly, analyses were flagged for further inspection where there was a change of more than 50% in Mg or Ca counts through those last 8 cycles. When analyses were flagged in this way, in cases where Mg and Ca counts remained low in the first 3 cycles of the last 8 under consideration, the average of those 3 cycles were taken as an acceptable analysis. Analyses were also discarded if C, Mg and/or Ca were notably higher than other analyses performed on the same sample, due to the likelihood of intersecting crystals, cracks or vesicles below the surface.

Errors on the unknowns were calculated from the repeatability on the standards using the Cameca 1270 instrument and a background correction. 2 sigma confidence intervals for H_2O were in the range 0.016-0.169 wt%, for CO_2 in the range 2-7 ppm, for S in the range 0.7-1.4 ppm, for Cl in the range 15-233 ppm and F in the range 6-116 ppm.
