# Supplementary material for: Textural and geochemical constraints on andesitic plug emplacement prior to the 2004–2010 vulcanian explosions at Galeras volcano, Colombia
Source: Bull Volcanol. 2018 Dec 7;81(1):1. doi: 10.1007/s00445-018-1260-y (PMC6383983; doi:10.1007/s00445-018-1260-y)
Supplement: Supplementary file 3 — BSE images and feldspar microlite tracings used for textural analysis (PDF 65497 kb) [file 445_2018_1260_MOESM3_ESM.pdf]

Online Resource 3: Image Analysis

Textural and geochemical constraints on andesitic plug emplacement prior to the 2004-2010 vulcanian explosions at Galeras volcano, Colombia

Bulletin of Volcanology

Amelia A. Bain, Eliza S. Calder, Joaquín A. Cortés, Gloria Patricia Cortés J., Susan C. Loughlin

This online resource provides BSE (back-scattered electron) images of the groundmass of each sample used for textural analysis, along with the corresponding tracings of feldspar microlites. Areas analysed are indicated by the yellow polygon in each image. The area analysed, image length and number of whole crystals in the analysed area are indicated below each image. Examples of features of interest are labelled in yellow:

Pl = plagioclase
Px = pyroxene
Fe-Ti = Fe-Ti oxide
Gl = glass
V = vesicle

AB2 BSE image

Dense bomb

Explosion date: 11-12 August 2004

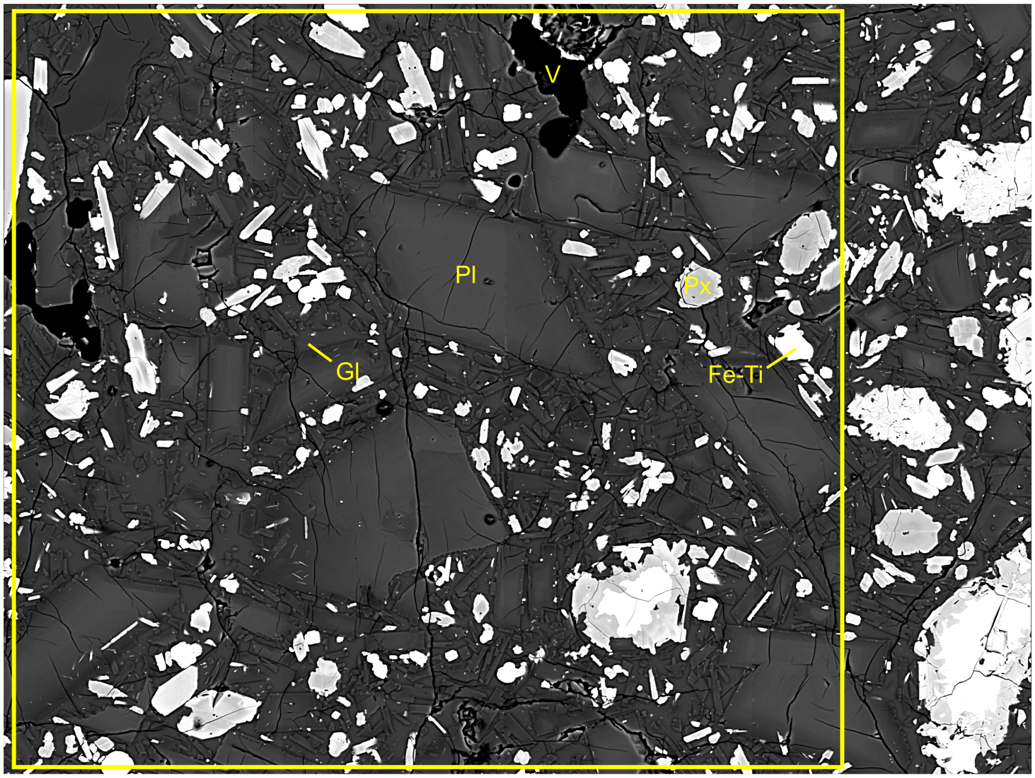

Sample: AB2

Image length: 0.754 mm

Analysed area: 0.348 mm^2

Number of feldspar crystals in analysed area (N): 1217

AB2 Feldspar microlites

Dense bomb

Explosion date: 11-12 August 2004

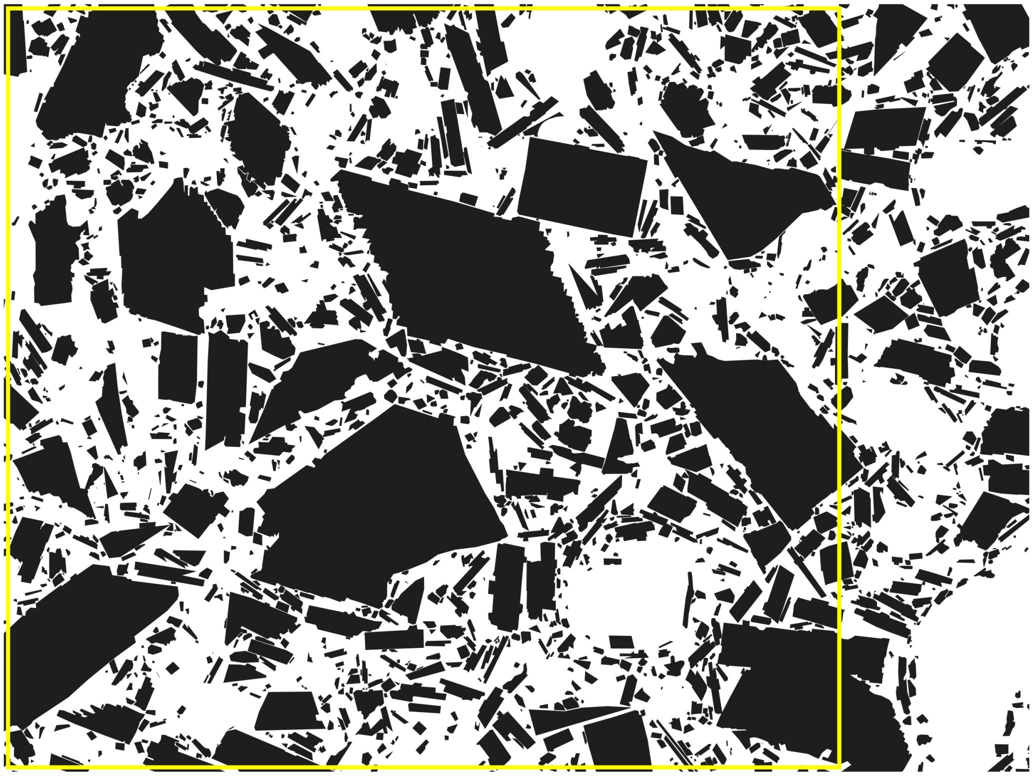

Sample: AB2

Image length: 0.754 mm

Analysed area: 0.348 mm^2

Number of feldspar crystals in analysed area (N): 1217

AB4 BSE image

Inflated bomb rind

Explosion date: 11-12 August 2004

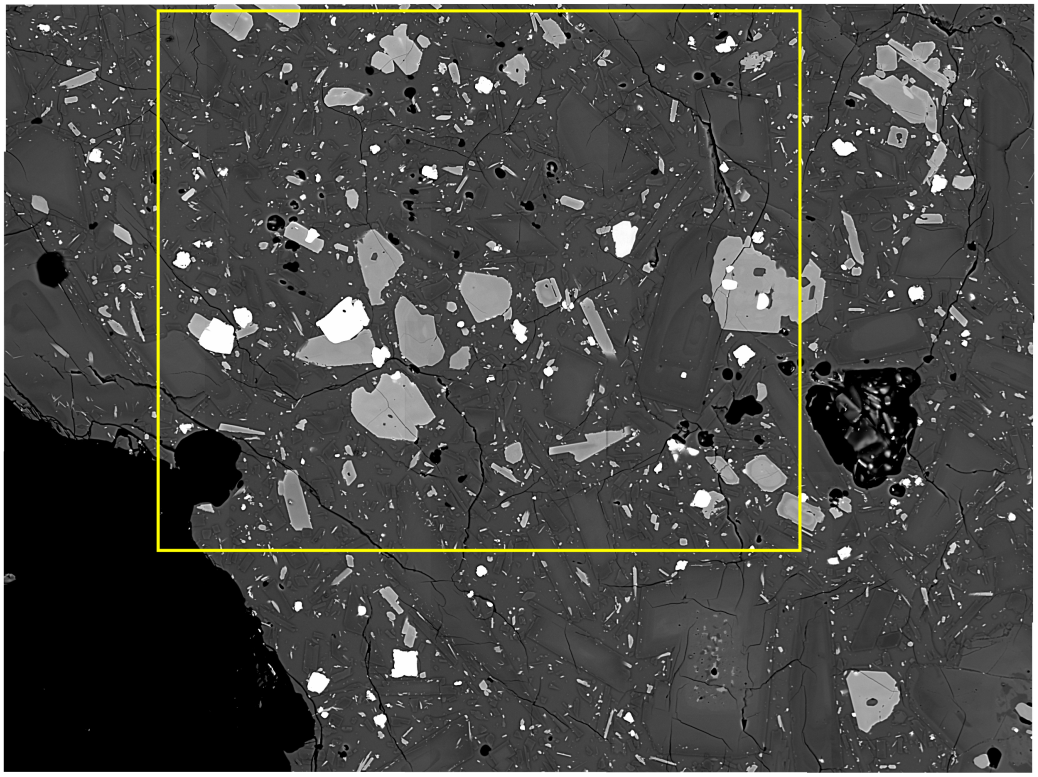

Sample: AB4

Image length: 0.959 mm

Analysed area: 0.338 mm^2

Number of feldspar crystals in analysed area (N): 1391

AB4 Feldspar microlites

Inflated bomb rind

Explosion date: 11-12 August 2004

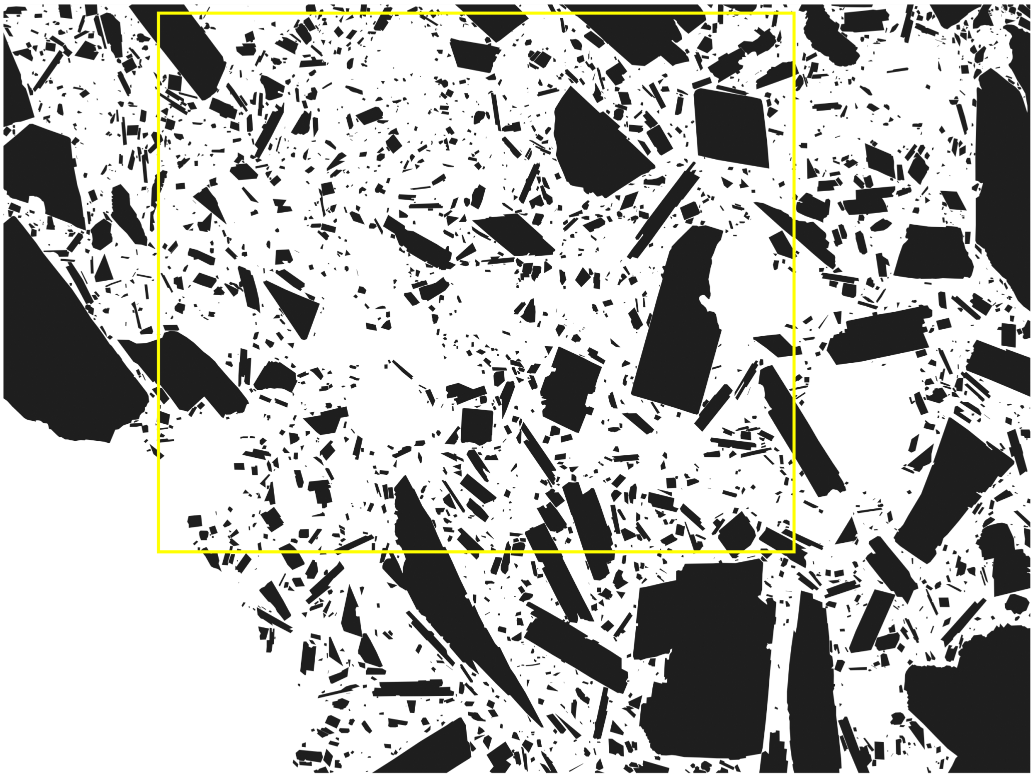

Sample: AB4

Image length: 0.959 mm

Analysed area: 0.338 mm^2

Number of feldspar crystals in analysed area (N): 1391

AB5b BSE image

Scoriaceous bomb

Explosion date: 21 November 2004

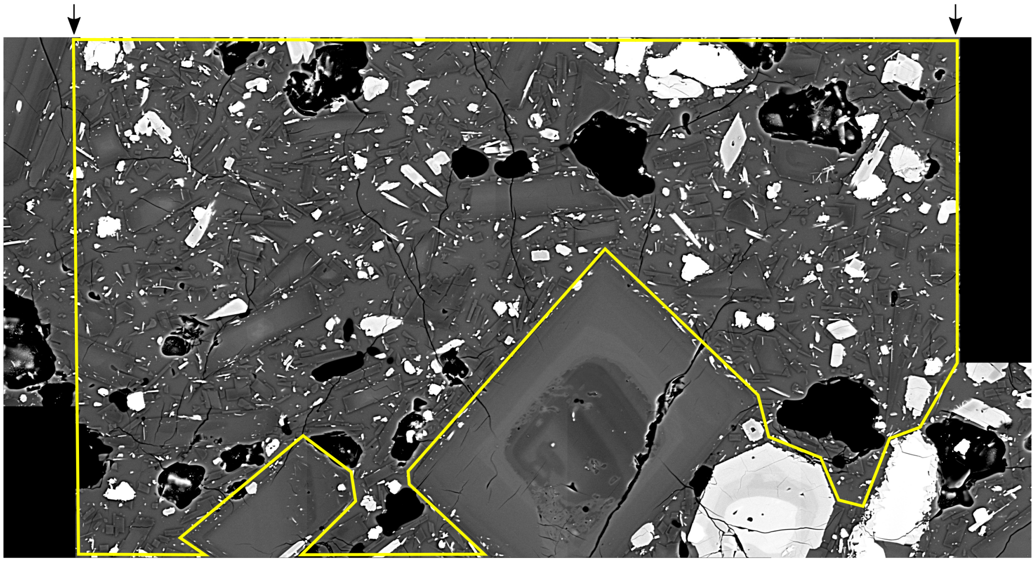

Sample: AB5b

Image length (between arrows): 0.711 mm

Analysed area: 0.228 mm^2

Number of feldspar crystals in analysed area (N): 1001

AB5b Feldspar microlites
Scoriaceous bomb
Explosion date: 21 November 2004

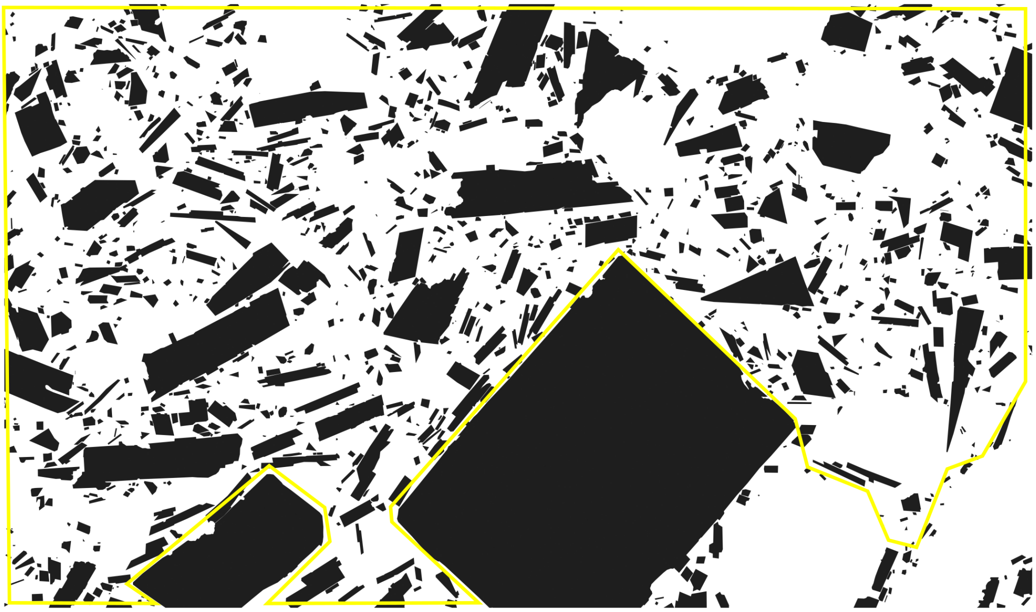

Sample: AB5b
Image length: 0.711 mm
Analysed area: 0.228 mm^2
Number of feldspar crystals in analysed area (N): 1001

AB6 BSE image
Inflated bomb rind
Explosion date: 21 November 2004

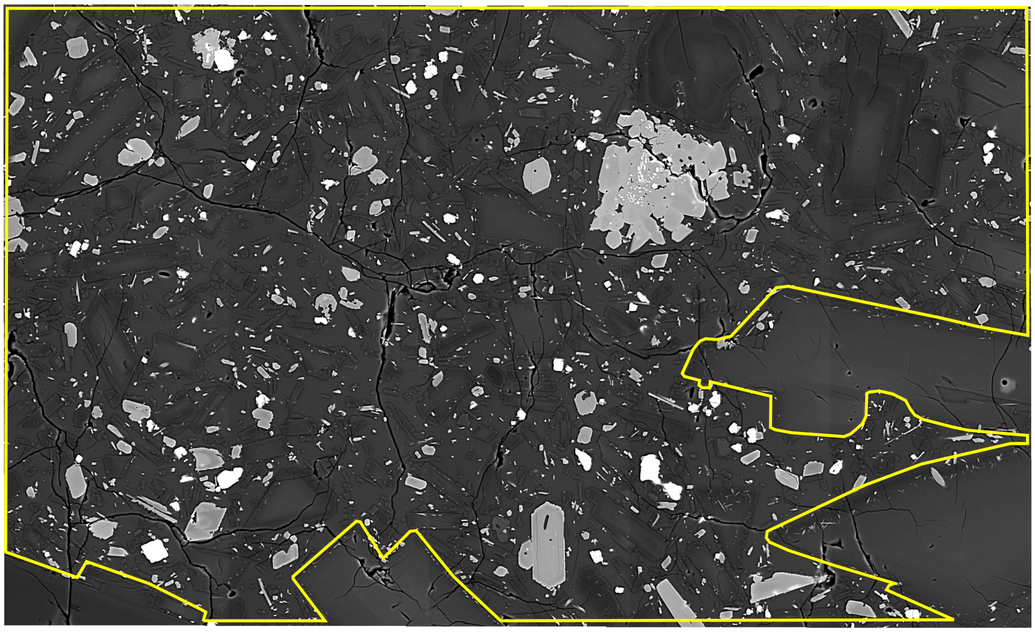

Sample: AB6
Image length: 0.948 mm
Analysed area: 0.464 mm^2
Number of feldspar crystals in analysed area (N): 1818

AB6 Feldspar microlites
Inflated bomb rind
Explosion date: 21 November 2004

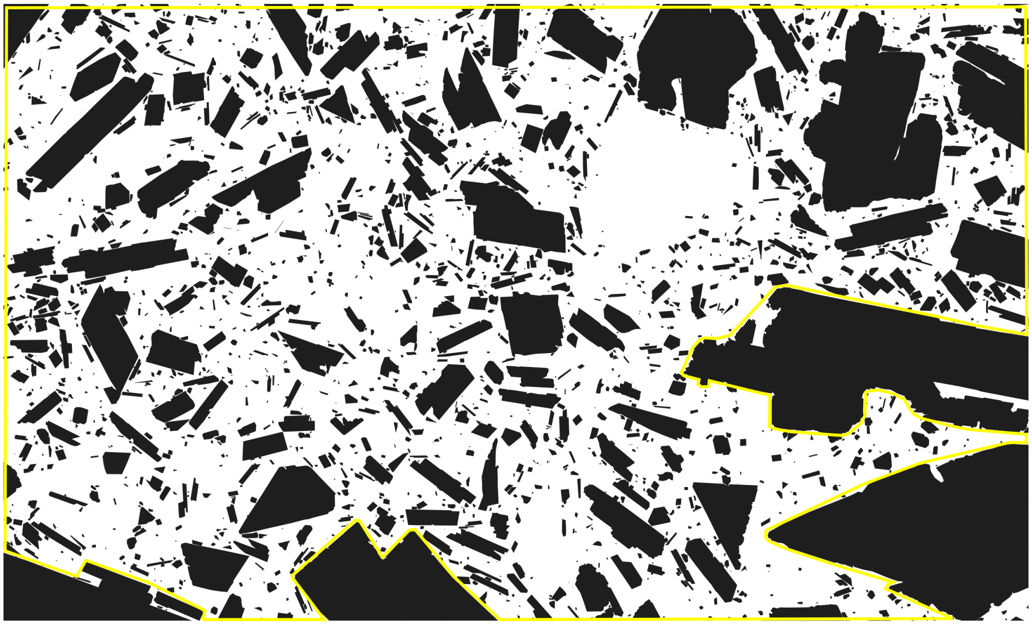

Sample: AB6
Image length: 0.948 mm
Analysed area: 0.464 mm^2
Number of feldspar crystals in analysed area (N): 1818

AB8 BSE image

Scoriaceous bomb

Explosion date: 12 July 2006

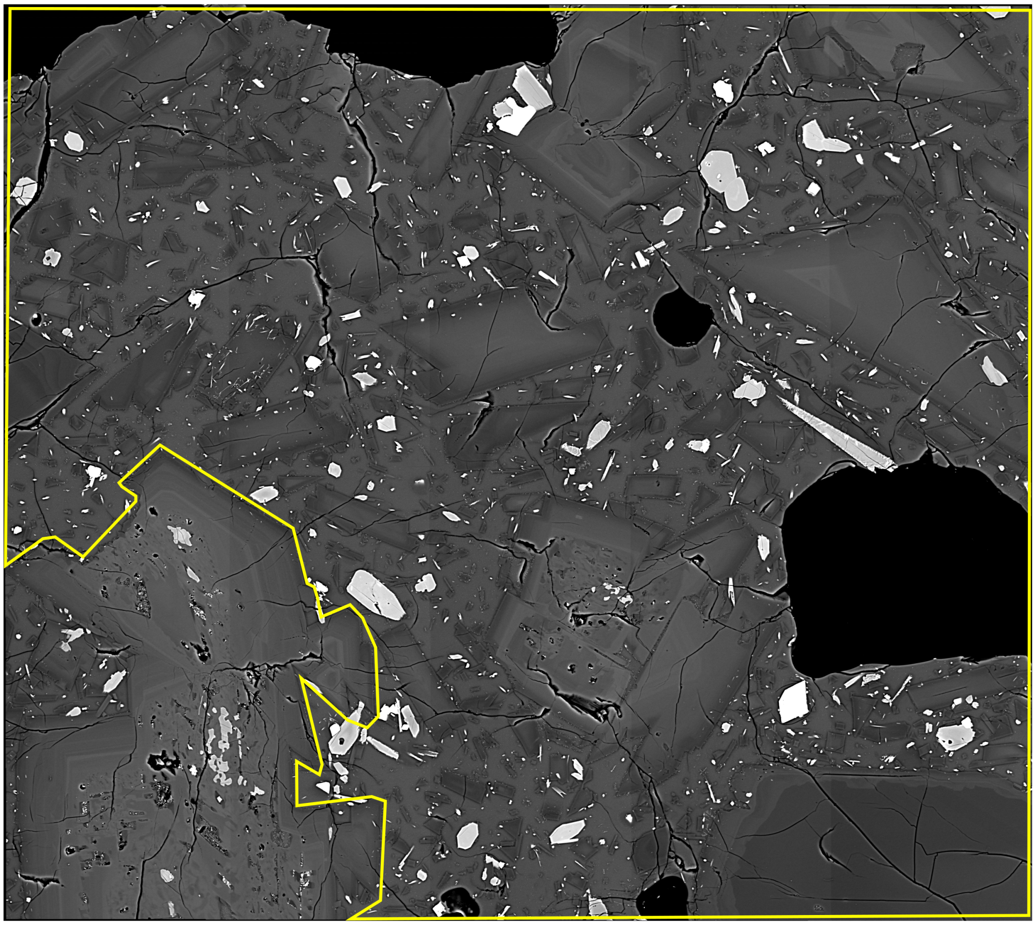

Sample: AB8

Image length: 0.939 mm

Analysed area: 0.649 mm^2

Number of feldspar crystals in analysed area (N): 1080

AB8 Feldspar microlites
Scoriaceous bomb
Explosion date: 12 July 2006

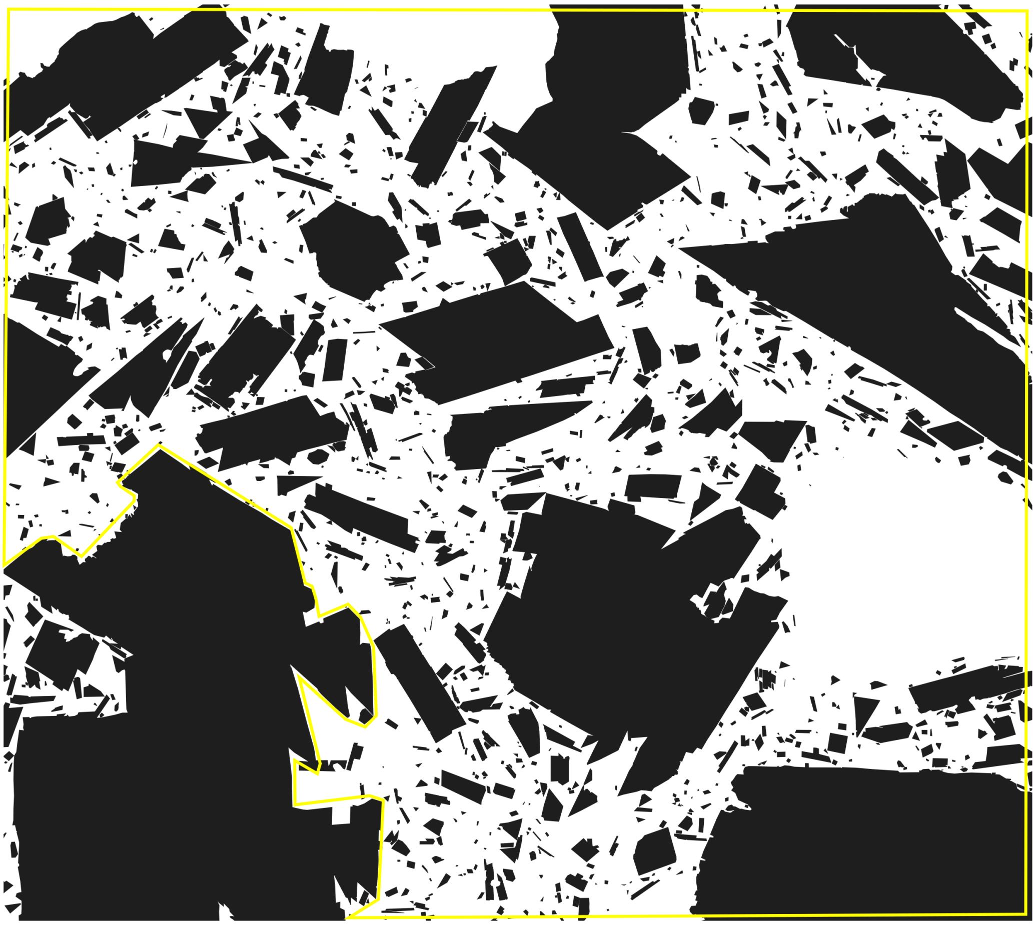

Sample: AB8
Image length: 0.939 mm
Analysed area: 0.649 mm^2
Number of feldspar crystals in analysed area (N): 1080

AB9 BSE image

Scoriaceous bomb

Explosion date: 12 July 2006

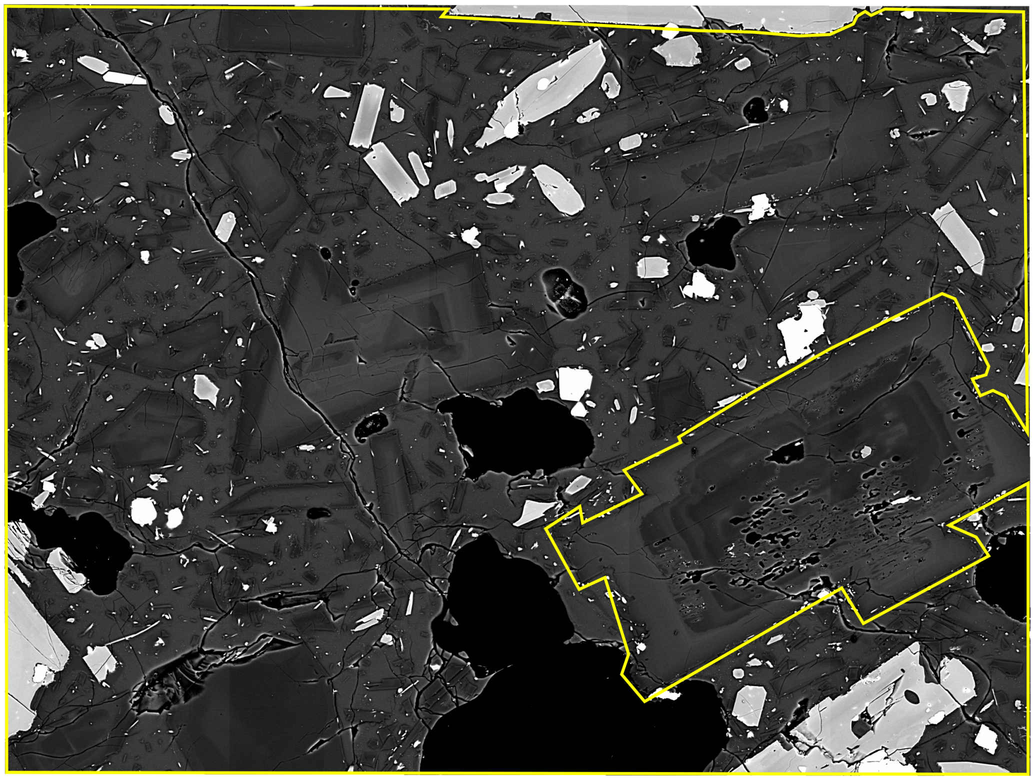

Sample: AB9

Image length: 0.948 mm

Analysed area: 0.577 mm^2

Number of feldspar crystals in analysed area (N): 1100

AB9 Feldspar microlites
Scoriaceous bomb
Explosion date: 12 July 2006

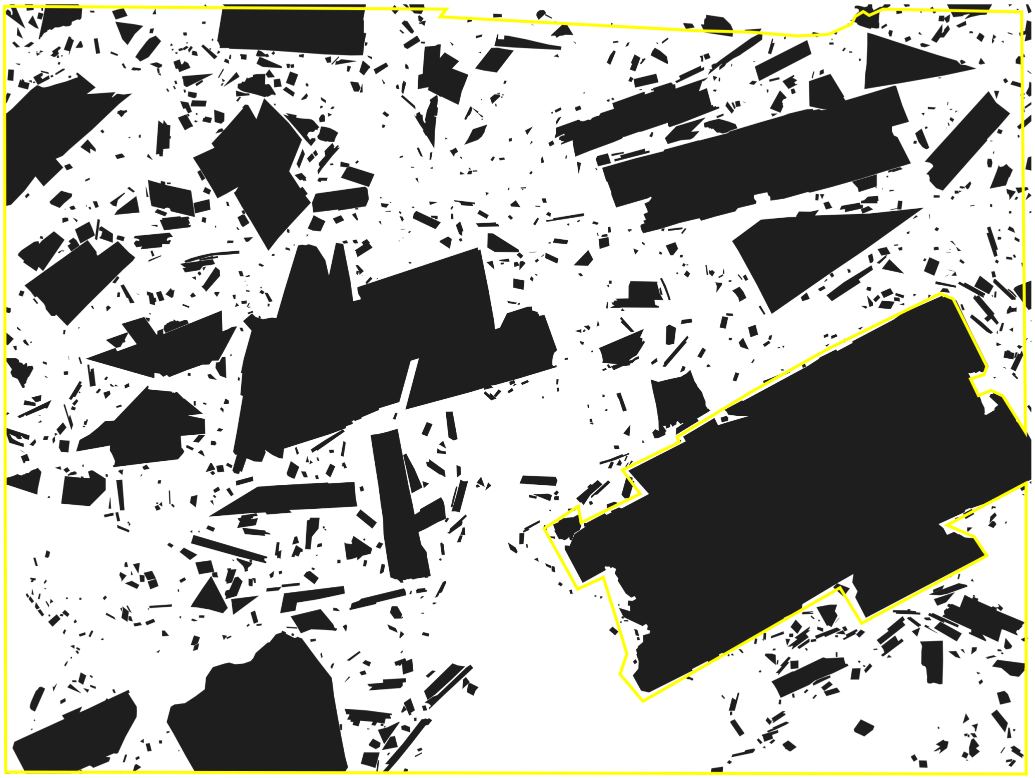

Sample: AB9
Image length: 0.948 mm
Analysed area: 0.577 mm^2
Number of feldspar crystals in analysed area (N): 1100

AB10 BSE image
Inflated bomb rind
Explosion date: 17 January 2008

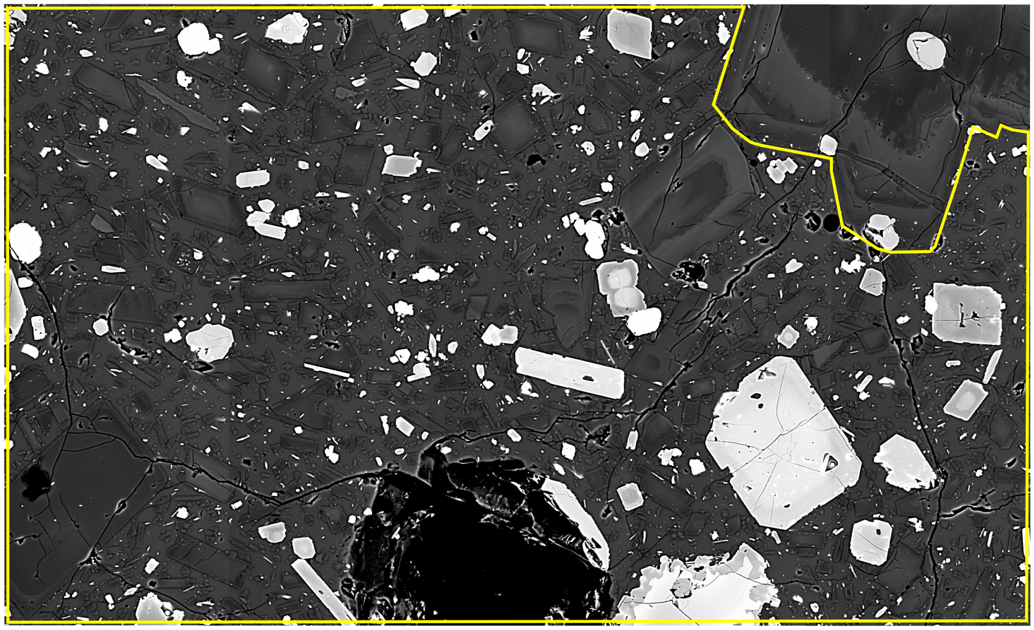

Sample: AB10
Image length: 0.948 mm
Analysed area: 0.494 mm^2
Number of feldspar crystals in analysed area (N): 3661

AB10 Feldspar microlites
Inflated bomb rind
Explosion date: 17 January 2008

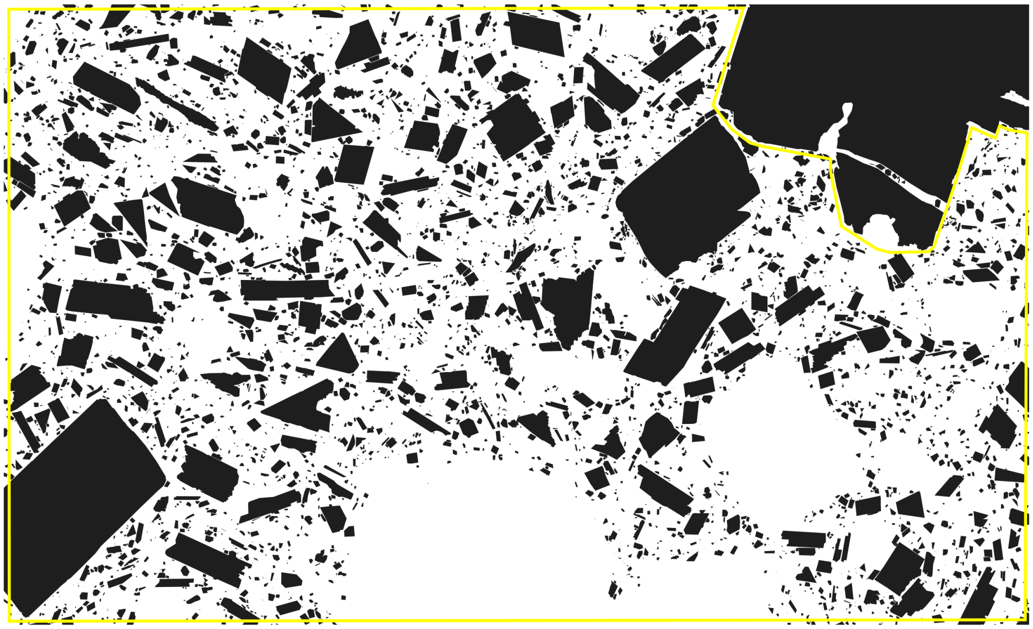

Sample: AB10
Image length: 0.948 mm
Analysed area: 0.494 mm^2
Number of feldspar crystals in analysed area (N): 3661

AB14 BSE image
Scoriaceous bomb
Explosion date: 17 January 2008

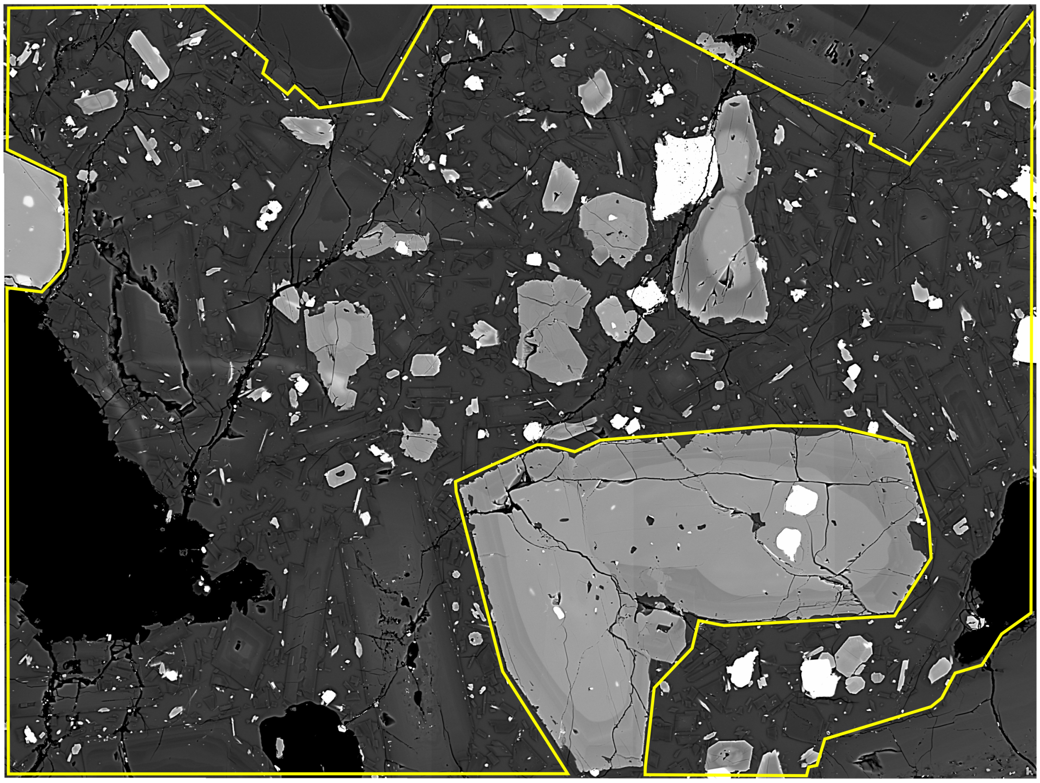

Sample: AB14
Image length: 0.957 mm
Analysed area: 0.505 mm^2
Number of feldspar crystals in analysed area (N): 1443

AB14 Feldspar microlites

Scoriaceous bomb

Explosion date: 17 January 2008

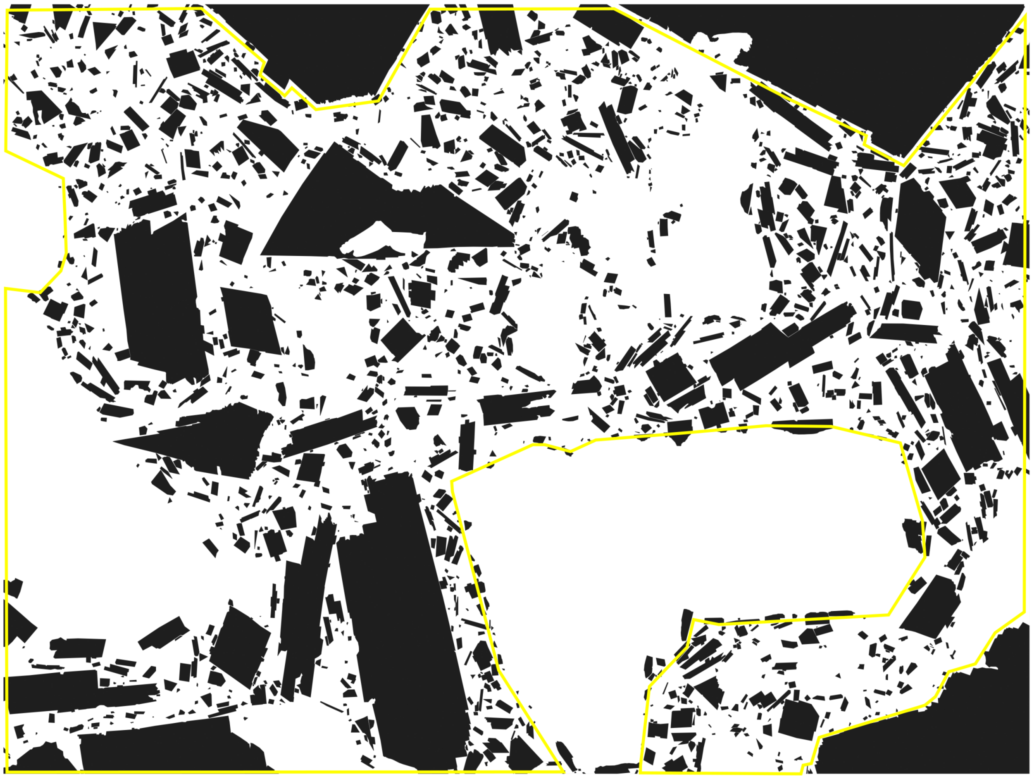

Sample: AB14

Image length: 0.957 mm

Analysed area: 0.505 mm^2

Number of feldspar crystals in analysed area (N): 1443

AB15 BSE image

Scoriaceous bomb

Explosion date: 17 January 2008

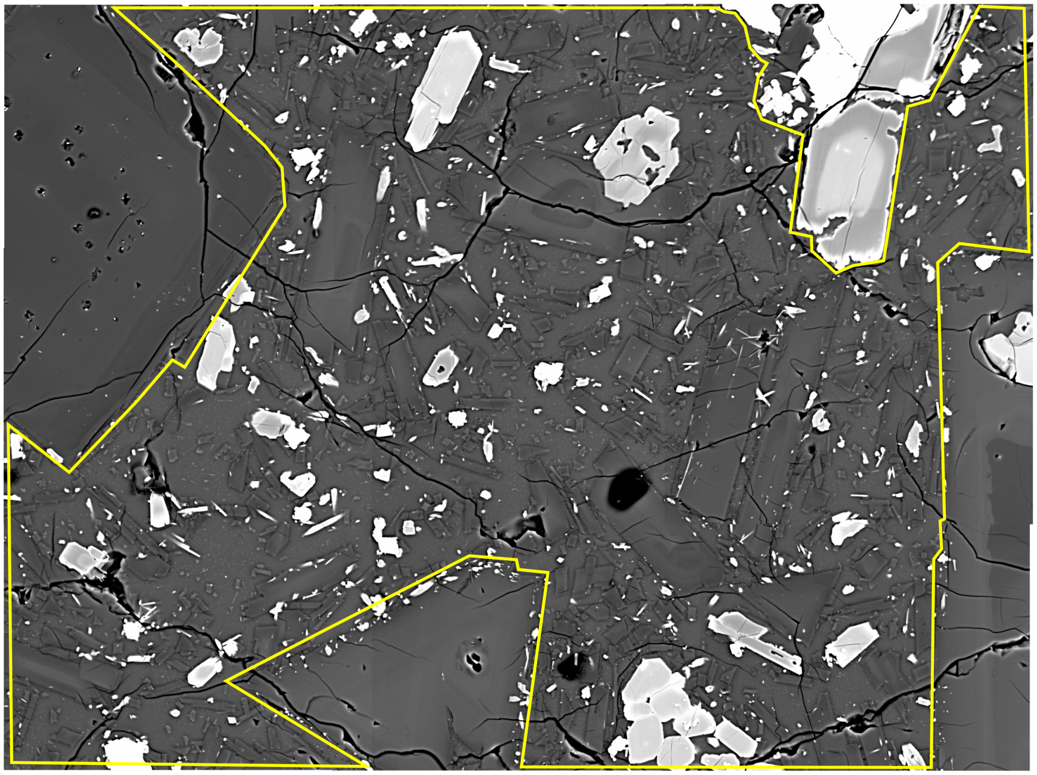

Sample: AB15

Image length: 0.577 mm

Analysed area: 0.178 mm^2

Number of feldspar crystals in analysed area (N): 828

AB15 Feldspar microlites
Scoriaceous bomb
Explosion date: 17 January 2008

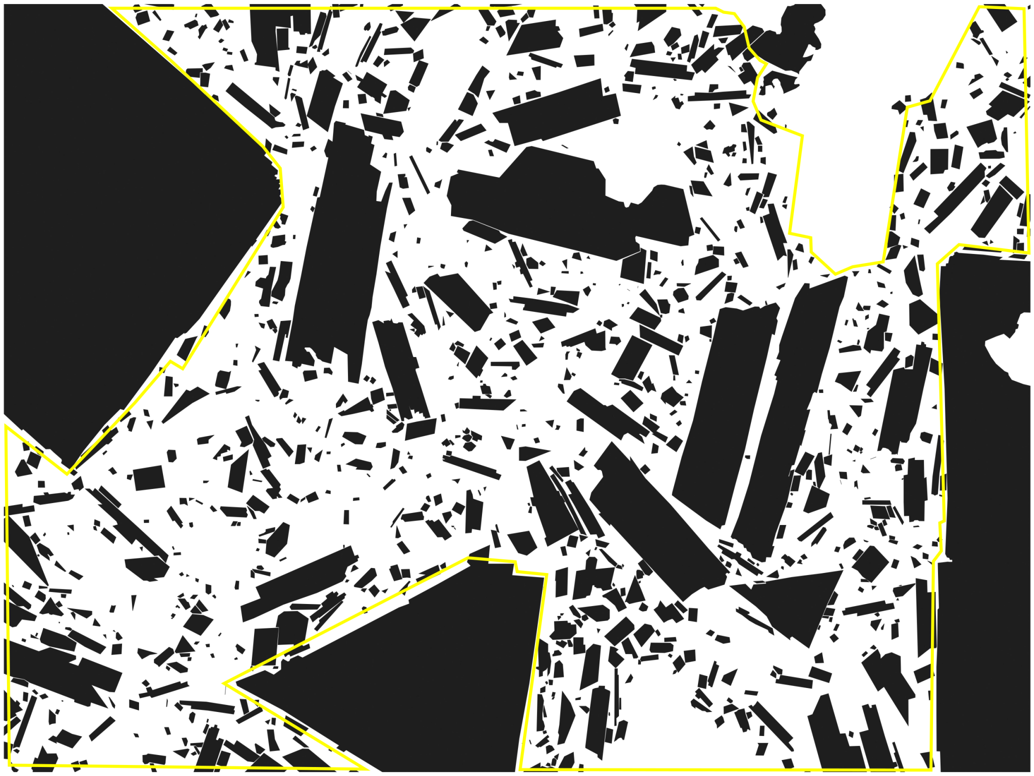

Sample: AB15
Image length: 0.577 mm
Analysed area: 0.178 mm^2
Number of feldspar crystals in analysed area (N): 828

AB16 BSE image

Inflated bomb rind

Explosion date: 20 February 2009

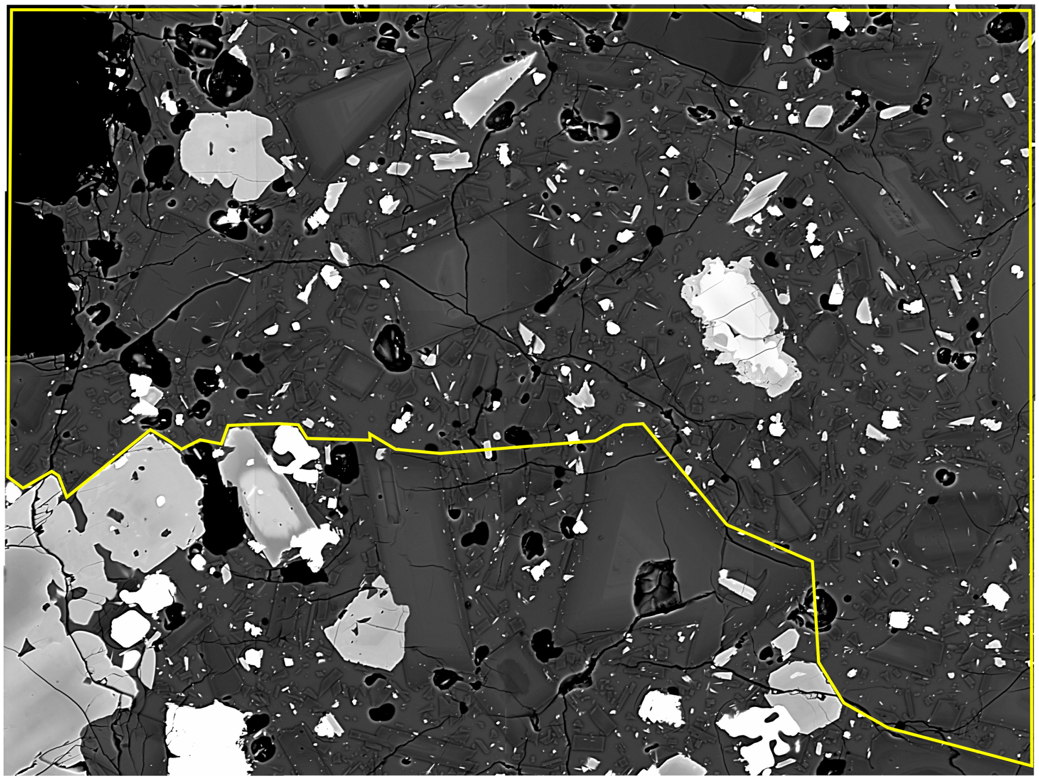

Sample: AB16

Image length: 0.758 mm

Analysed area: 0.29 mm^2

Number of feldspar crystals in analysed area (N): 1433

AB16 Feldspar microlites

Inflated bomb rind

Explosion date: 20 February 2009

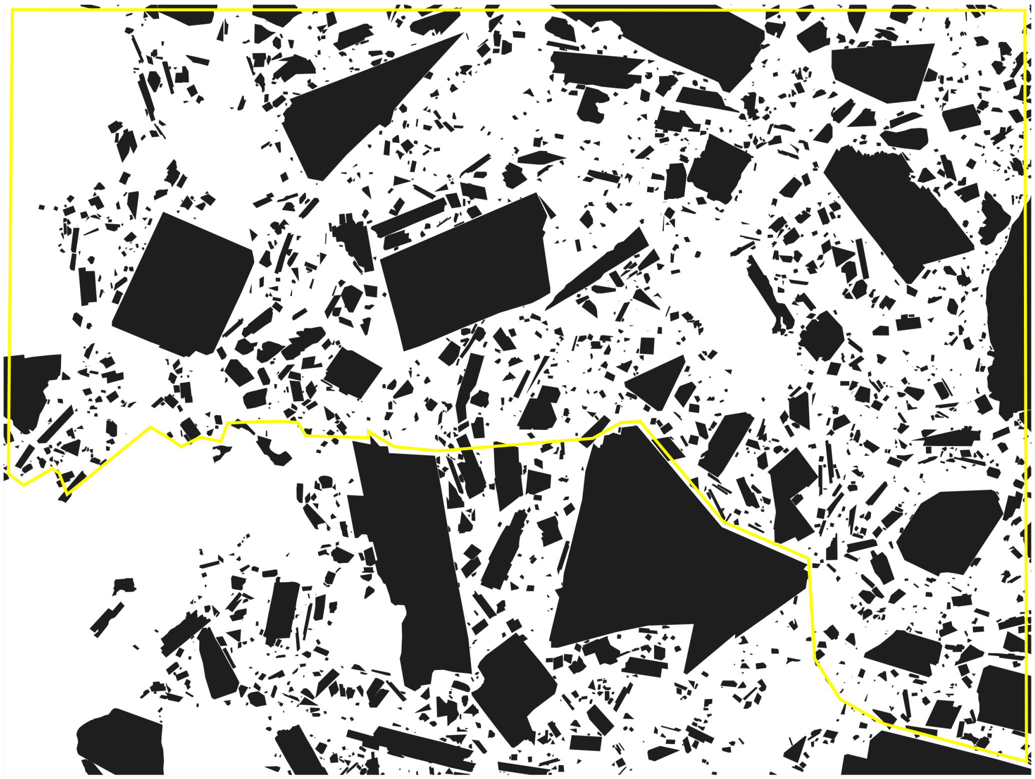

Sample: AB16

Image length: 0.758 mm

Analysed area: 0.29 mm^2

Number of feldspar crystals in analysed area (N): 1433

AB18 BSE image

Dense bomb

Explosion date: 20 February 2009

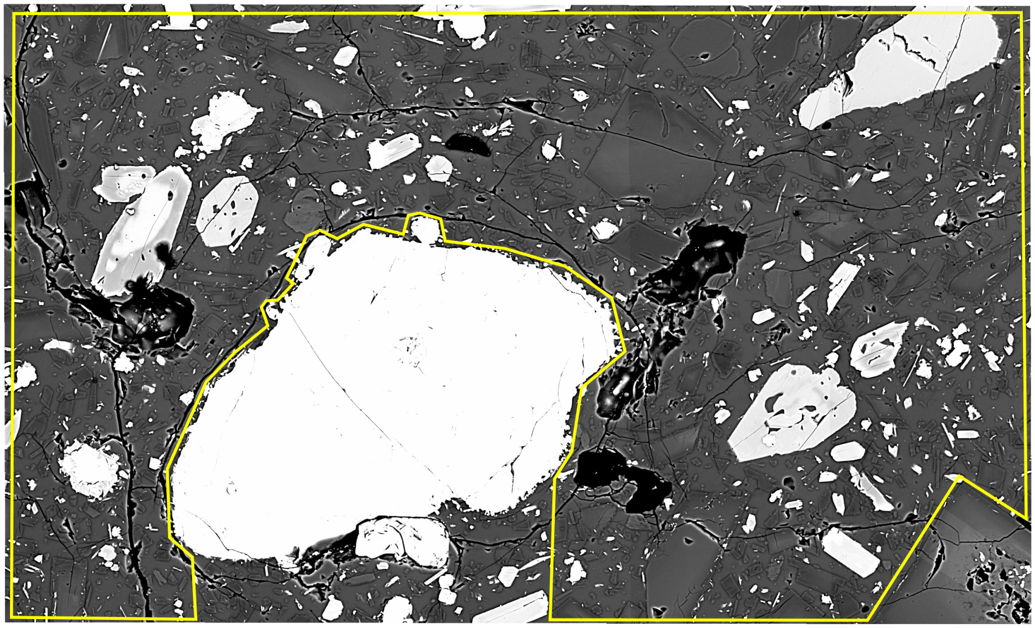

Sample: AB18

Image length: 0.93 mm

Analysed area: 0.379 mm^2

Number of feldspar crystals in analysed area (N): 1778

AB18 Feldspar microlites
Dense bomb
Explosion date: 20 February 2009

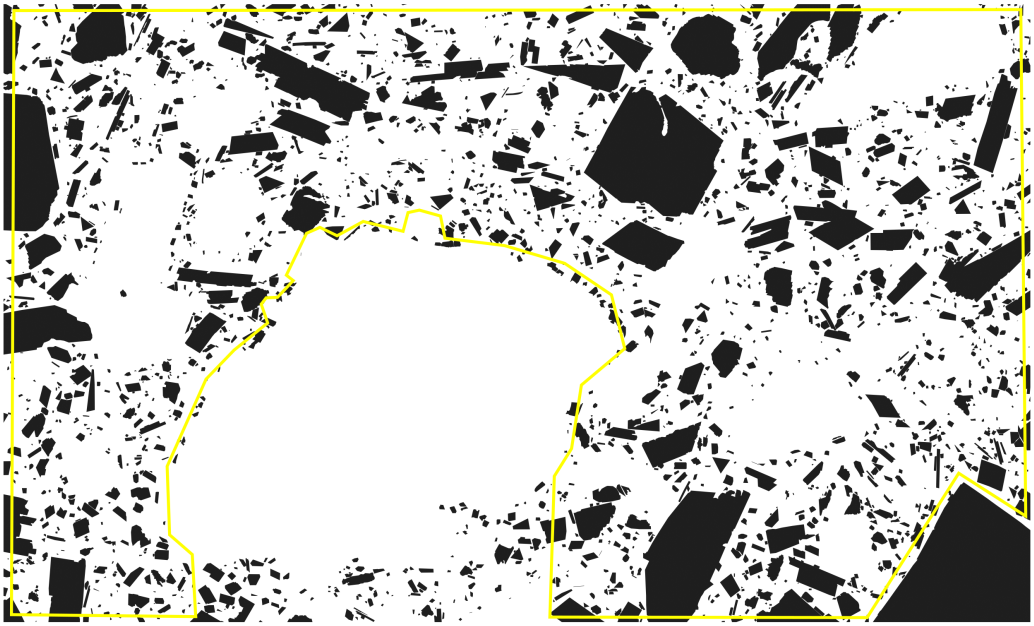

Sample: AB18
Image length: 0.93 mm
Analysed area: 0.379 mm^2
Number of feldspar crystals in analysed area (N): 1778

AB21 BSE image

Dense bomb

Explosion date: 2 January 2010

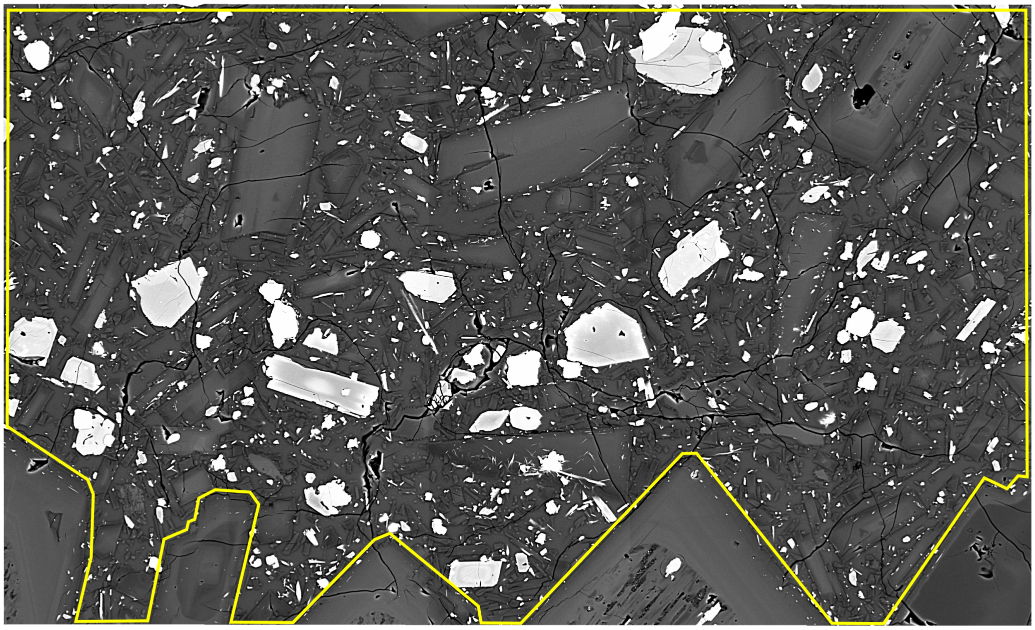

Sample: AB21

Image length: 0.948 mm

Analysed area: 0.477 mm^2

Number of feldspar crystals in analysed area (N): 2957

AB21 Feldspar microlites

Dense bomb

Explosion date: 2 January 2010

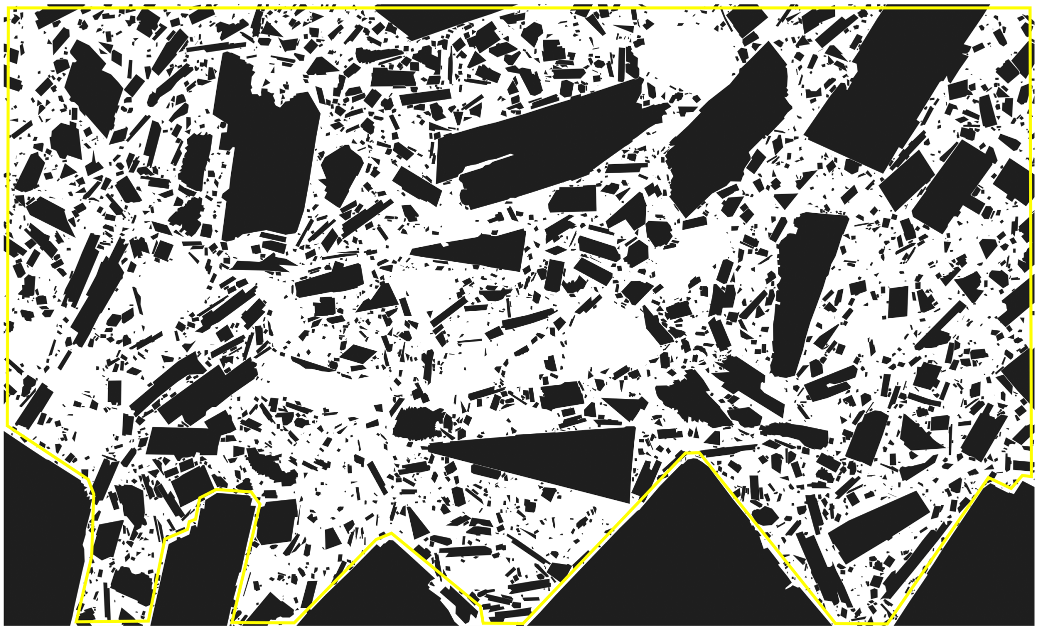

Sample: AB21

Image length: 0.948 mm

Analysed area: 0.477 mm^2

Number of feldspar crystals in analysed area (N): 2957

AB22 BSE image
Inflated bomb rind
Explosion date: 2 January 2010

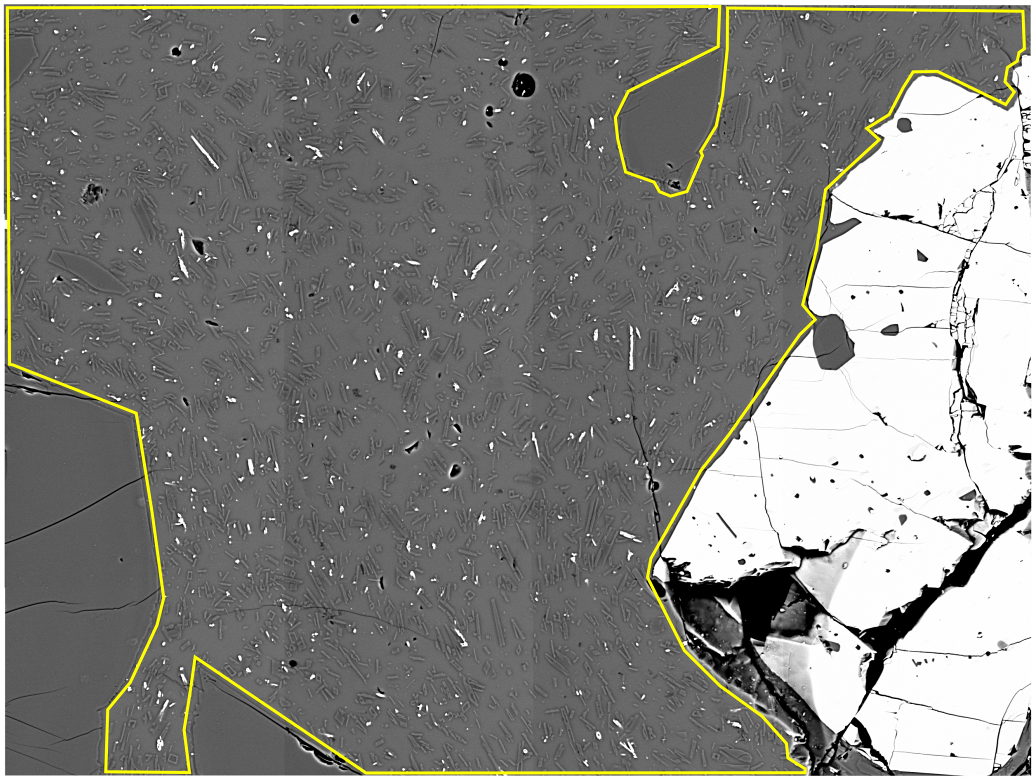

Sample: AB22
Image length (between arrows): 0.756 mm
Analysed area: 0.294 mm^2
Number of feldspar crystals in analysed area (N): 3737

AB22 Feldspar microlites

Inflated bomb rind

Explosion date: 2 January 2010

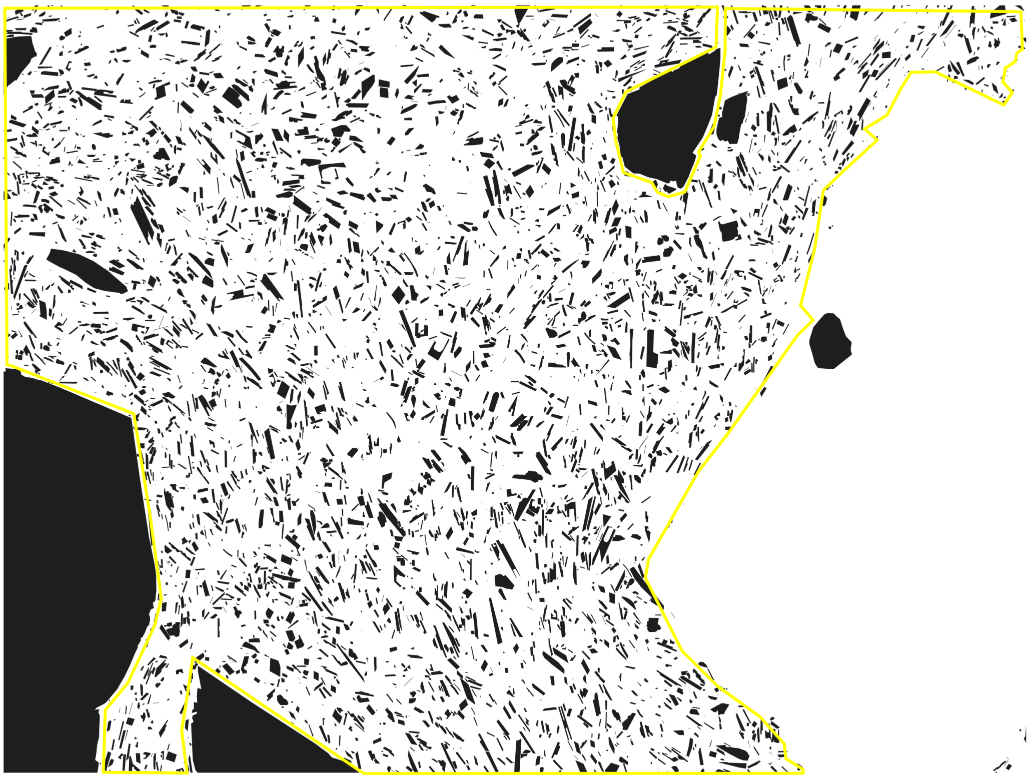

Sample: AB22

Image length (between arrows): 0.756 mm

Analysed area: 0.294 mm^2

Number of feldspar crystals in analysed area (N): 3737
