# Supplementary material for: Textural and geochemical constraints on andesitic plug emplacement prior to the 2004–2010 vulcanian explosions at Galeras volcano, Colombia
Source: Bull Volcanol. 2018 Dec 7;81(1):1. doi: 10.1007/s00445-018-1260-y (PMC6383983; doi:10.1007/s00445-018-1260-y)
Supplement: Supplementary file 4 — Photo-micrographs of bomb sample textures (PDF 182693 kb) [file 445_2018_1260_MOESM4_ESM.pdf]

Online Resource 4:
Sample photomicrographs

Textural and geochemical constraints on andesitic plug emplacement prior to the 2004-2010 vulcanian explosions at Galeras volcano, Colombia

Bulletin of Volcanology

Amelia A. Bain, Eliza S. Calder, Joaquín A. Cortés, Gloria Patricia Cortés J., Susan C. Loughlin

This online resource provides plane polarised light images of each bomb sample, along with the areal % of vesicles, phenocrysts and groundmass estimated from thin sections. As there is typically no crystal alignment in these samples, the areal % is considered equivalent to the volume %.

Dense and scoriaceous bombs are listed in the order of increasing vesicularity so that the gradual transition in vesicularity between these bomb types is shown. Differences in the phenocryst content are mainly due to differences in vesicularity and minor variation in phenocryst content.

Examples of features of interest are labelled in yellow:

Pl = plagioclase phenocryst
Px = pyroxene phenocryst
Fe-Ti = Fe-Ti phenocryst
A = Amphibole
Gr = groundmass
V = vesicle

Dense bombs

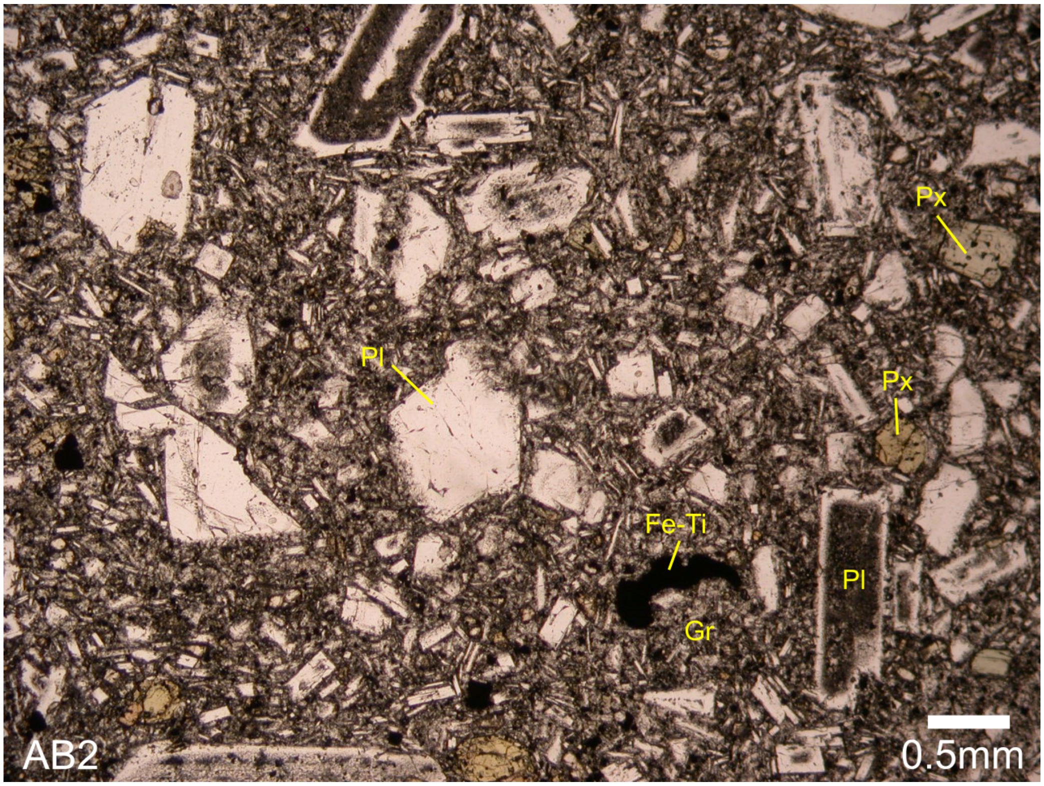

AB2
Vesicles: **0%**
Phenocrysts: 40%
Plagioclase 25%
Pyroxenes 10%
Fe-Ti Oxides 5%
Groundmass: 60%

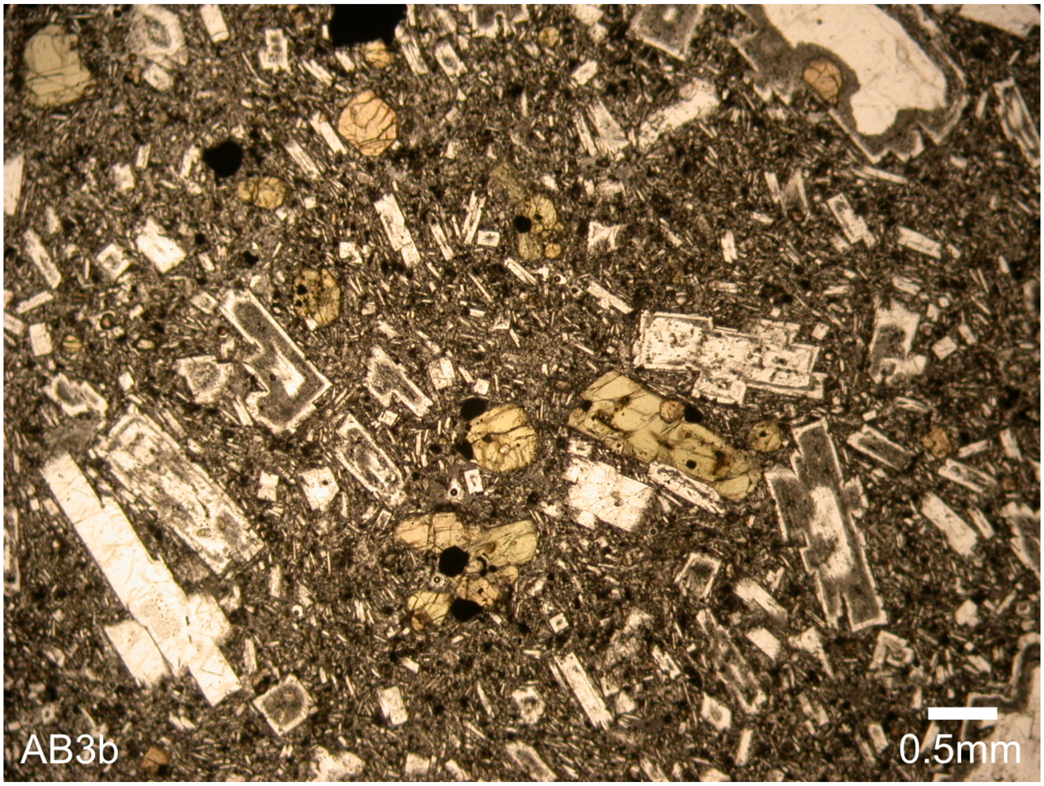

AB3b
Vesicles: **0%**
Phenocrysts: 35%
Plagioclase 20%
Pyroxenes 10%
Fe-Ti Oxides 5%
Groundmass: 65%

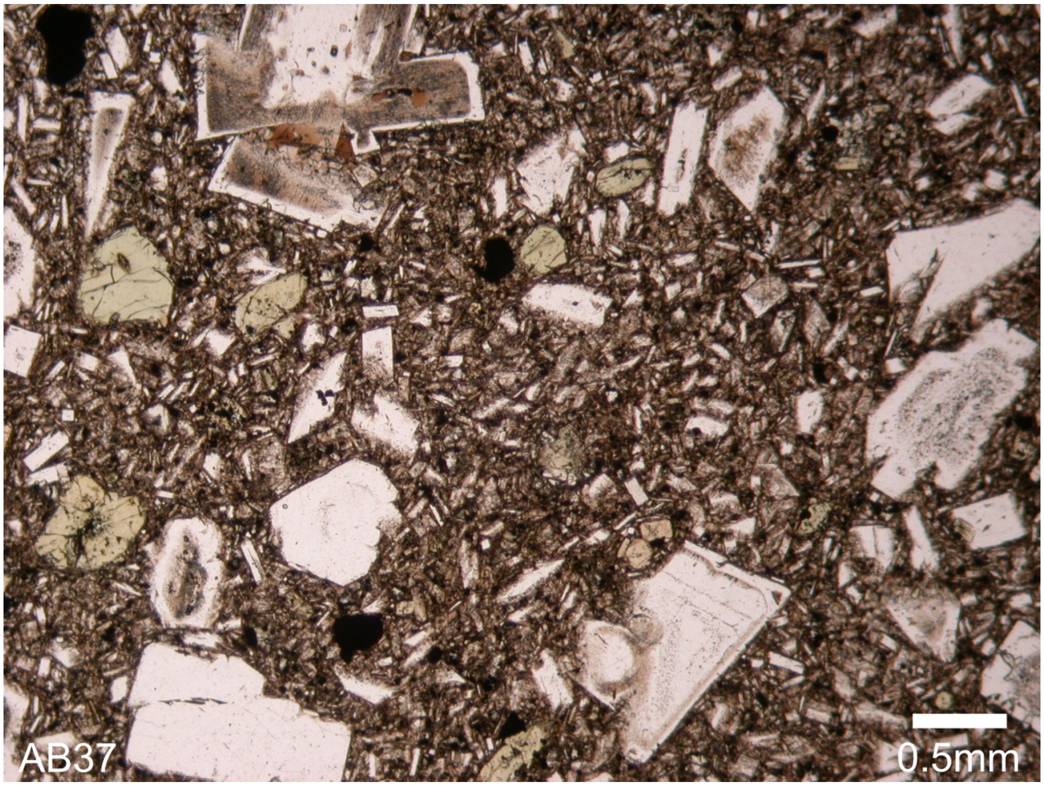

AB37
Vesicles: **0%**
Phenocrysts: 30%
Plagioclase 20%
Pyroxenes 7%
Fe-Ti Oxides 3%
Groundmass: 70%

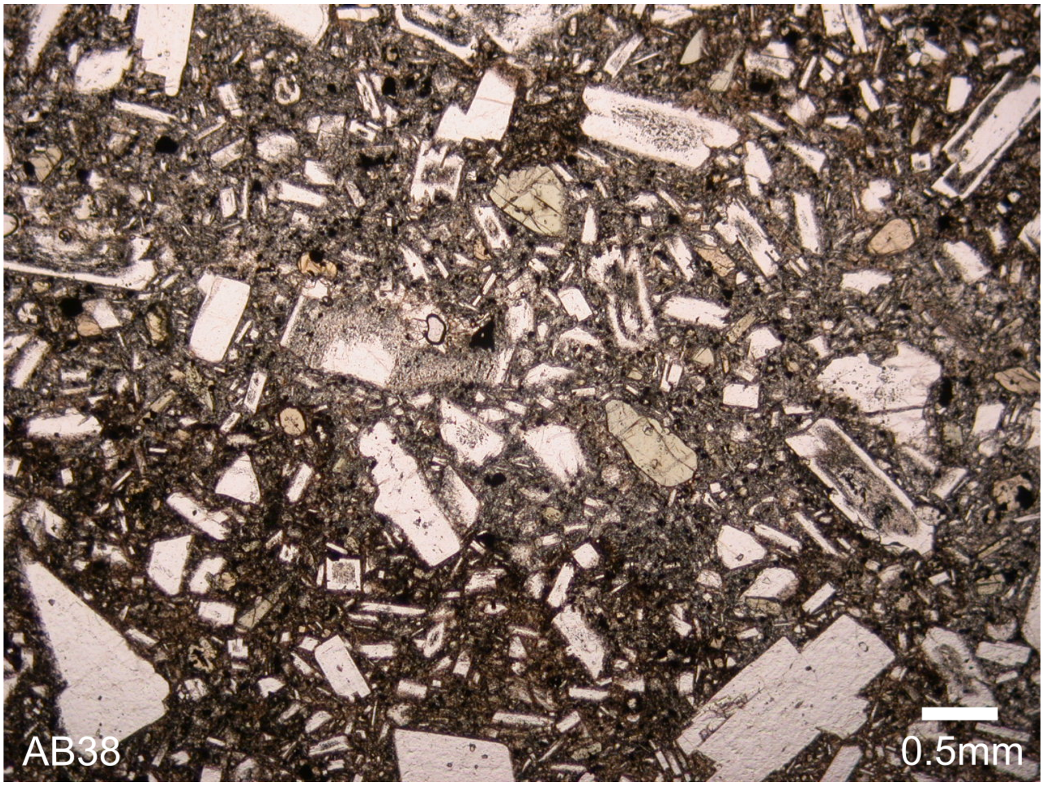

AB38
Vesicles: **0%**
Phenocrysts: 33%
Plagioclase 20%
Pyroxenes 10%
Fe-Ti Oxides 3%
Groundmass: 67%

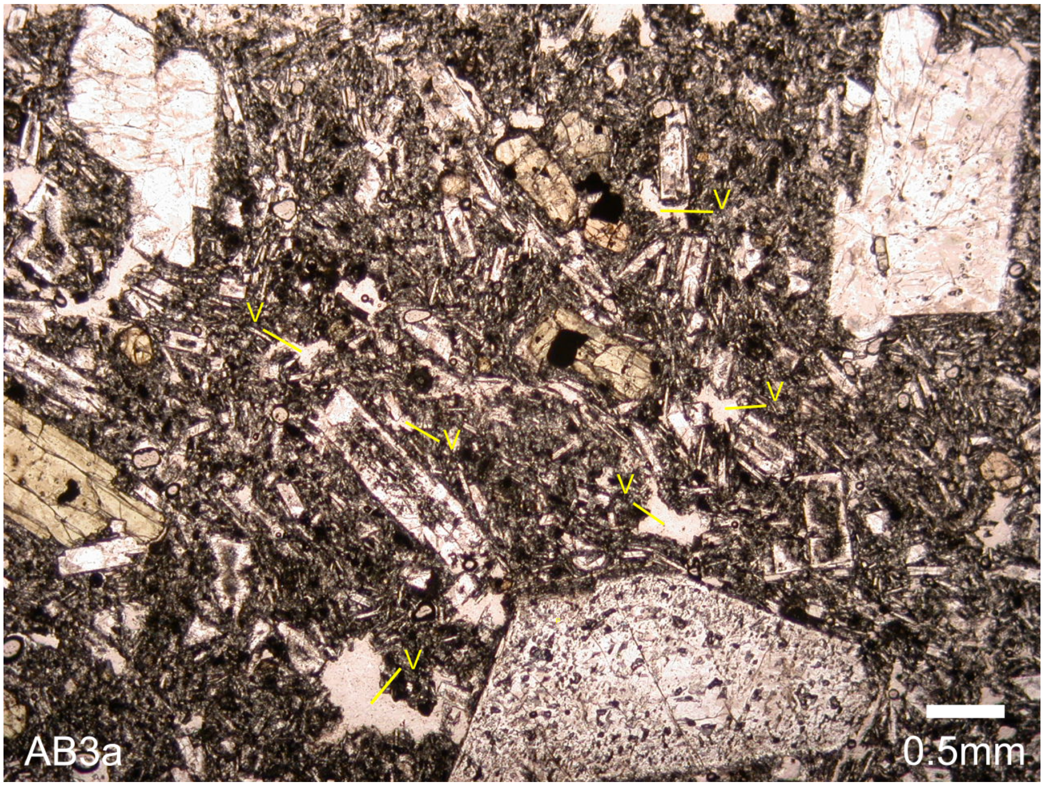

AB3a
Vesicles: **0.5%**
Phenocrysts: 26%
Plagioclase 20%
Pyroxenes 5%
Fe-Ti Oxides 5%
Groundmass: 73.5%

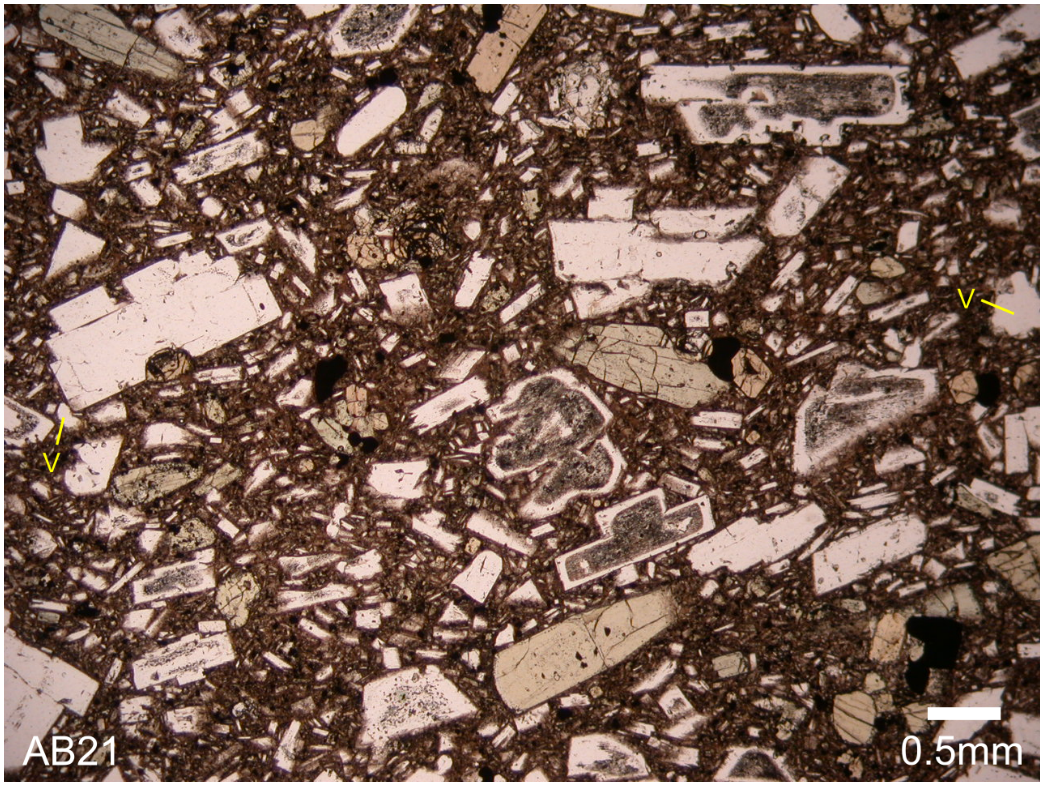

AB21
Vesicles: **0.5%**
Phenocrysts: 32%
Plagioclase 20%
Pyroxenes 10%
Fe-Ti Oxides 2%
Groundmass: 67.5%

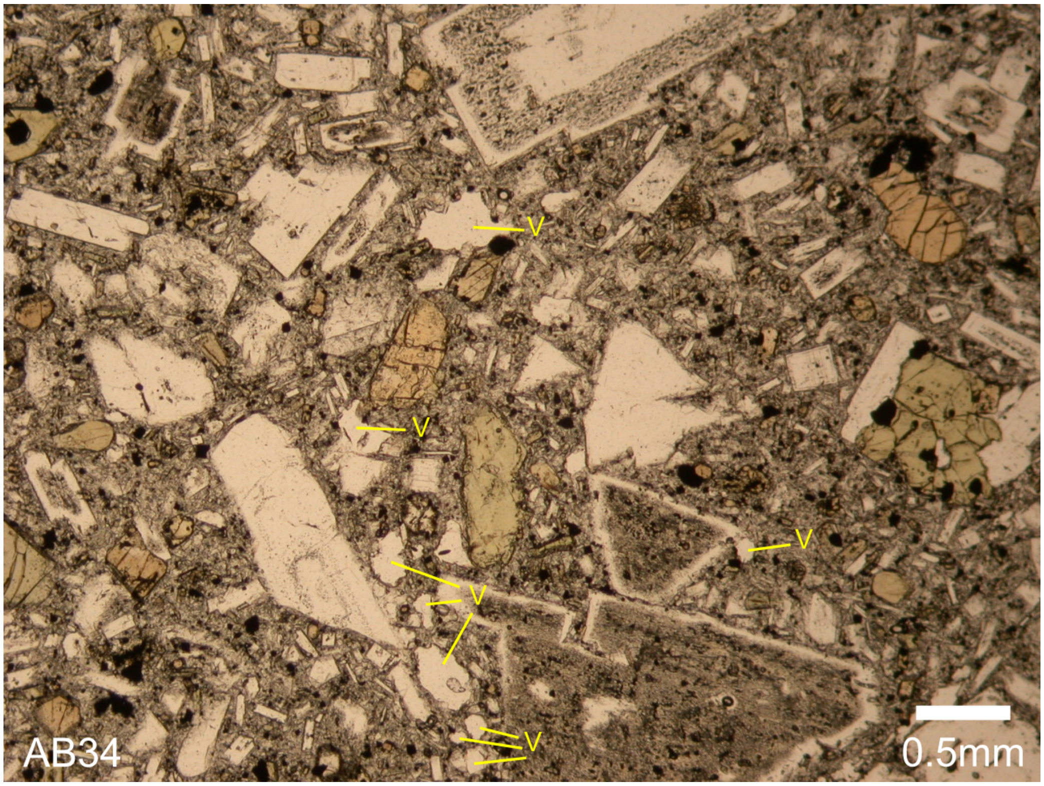

AB34
Vesicles: **0.5%**
Phenocrysts: 31%
Plagioclase 20%
Pyroxenes 8%
Fe-Ti Oxides 3%
Groundmass: 68.5%

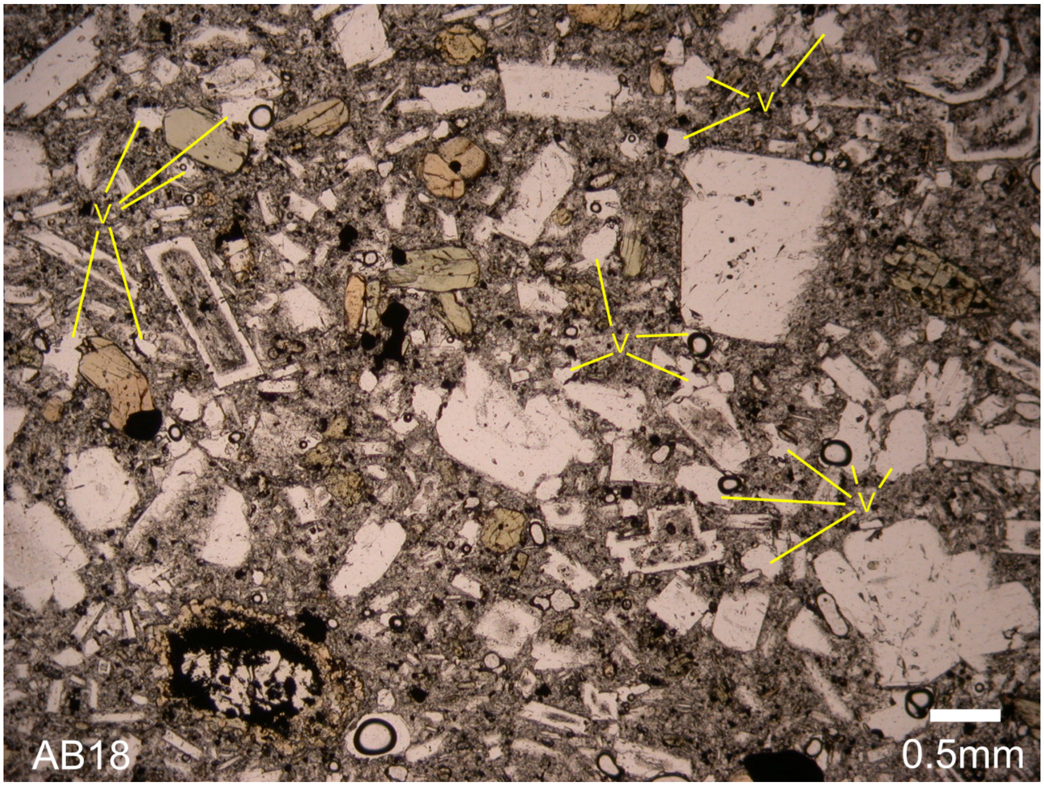

AB18
Vesicles: **5%**
Phenocrysts: 28%
Plagioclase 15%
Pyroxenes 10%
Fe-Ti Oxides 3%
Groundmass: 67%

Scoriaceous bombs

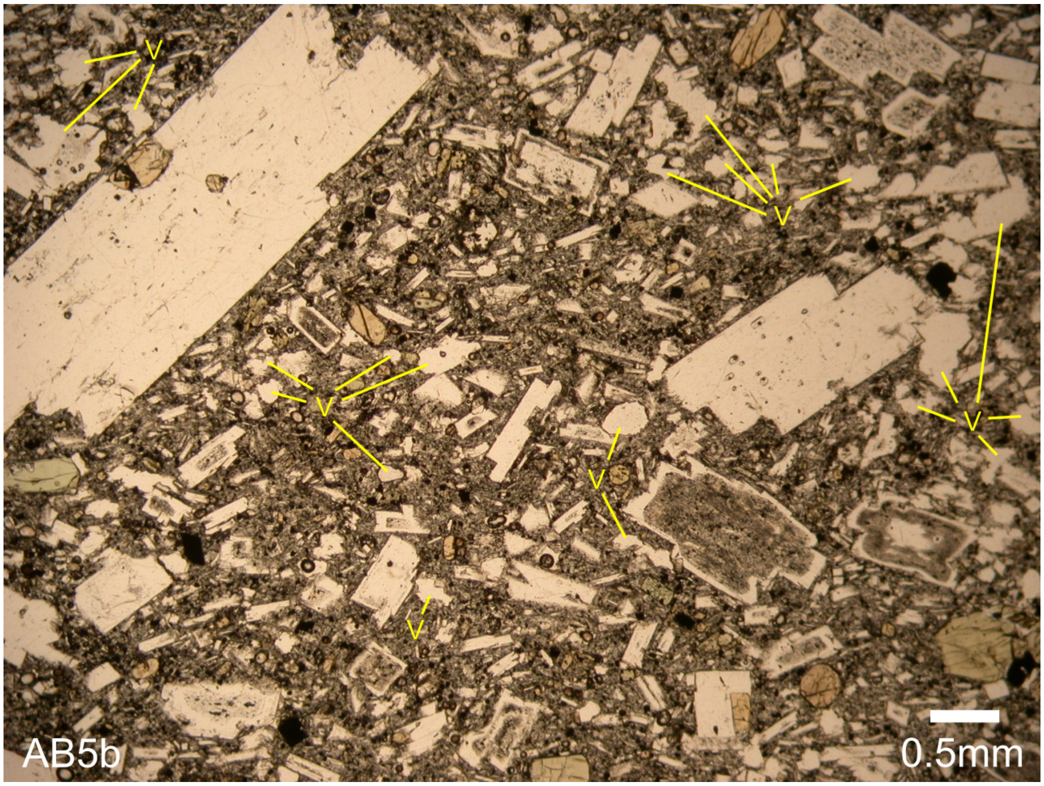

AB5b
Vesicles: **3%**
Phenocrysts: 30%
Plagioclase 20%
Pyroxenes 7%
Fe-Ti Oxides 3%
Groundmass: 67%

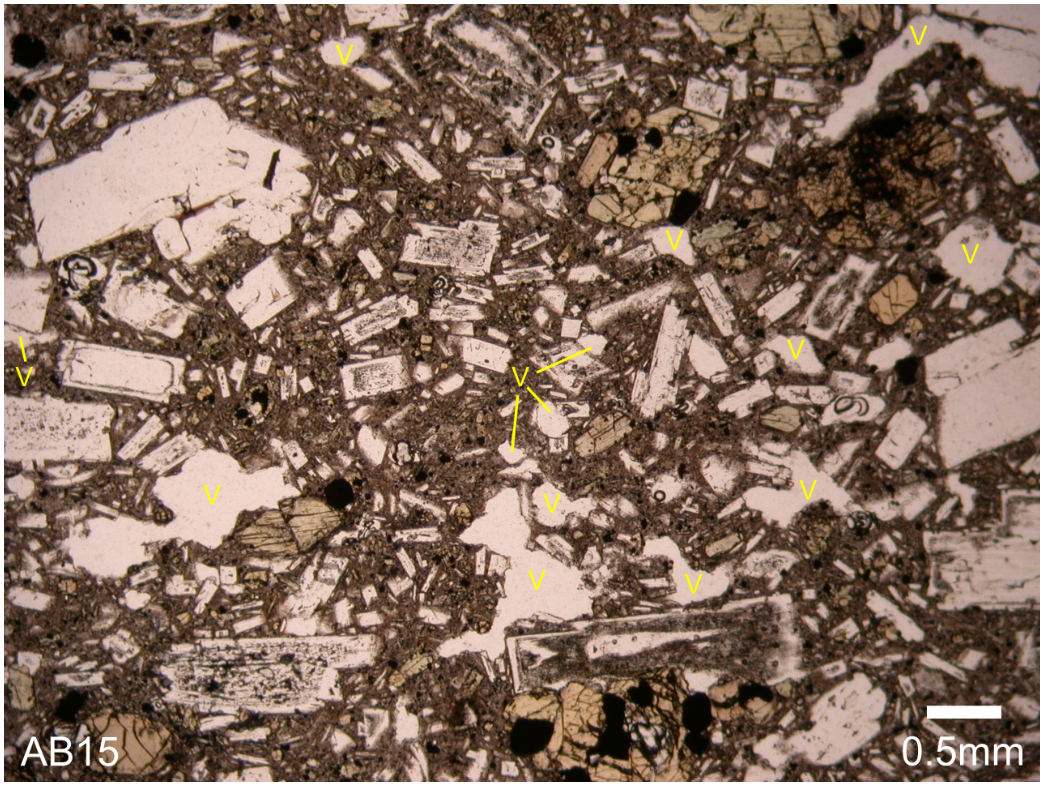

AB15
Vesicles: **5%**
Phenocrysts: 30%
Plagioclase 20%
Pyroxenes 7%
Fe-Ti Oxides 3%
Groundmass: 65%

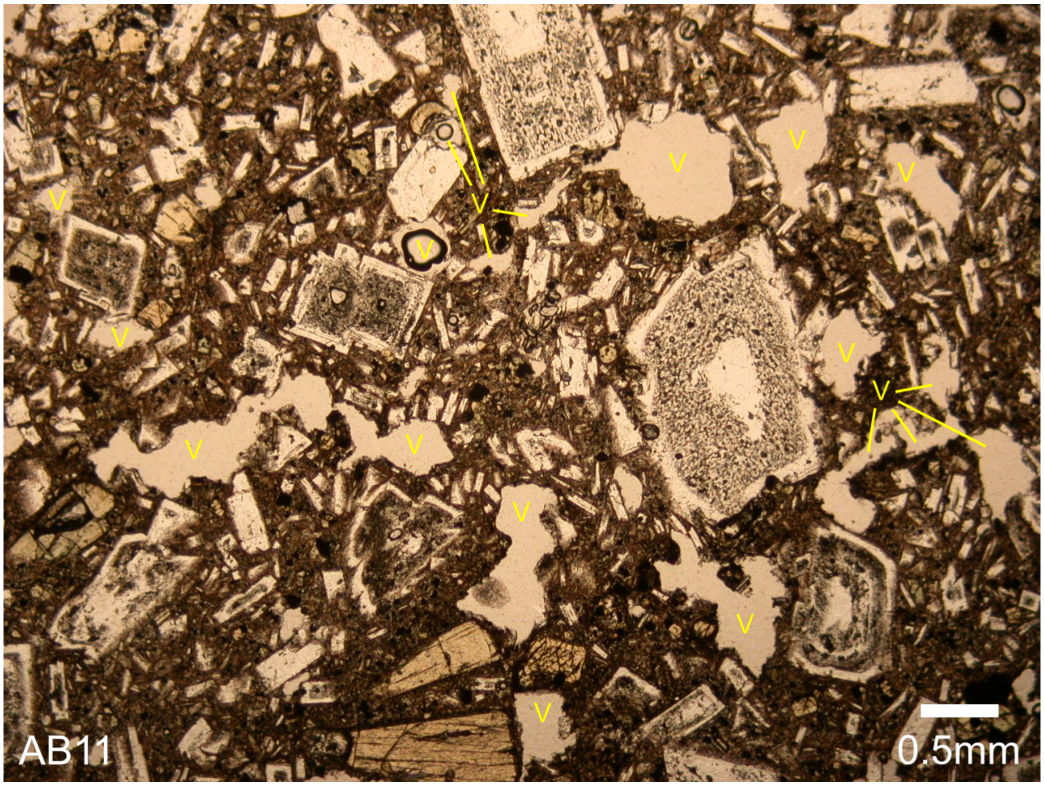

AB11
Vesicles: **7%**
Phenocrysts: 30%
Plagioclase 20%
Pyroxenes 7%
Fe-Ti Oxides 3%
Groundmass: 63%

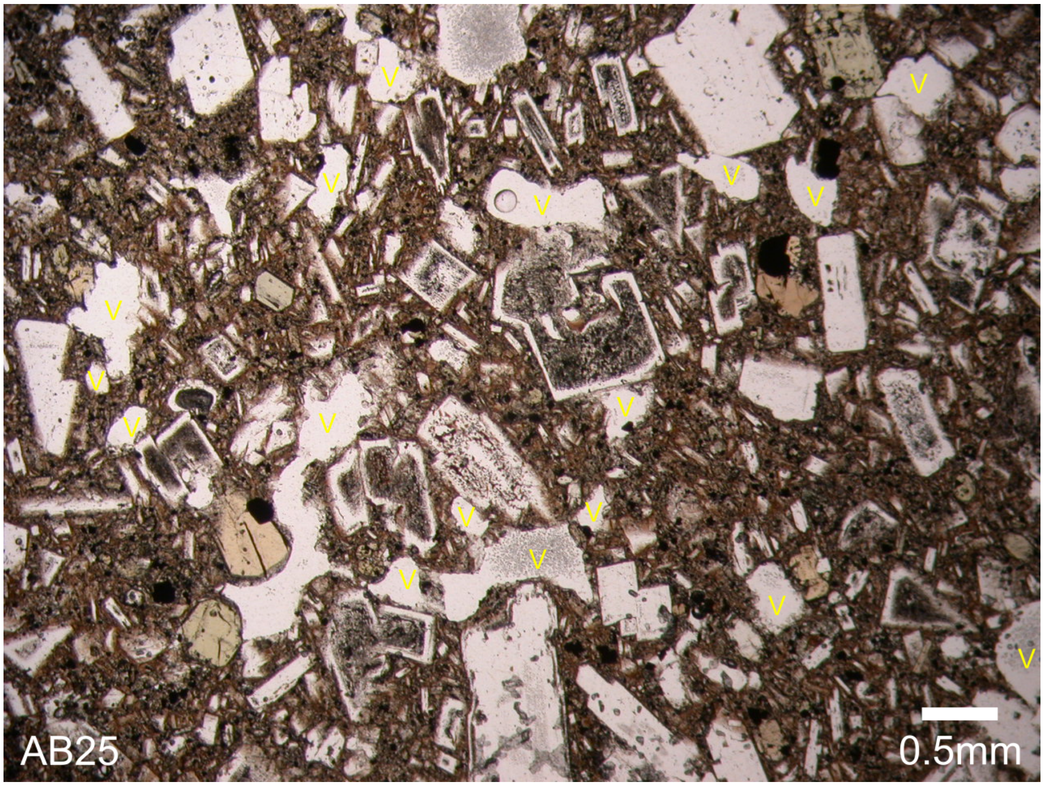

AB25
Vesicles: **7%**
Phenocrysts: 22%
Plagioclase 15%
Pyroxenes 5%
Fe-Ti Oxides 2%
Groundmass: 71%

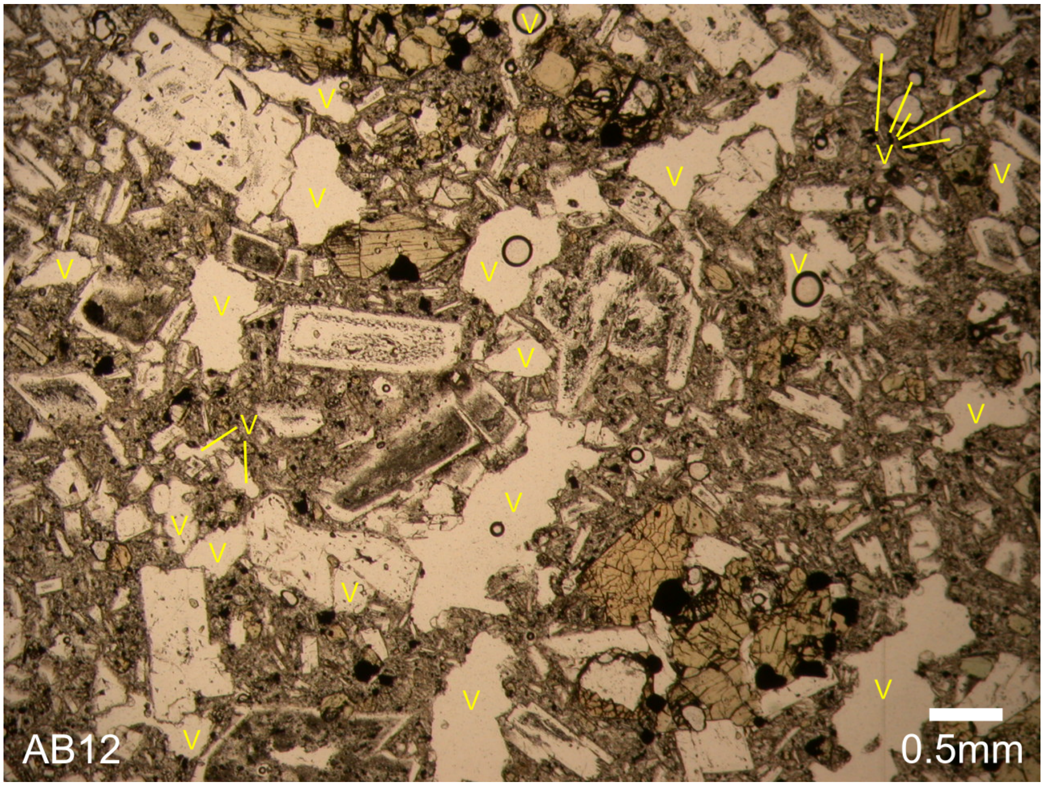

AB12
Vesicles: **10%**
Phenocrysts: 30%
Plagioclase 20%
Pyroxenes 7%
Fe-Ti Oxides 3%
Groundmass: 60%

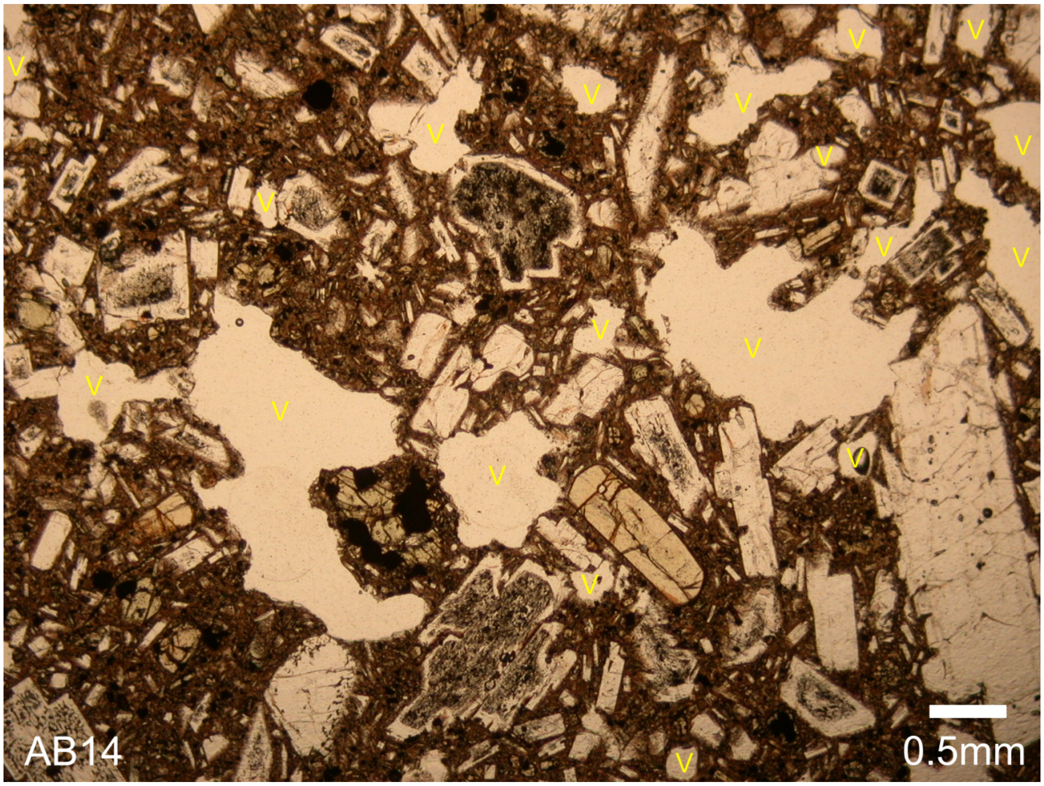

AB14
Vesicles: **10%**
Phenocrysts: 28%
Plagioclase 15%
Pyroxenes 10%
Fe-Ti Oxides 3%
Groundmass: 62%

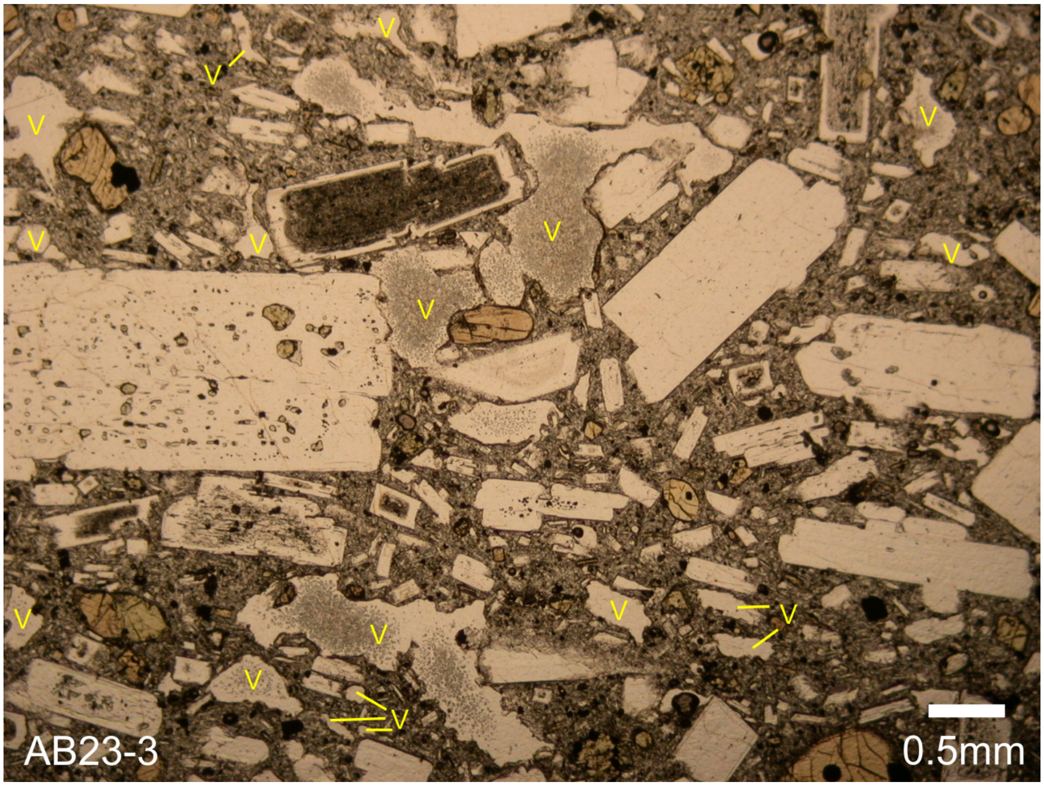

AB23-3
Vesicles: **12%**
Phenocrysts: 2/%
Plagioclase 15%
Pyroxenes 10%
Fe-Ti Oxides 3%
Groundmass: 62%

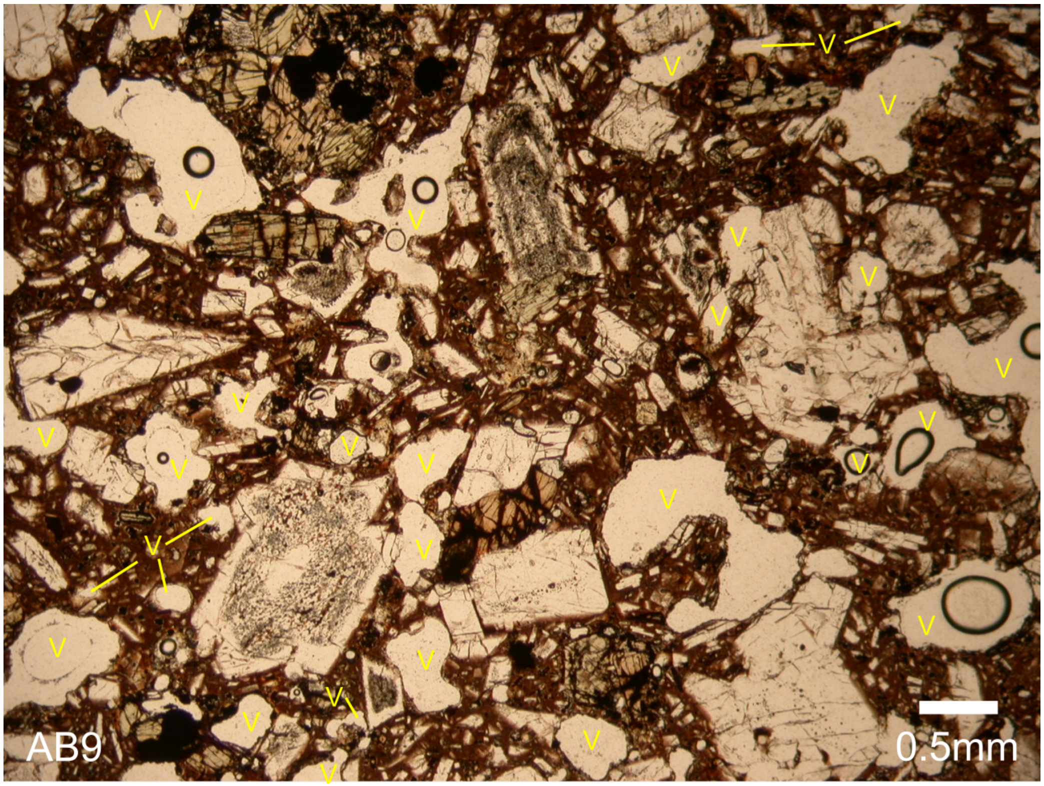

AB9
Vesicles: **15%**
Phenocrysts: 30%
Plagioclase 20%
Pyroxenes 7%
Fe-Ti Oxides 3%
Groundmass: 33%

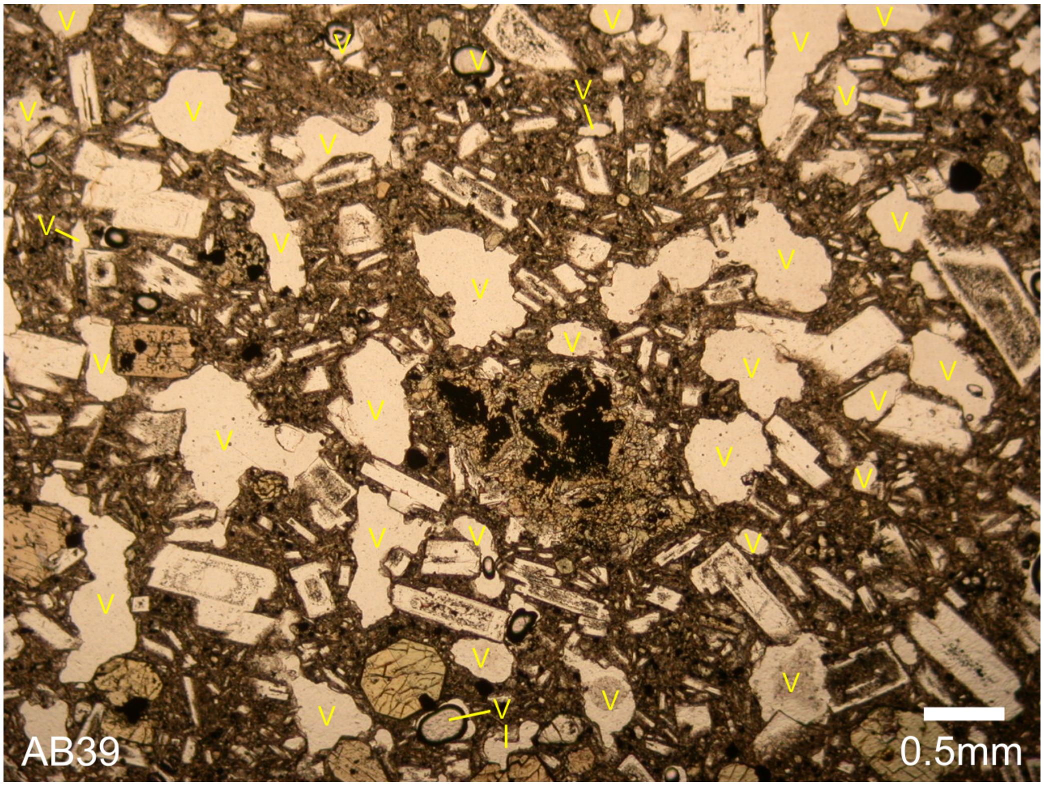

AB39
Vesicles: **15%**
Phenocrysts: 23%
Plagioclase 15%
Pyroxenes 5%
Fe-Ti Oxides 3%
Groundmass: 62%

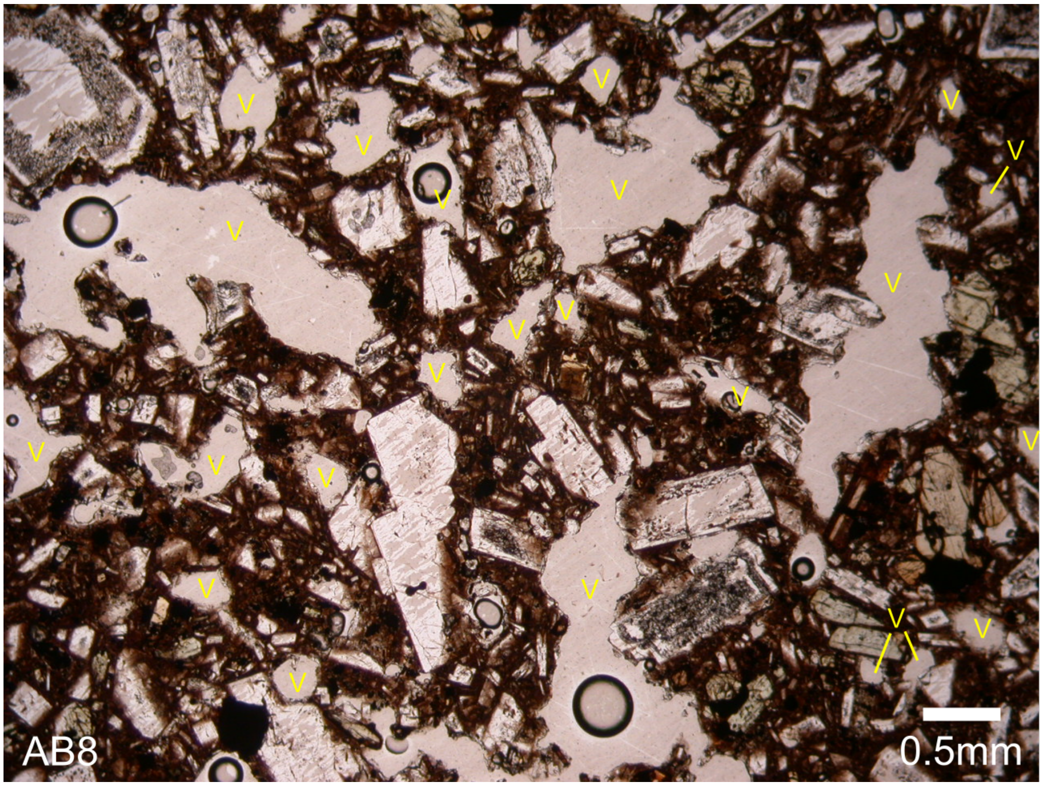

AB8
Vesicles: **20%**
Phenocrysts: 25%
Plagioclase 15%
Pyroxenes 7%
Fe-Ti Oxides 3%
Groundmass: 55%

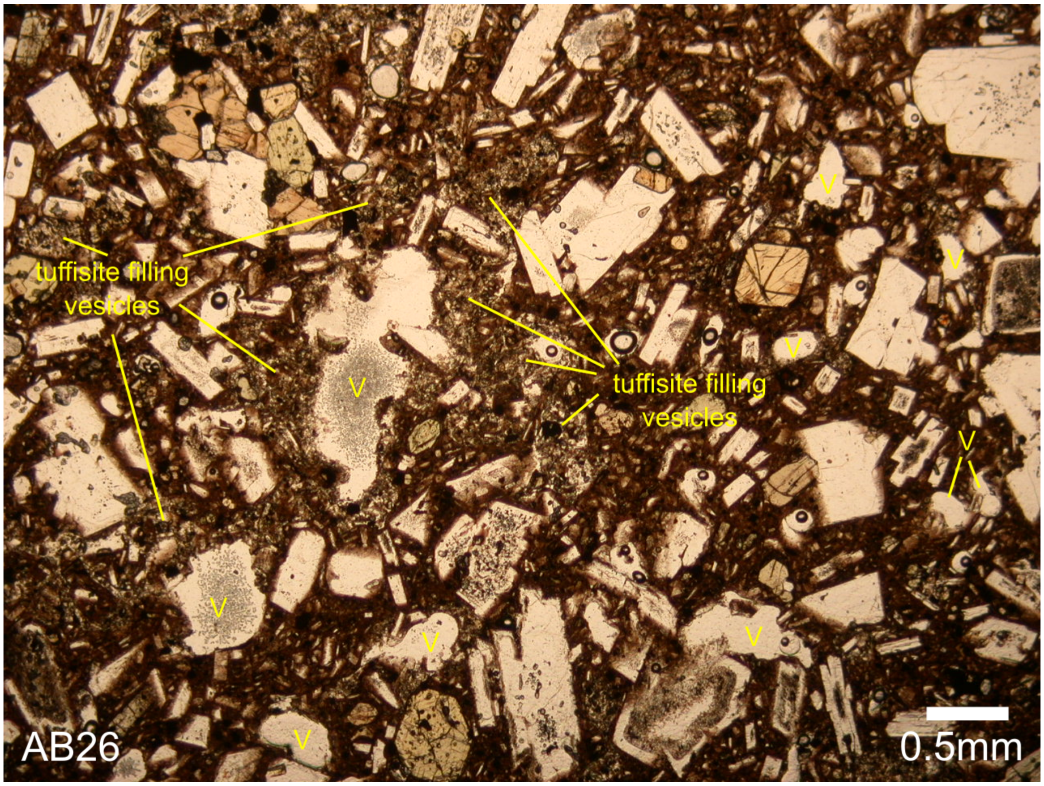

AB26
Vesicles: **20%** (n.b.: approx. 50 % of vesicles are filled with partially-annealed ash fragments from tuffisite vein creation event)
Phenocrysts: 30%
Plagioclase 15%
Pyroxenes 10%
Fe-Ti Oxides 5%
Groundmass: 50%

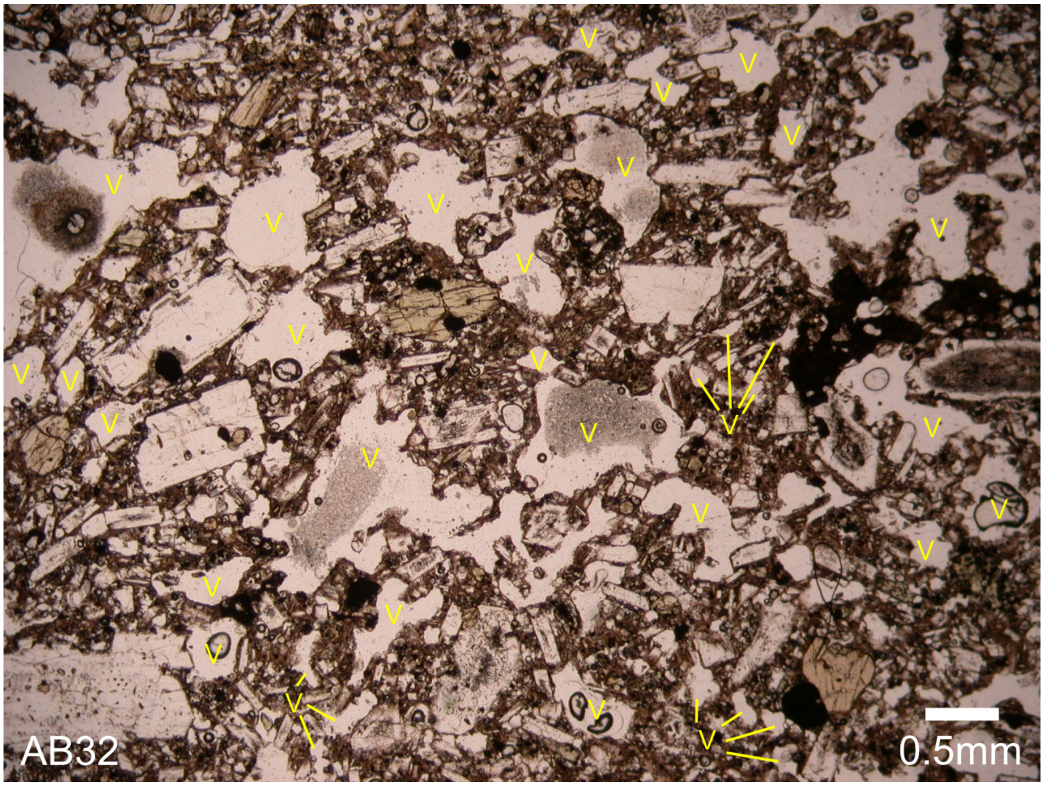

AB32
Vesicles: **25%**
Phenocrysts: 24%
Plagioclase 15%
Pyroxenes 7%
Fe-Ti Oxides 2%
Groundmass: 51%

Inflated bombs

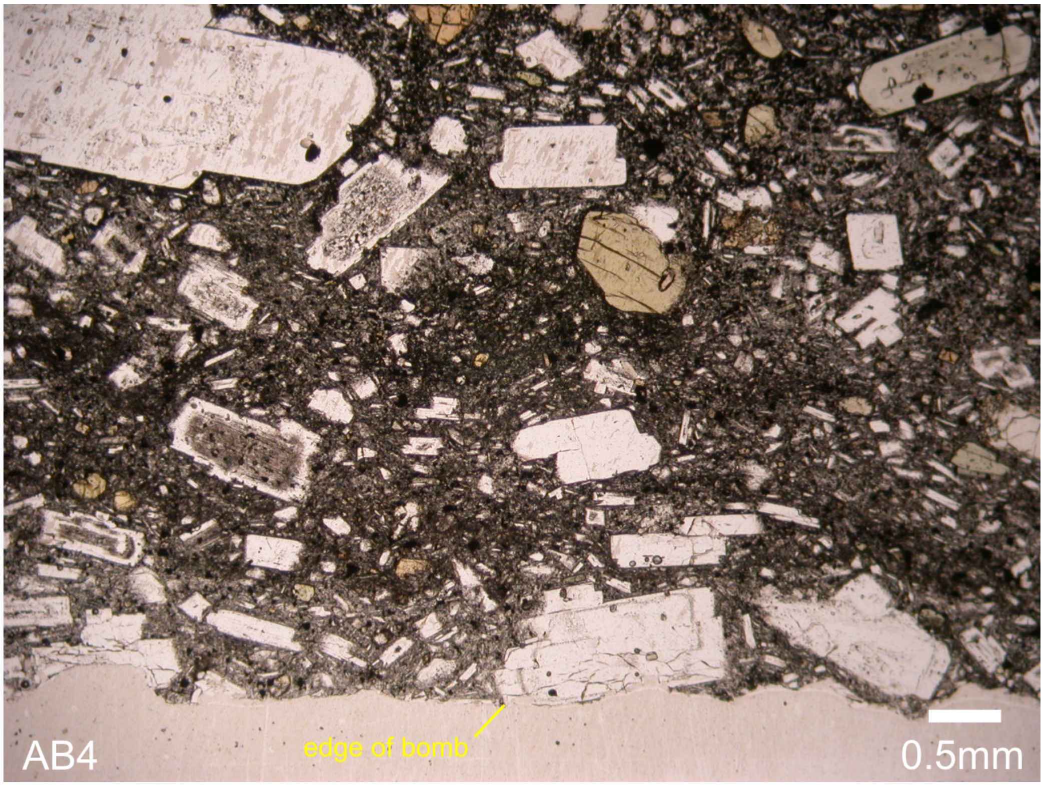

AB4 Rind
Vesicles: **0%**
Phenocrysts: 30%
Plagioclase 20%
Pyroxenes 7%
Fe-Ti Oxides 3%
Groundmass: 70%

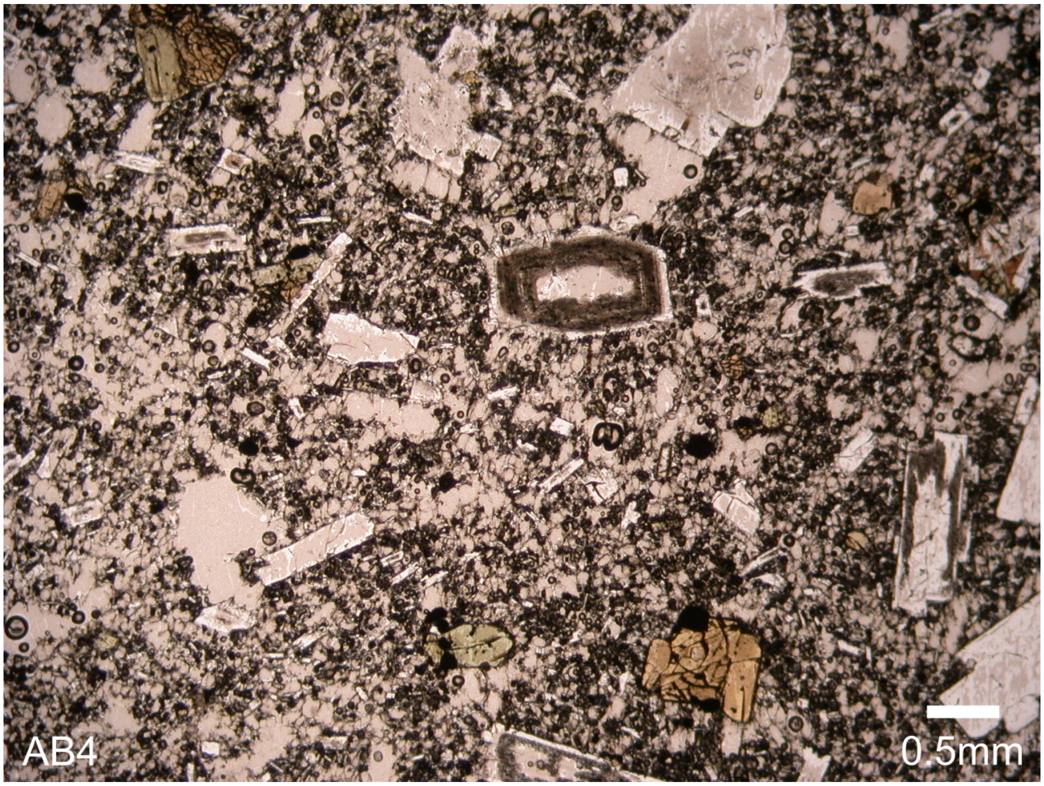

AB4 Interior
Vesicles: **70%**
Phenocrysts: 14%
Plagioclase 10%
Pyroxenes 3%
Fe-Ti Oxides 1%
Groundmass: 16%

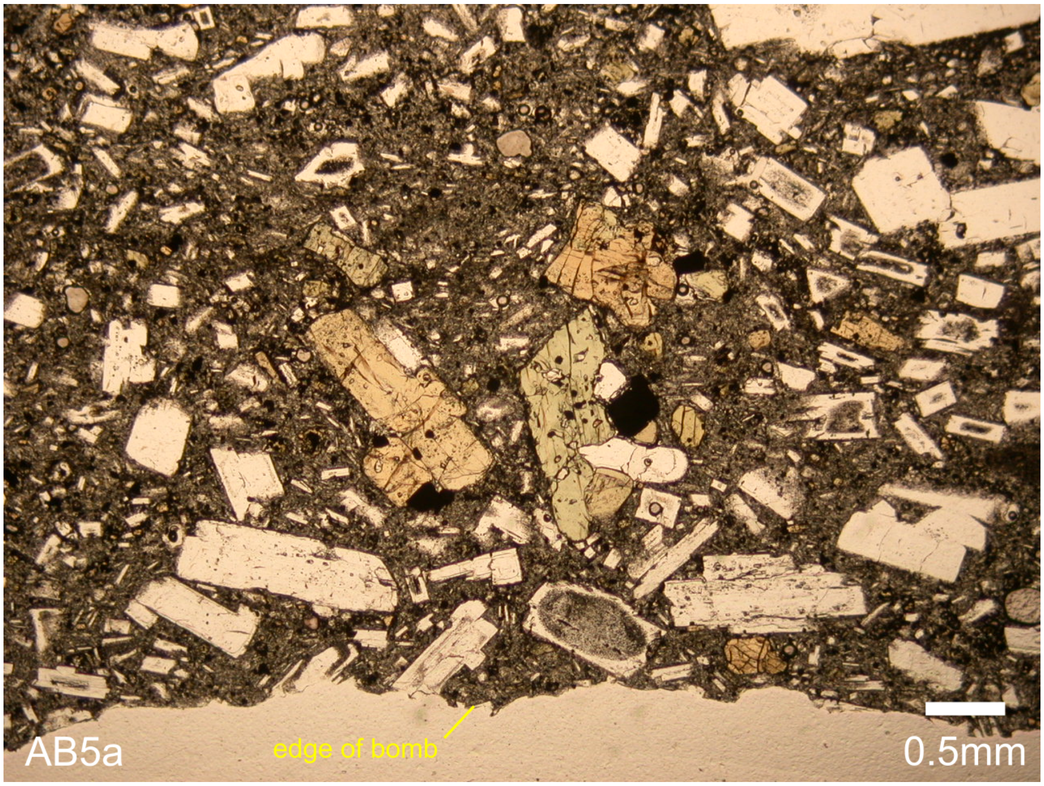

AB5a rind
Vesicles: **0.5%**
Phenocrysts: 29%
Plagioclase 20%
Pyroxenes 7%
Fe-Ti Oxides 2%
Groundmass: 70.5%

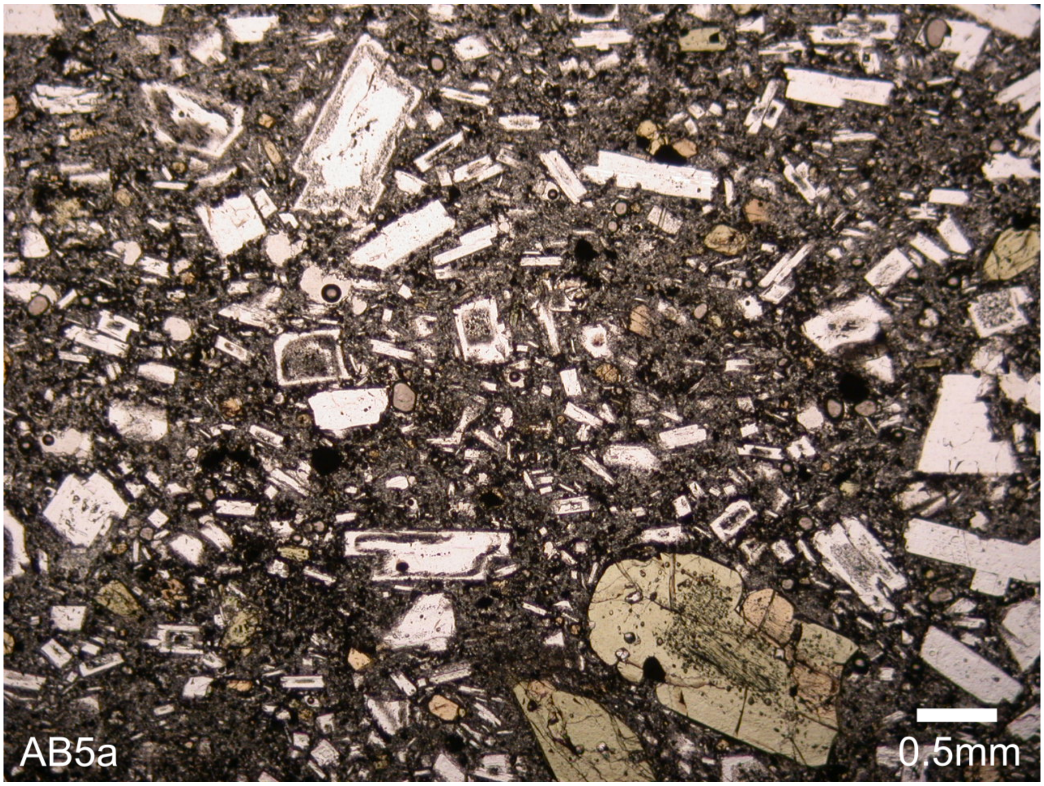

AB5a interior
Vesicles: **10%**
Phenocrysts: 32%
Plagioclase 20%
Pyroxenes 10%
Fe-Ti Oxides 2%
Groundmass: 58%

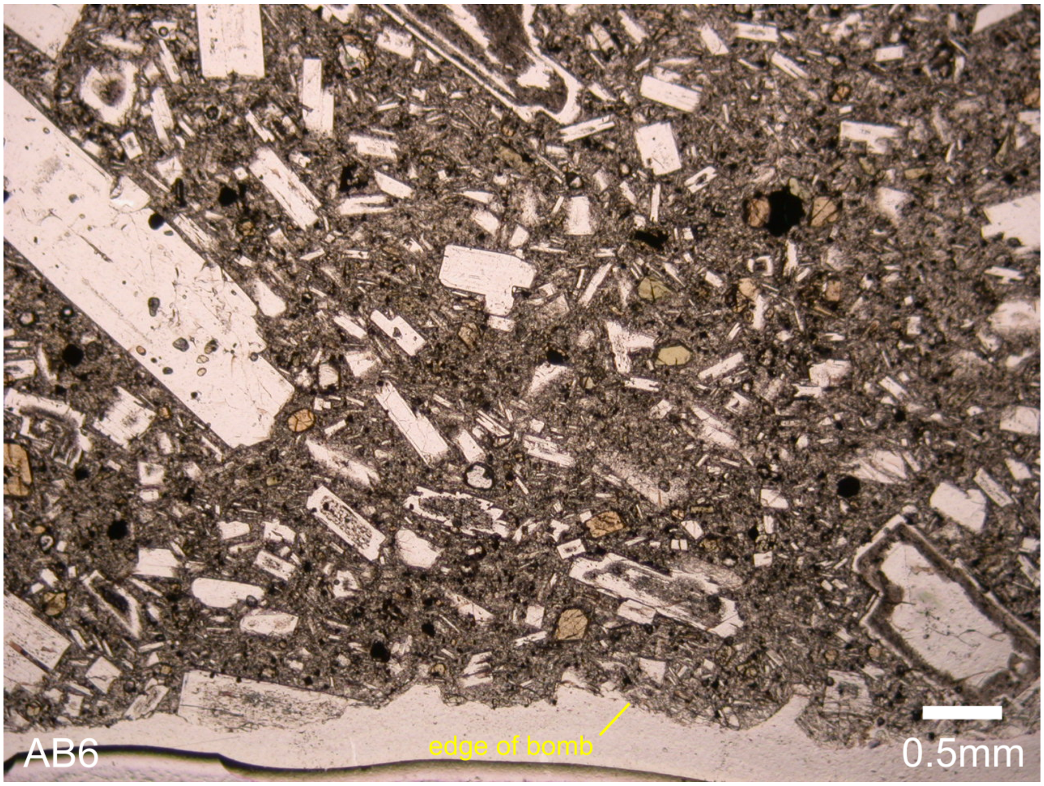

AB6 Rind
<u>Vesicles:</u> **0.5%**
<u>Phenocrysts:</u> 30%
Plagioclase 20%
Pyroxenes 7%
Fe-Ti Oxides 3%
<u>Groundmass:</u> 69.5%

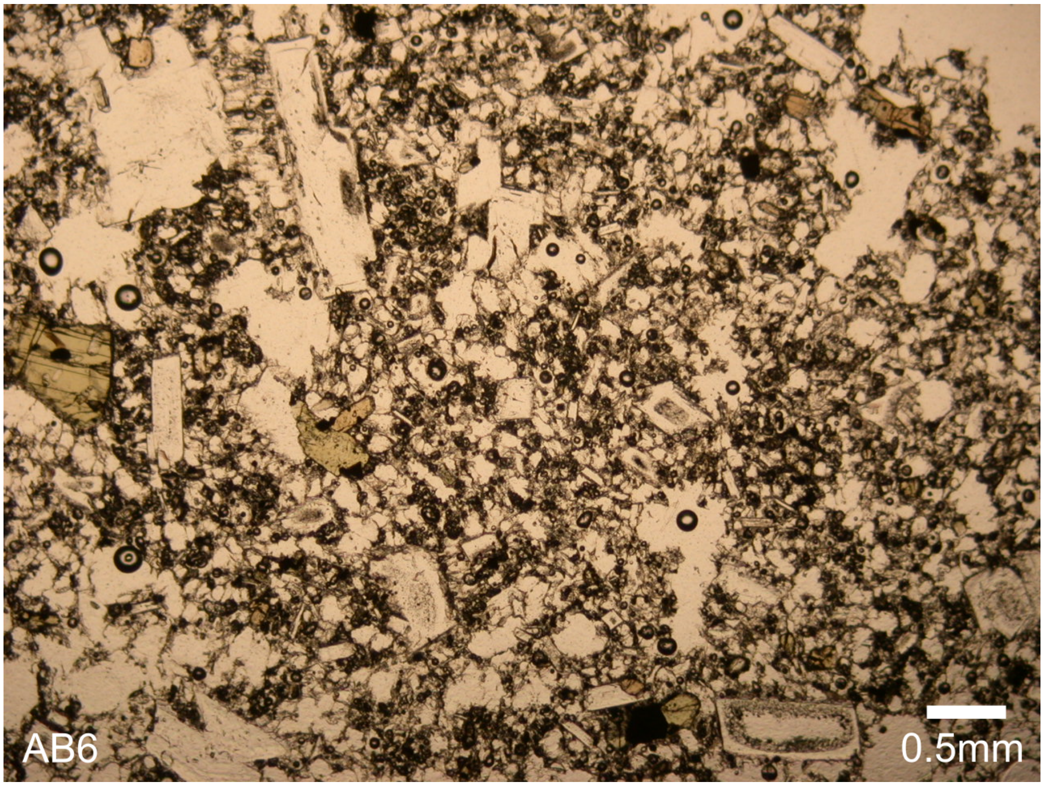

AB6 interior
Vesicles: **70%**
Phenocrysts: 14%
Plagioclase 10%
Pyroxenes 3%
Fe-Ti Oxides 1%
Groundmass: 16%

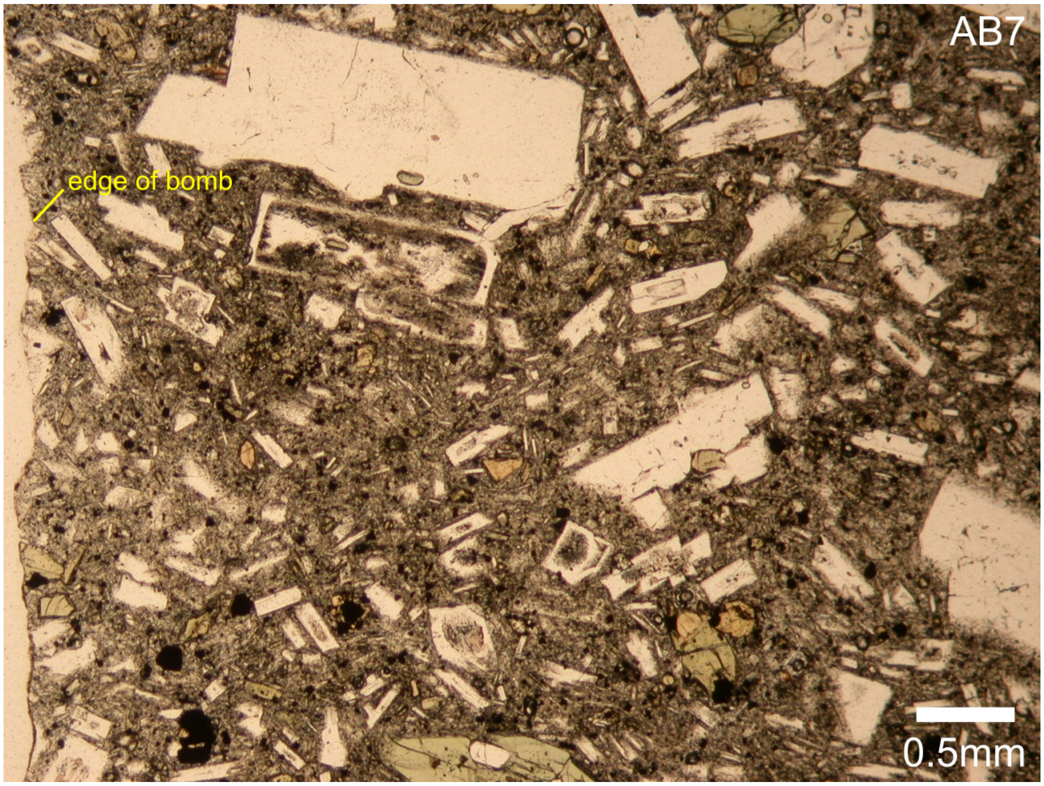

AB7 Rind
Vesicles: **0.5%**
Phenocrysts: 30%
Plagioclase 20%
Pyroxenes 7%
Fe-Ti Oxides 3%
Groundmass: 69.5%

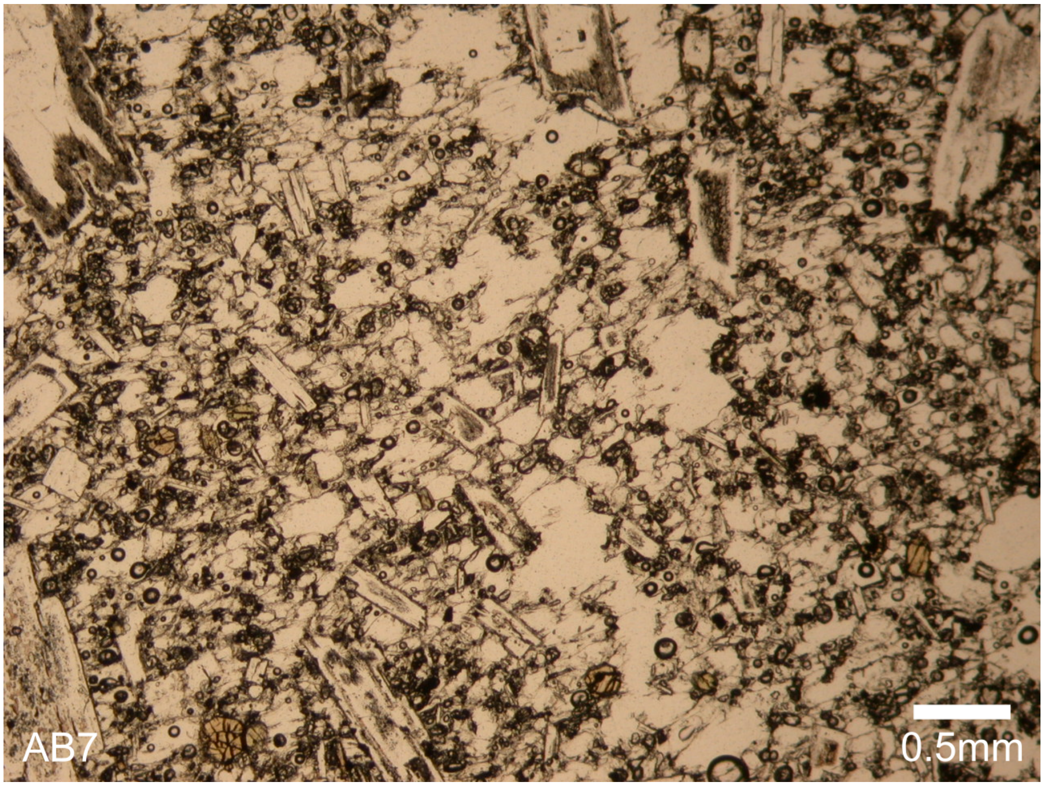

AB7 Interior
Vesicles: **80%**
Phenocrysts: 11%
Plagioclase 7%
Pyroxenes 3%
Fe-Ti Oxides 1%
Groundmass: 9%

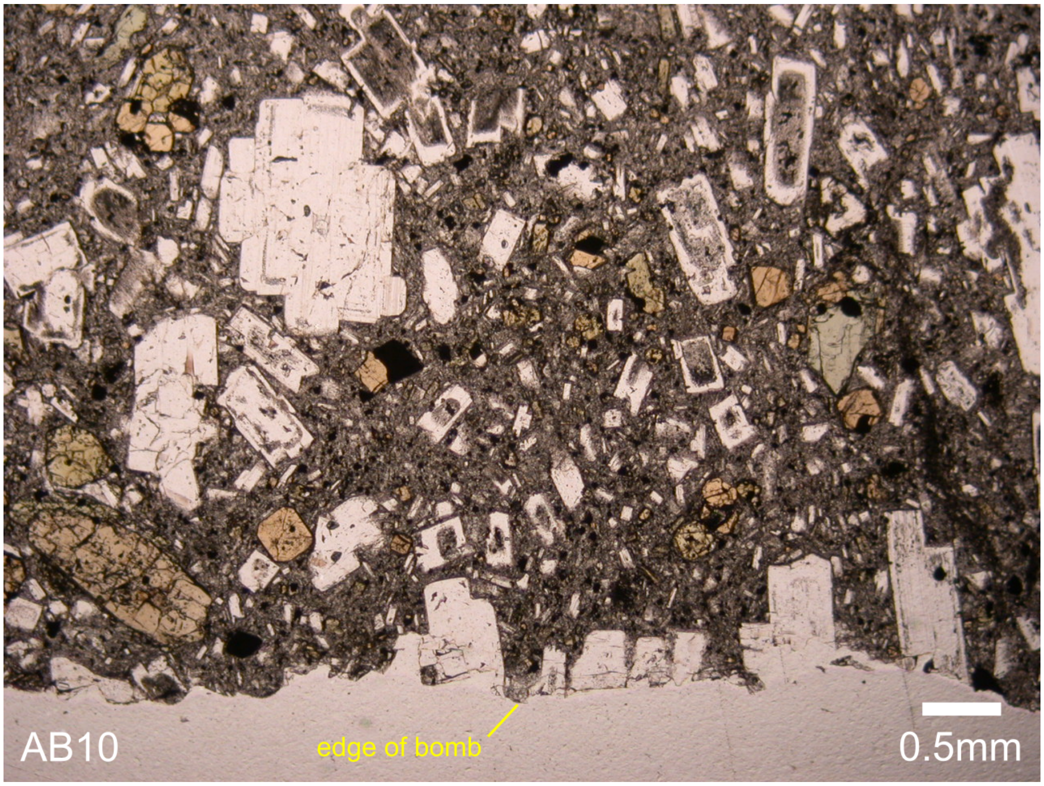

AB10 Rind
Vesicles: **0%**
Phenocrysts: 33%
Plagioclase 23%
Pyroxenes 7%
Fe-Ti Oxides 3%
Groundmass: 67%

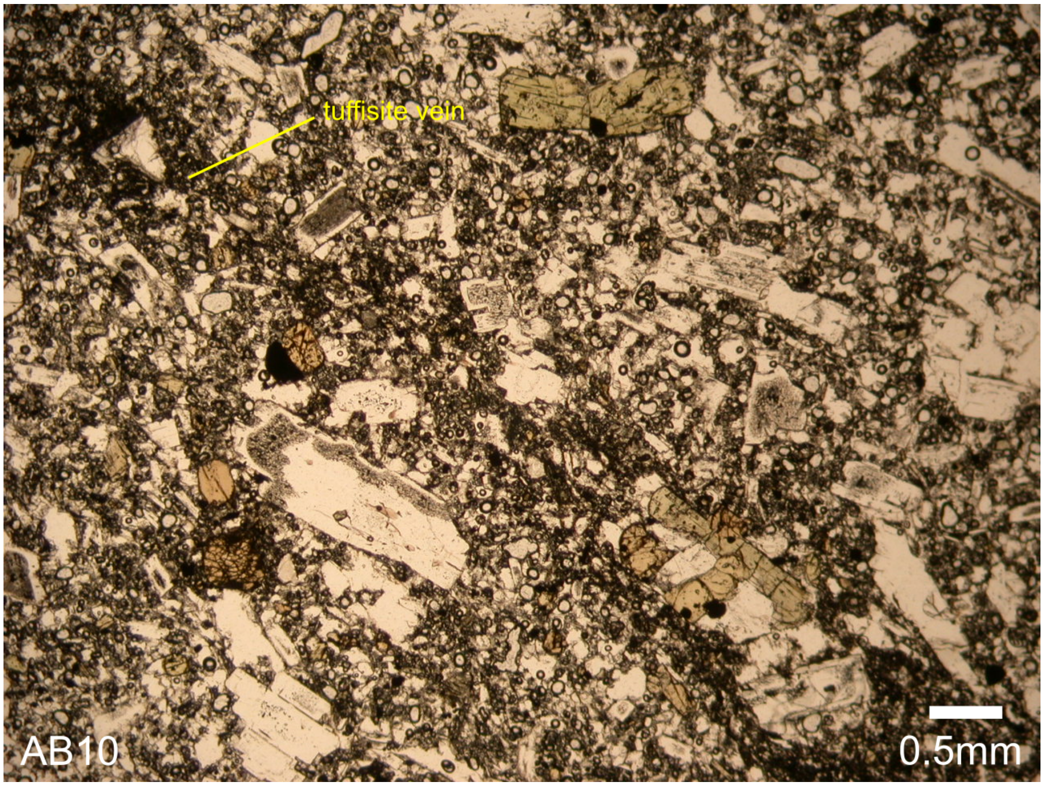

AB10 Interior
Vesicles: **60%**
Phenocrysts: 14%
Plagioclase 10%
Pyroxenes 3%
Fe-Ti Oxides 1%
Groundmass: 26%

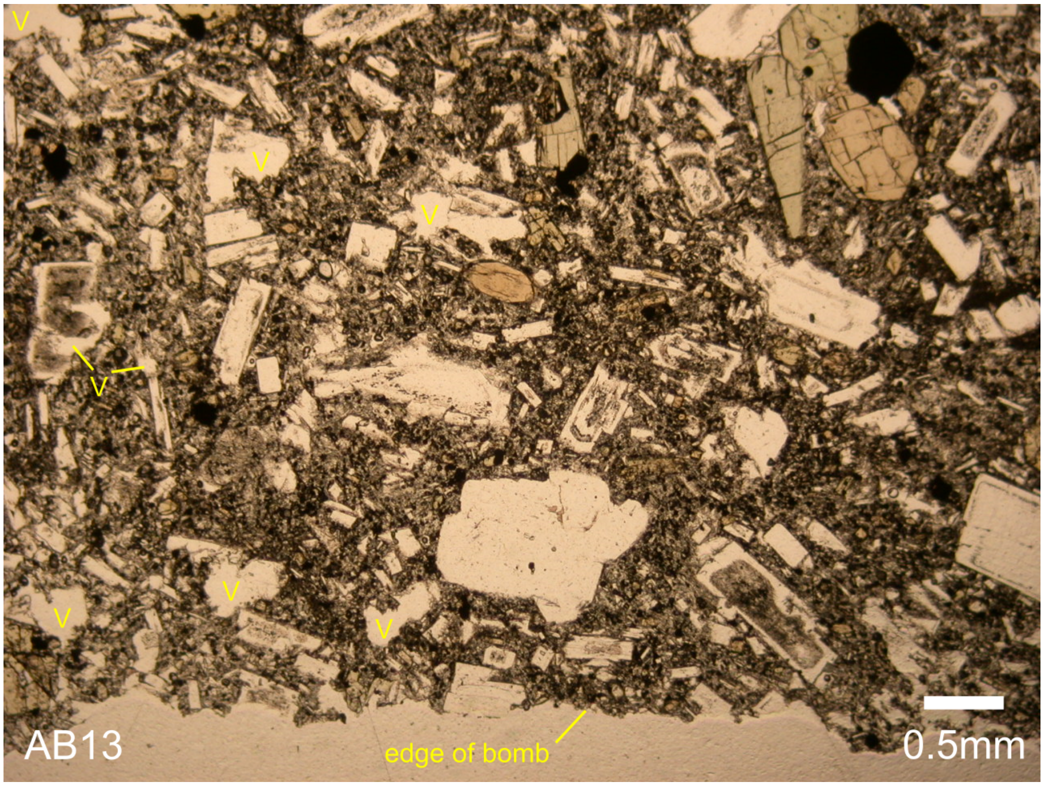

AB13 Rind
Vesicles: **0.5%**
Phenocrysts: 30%
Plagioclase 20%
Pyroxenes 7%
Fe-Ti Oxides 3%
Groundmass: 69.5%

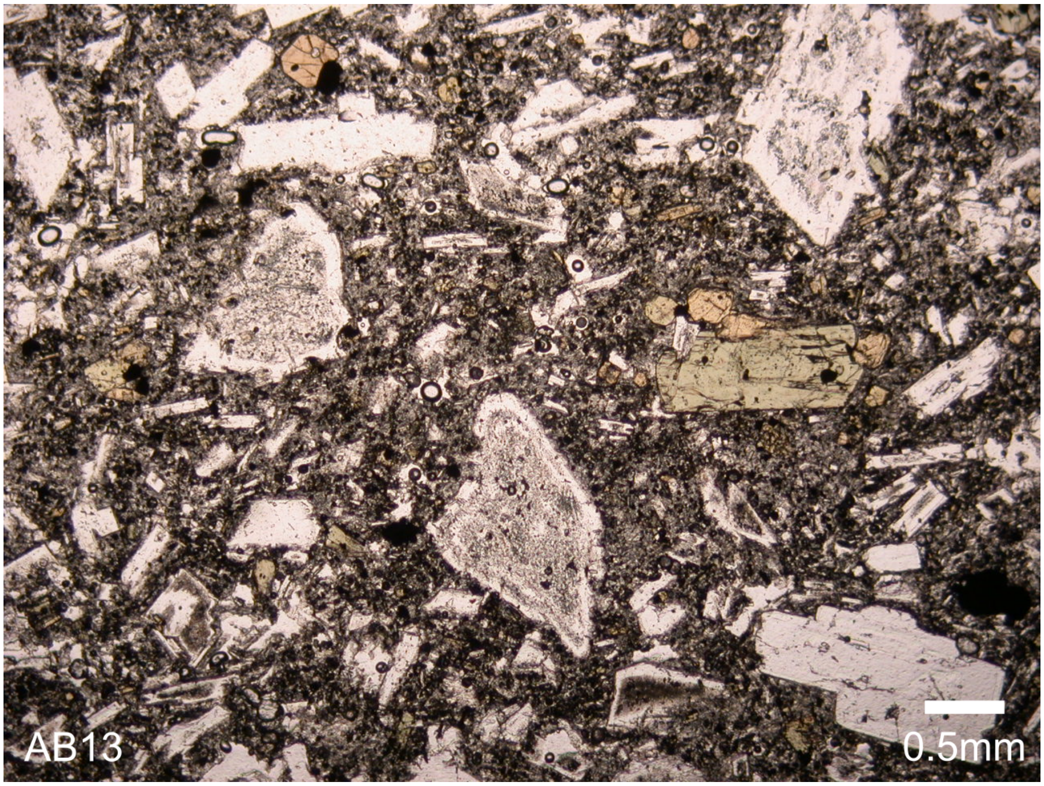

AB13 Interior
Vesicles: **40%**
Phenocrysts: 14%
Plagioclase 10%
Pyroxenes 3%
Fe-Ti Oxides 1%
Groundmass: 45%

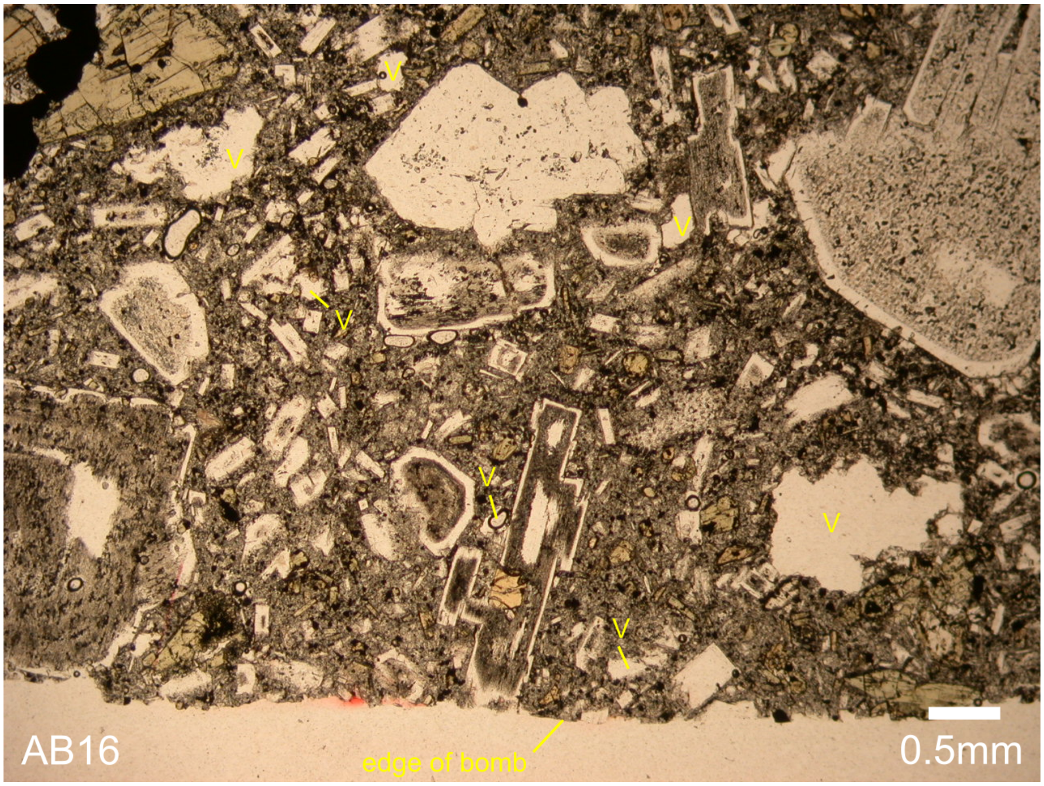

AB16 Rind
Vesicles: **10%**
Phenocrysts: 30%
Plagioclase 20%
Pyroxenes 7%
Fe-Ti Oxides 3%
Groundmass: 60%

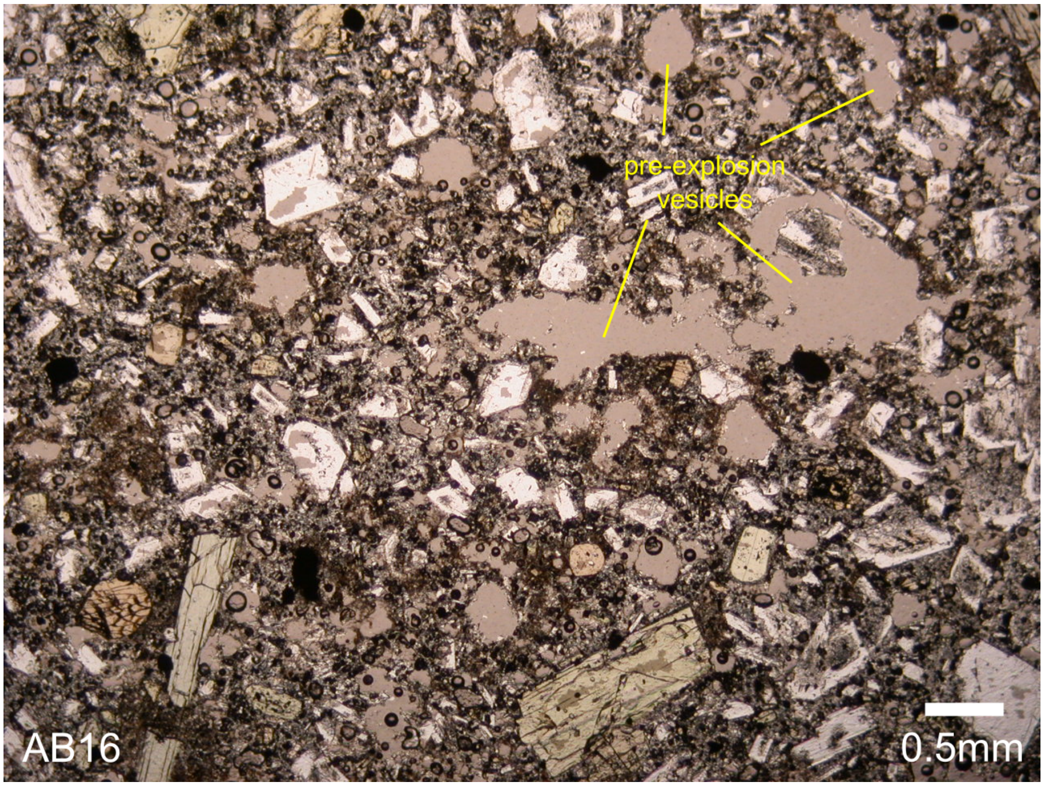

AB16 Interior
Vesicles: **60%**
Phenocrysts: 23%
Plagioclase 15%
Pyroxenes 5%
Fe-Ti Oxides 3%
Groundmass: 17%

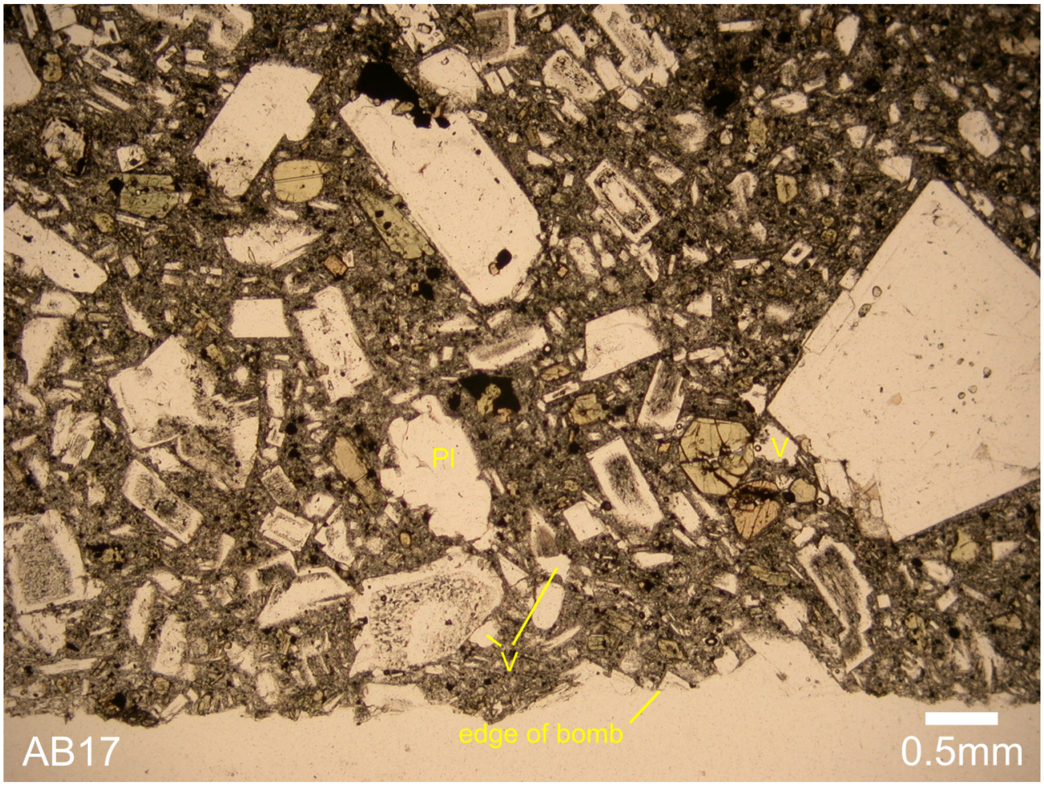

AB17 Rind
Vesicles: **0.5%**
Phenocrysts: 31%
Plagioclase 20%
Pyroxenes 7%
Fe-Ti Oxides 4%
Groundmass: 68.5%

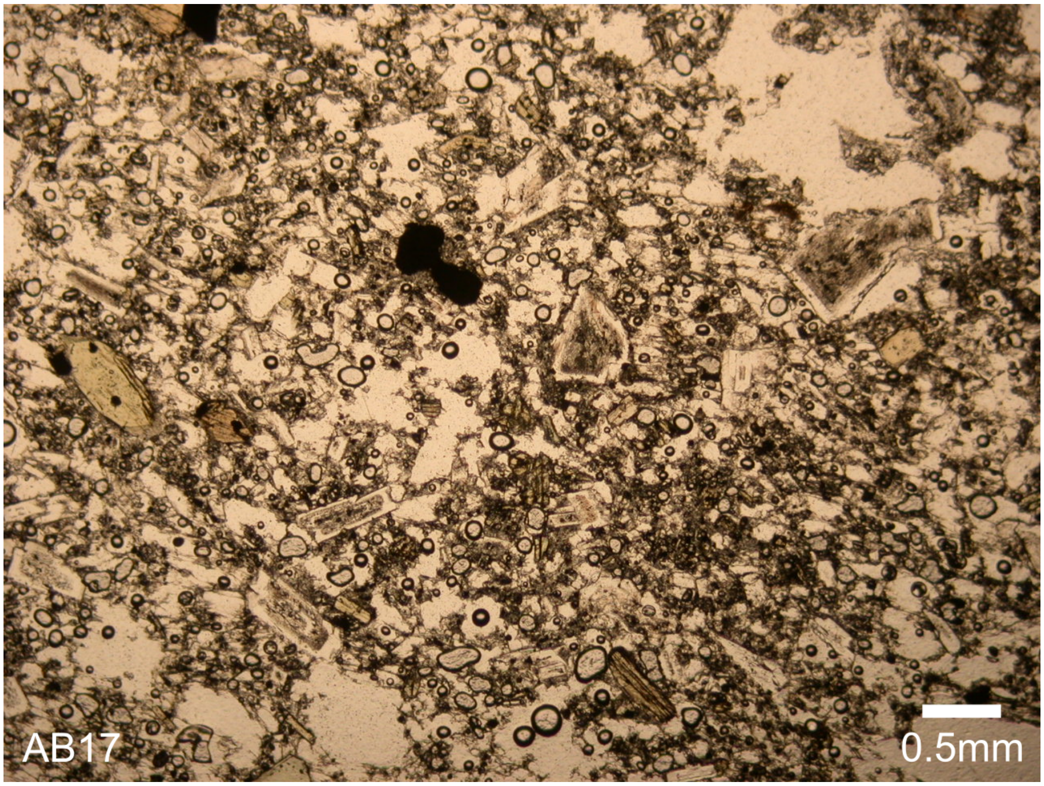

AB17 Interior
Vesicles: **80%**
Phenocrysts: 14%
Plagioclase 10%
Pyroxenes 3%
Fe-Ti Oxides 1%
Groundmass: 6%

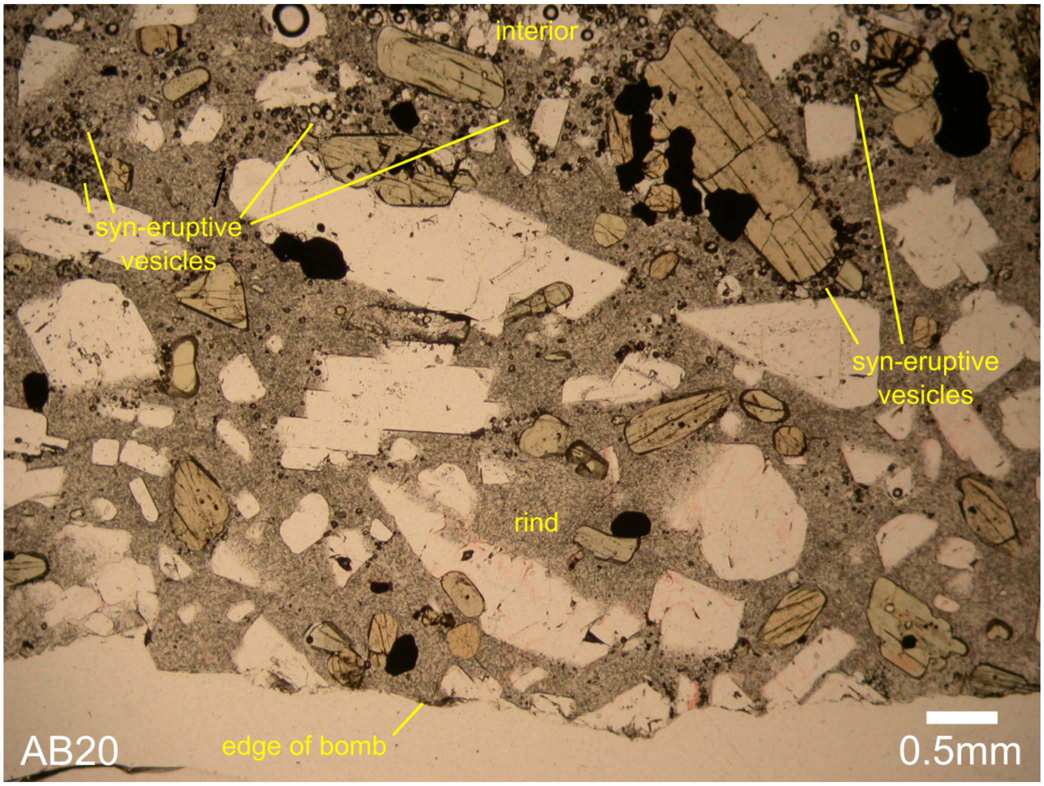

AB20 Rind
Vesicles: **0%**
Phenocrysts: 35%
Plagioclase 20%
Pyroxenes 10%
Fe-Ti Oxides 5%
Groundmass: 65%

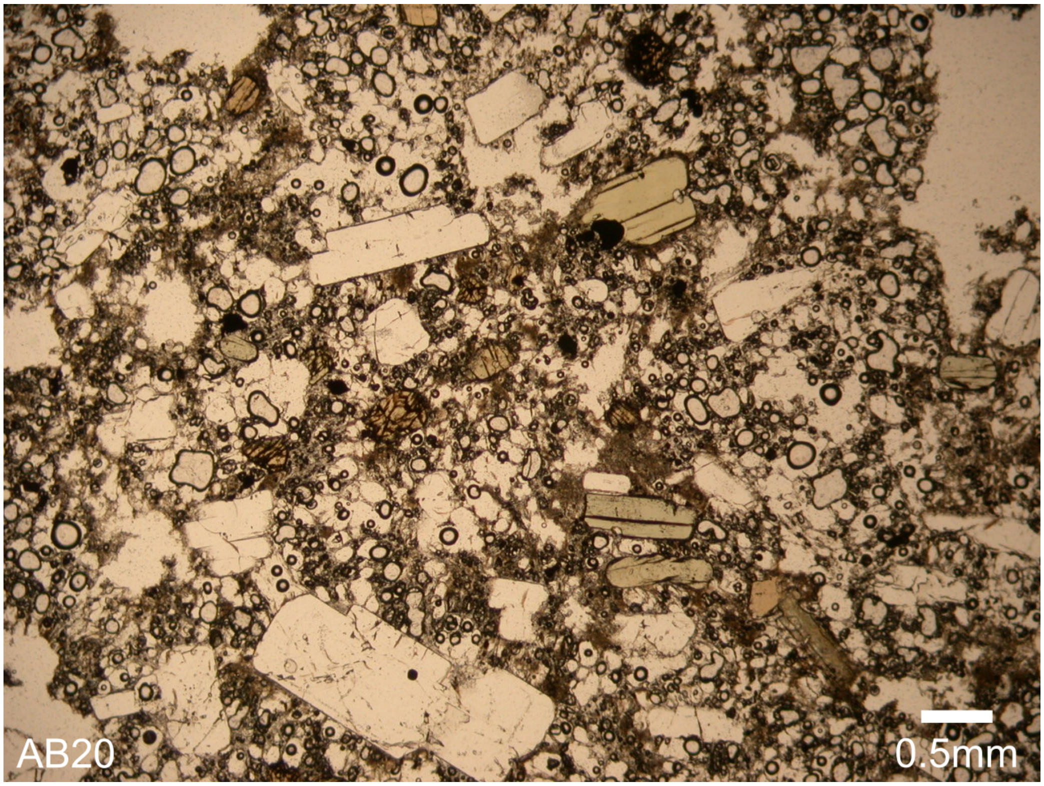

AB20 Interior
Vesicles: **70%**
Phenocrysts: 14%
Plagioclase 8%
Pyroxenes 4%
Fe-Ti Oxides 2%
Groundmass: 16%

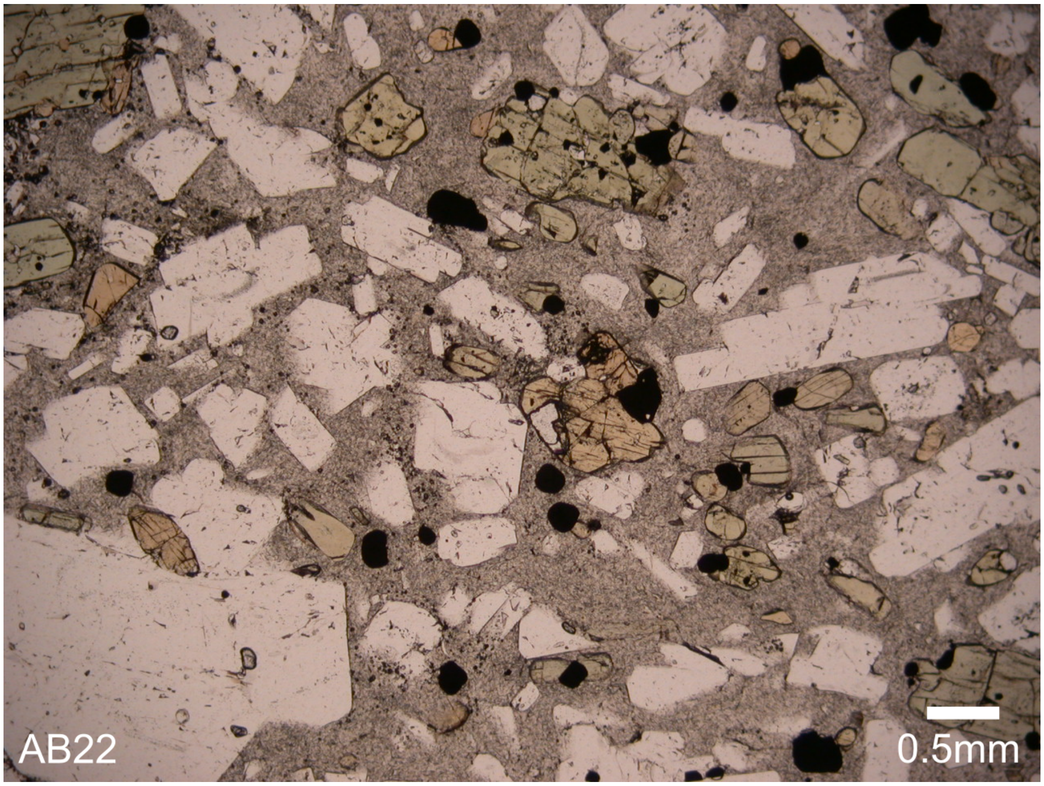

AB22 Rind
Vesicles: **0.5%**
Phenocrysts: 35%
Plagioclase 20%
Pyroxenes 10%
Fe-Ti Oxides 5%
Groundmass: 64.5%

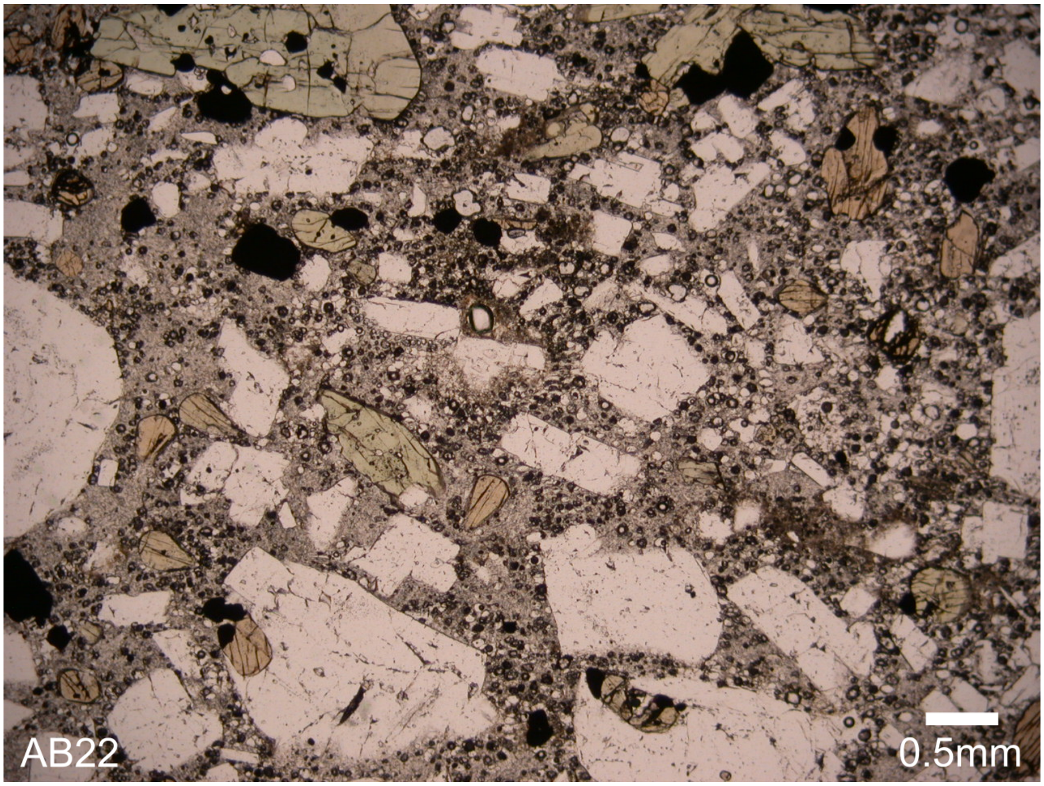

AB22 Interior
Vesicles: **40%**
Phenocrysts: 26%
Plagioclase 15%
Pyroxenes 7%
Fe-Ti Oxides 4%
Groundmass: 34%

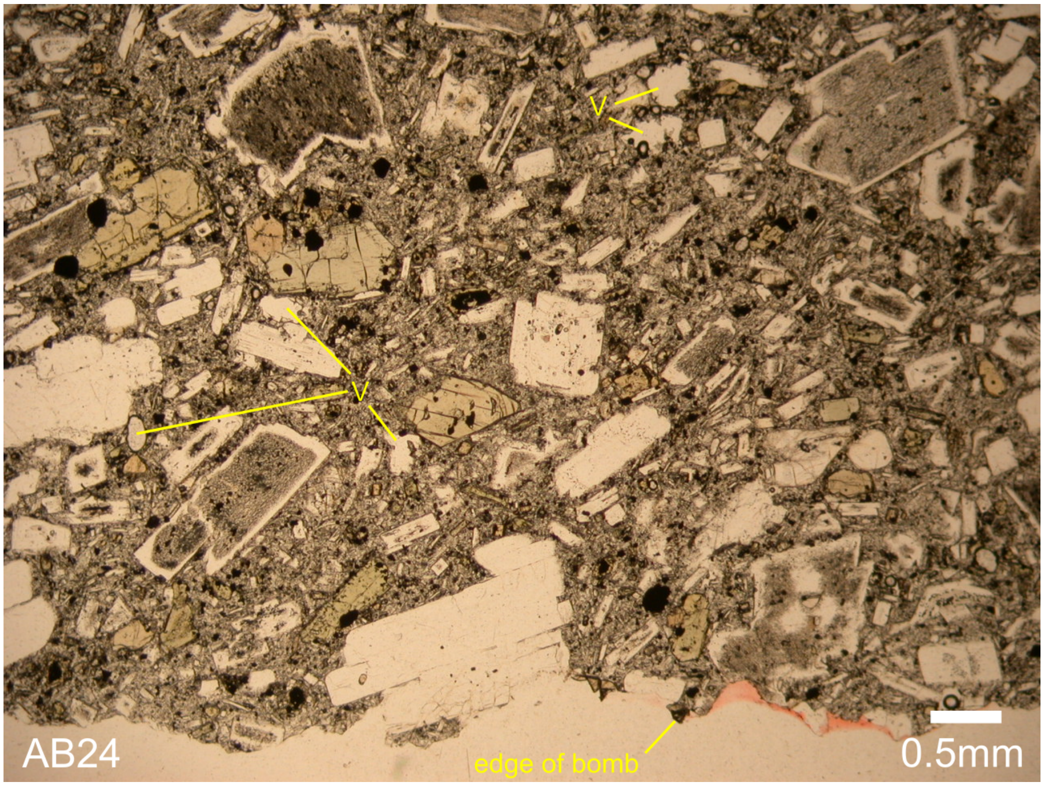

AB24 Rind
Vesicles: **3%**
Phenocrysts: 28%
Plagioclase 15%
Pyroxenes 10%
Fe-Ti Oxides 3%
Groundmass: 69%

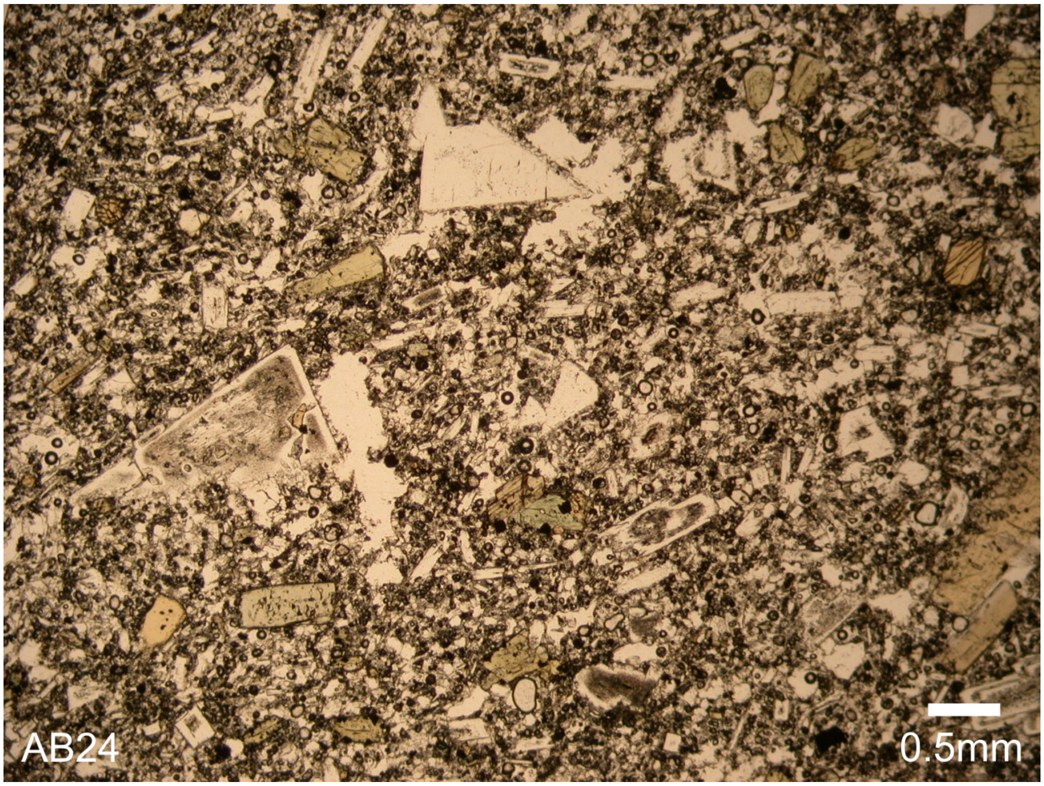

AB24 Interior
Vesicles: **50%**
Phenocrysts: 14%
Plagioclase 10%
Pyroxenes 3%
Fe-Ti Oxides 1%
Groundmass: 36%

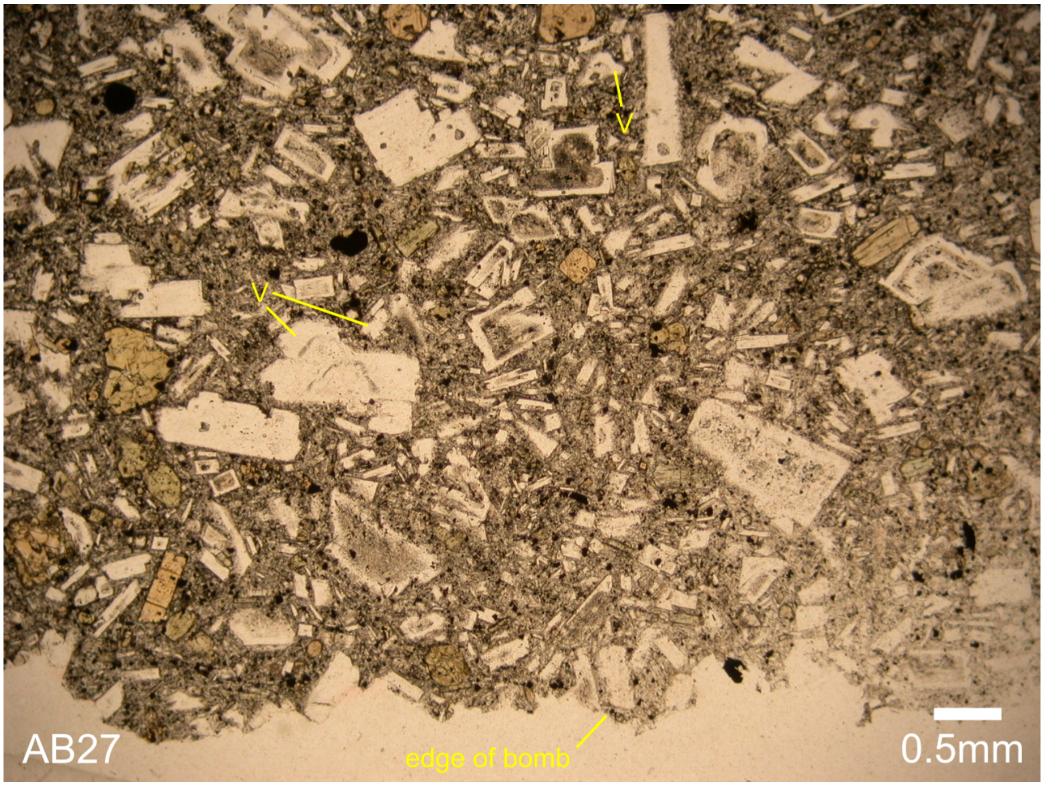

AB27 rind
Vesicles: **0.5%**
Phenocrysts: 30%
Plagioclase 20%
Pyroxenes 7%
Fe-Ti Oxides 3%
Groundmass: 69.5%

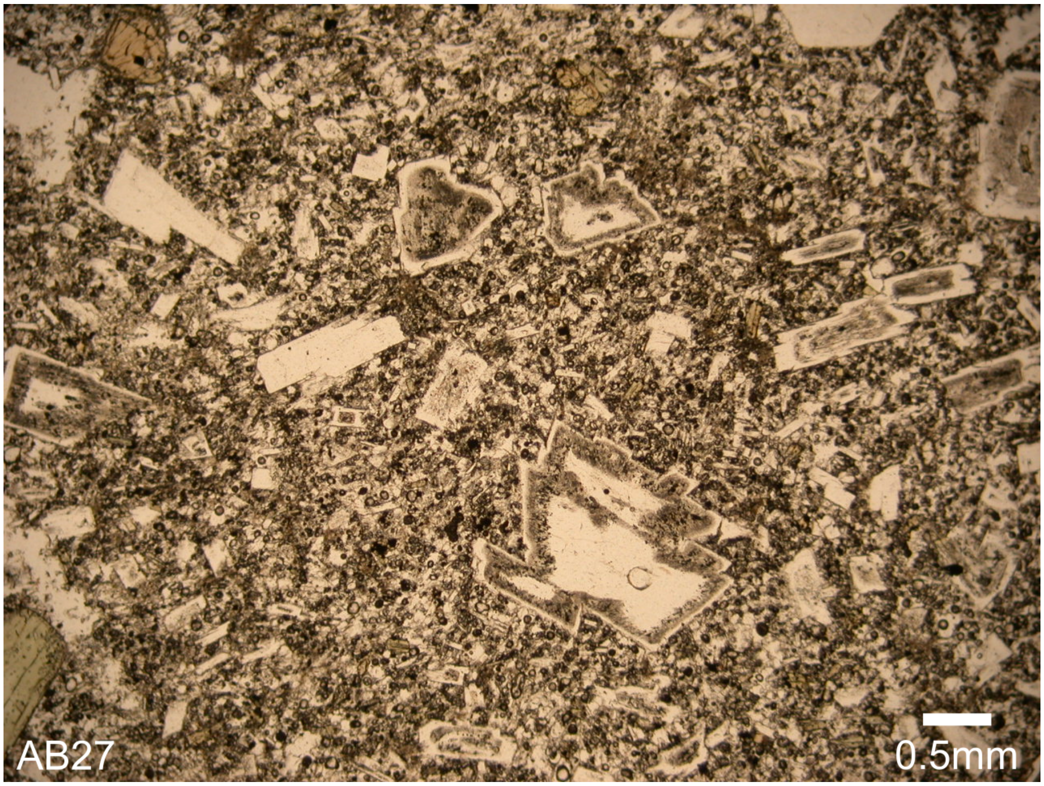

AB27 Interior
Vesicles: **65%**
Phenocrysts: 14%
Plagioclase 10%
Pyroxenes 3%
Fe-Ti Oxides 1%
Groundmass: 21%

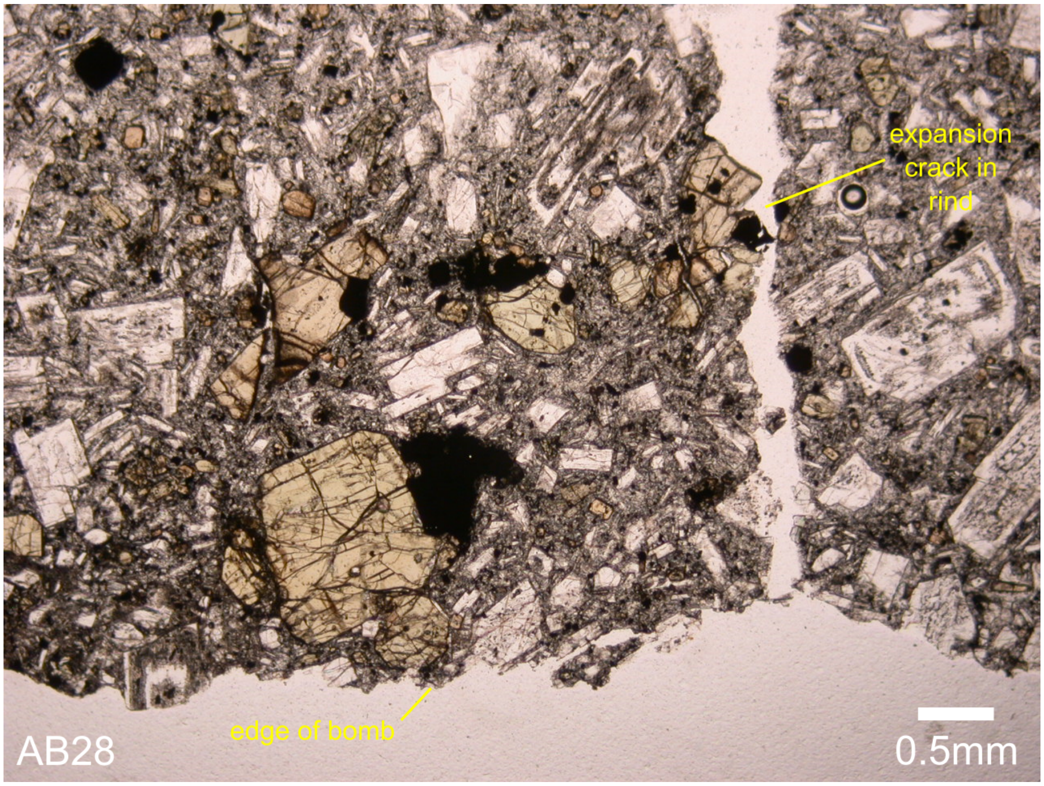

AB28 Rind
Vesicles: **0.5%**
Phenocrysts: 30%
Plagioclase 20%
Pyroxenes 7%
Fe-Ti Oxides 3%
Groundmass: 69.5%

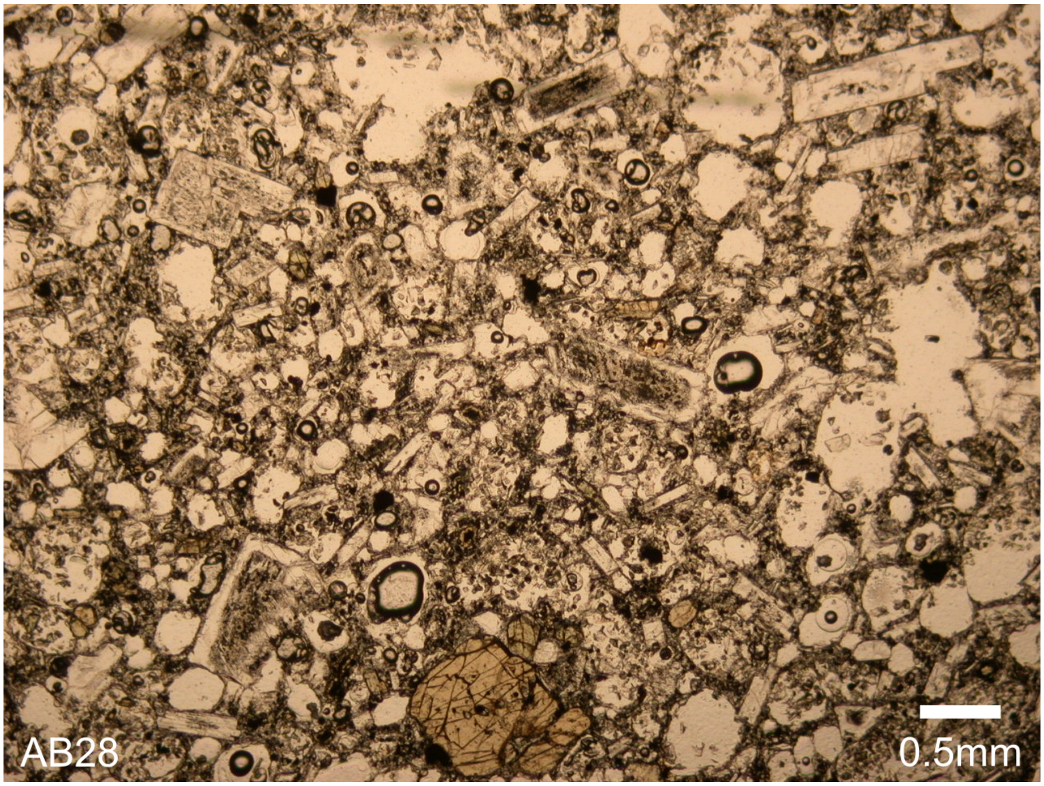

AB28 Interior
Vesicles: **70%**
Phenocrysts: 15%
Plagioclase 10%
Pyroxenes 3%
Fe-Ti Oxides 2%
Groundmass: 15%

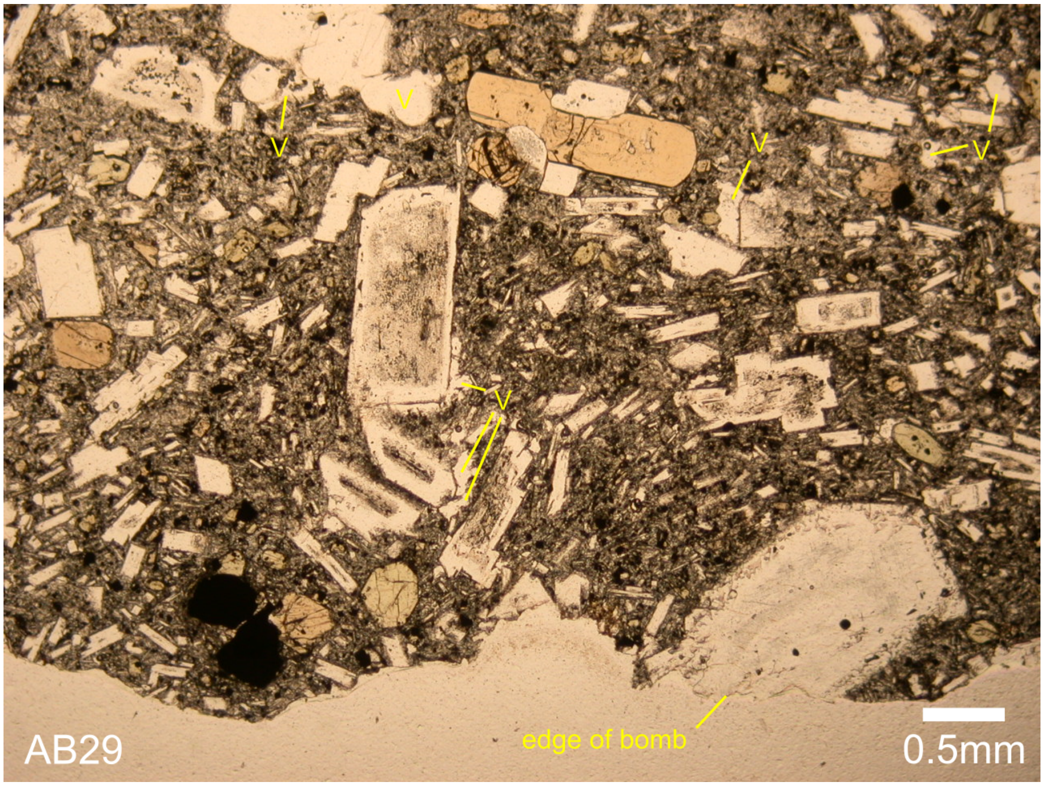

AB29 rind
Vesicles: **0.5%**
Phenocrysts: 28%
Plagioclase 20%
Pyroxenes 5%
Fe-Ti Oxides 3%
Groundmass: 71.5%

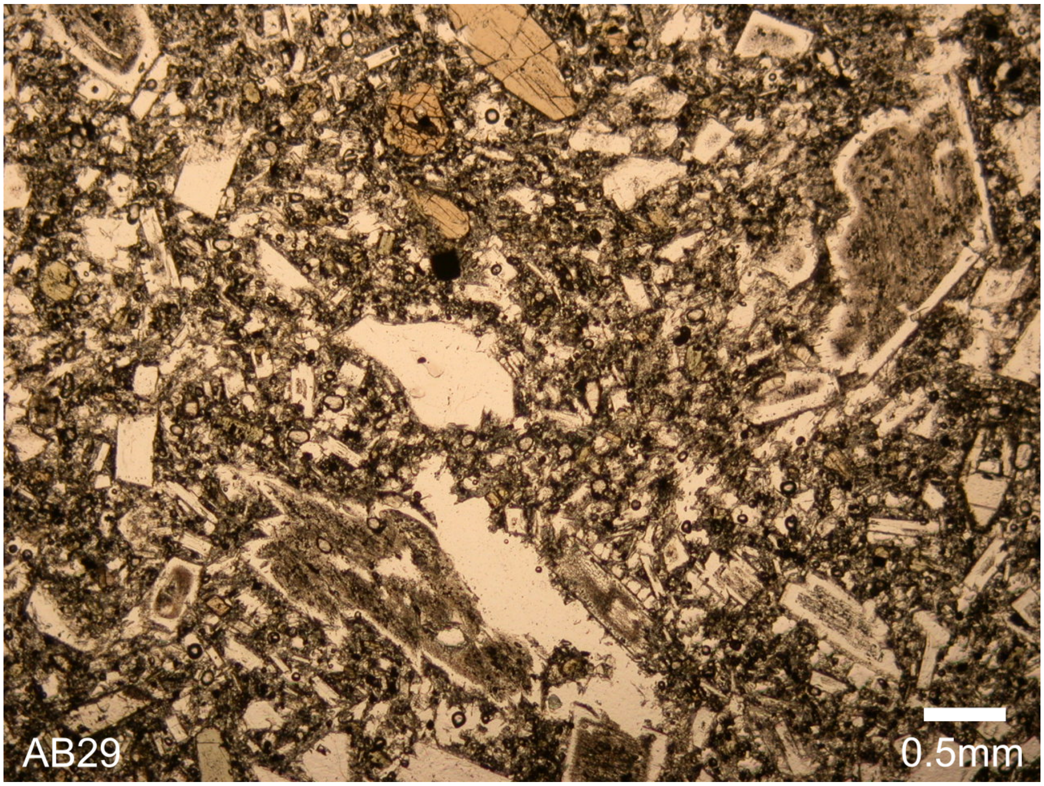

AB29 Interior
Vesicles: **50%**
Phenocrysts: 15%
Plagioclase 10%
Pyroxenes 3%
Fe-Ti Oxides 2%
Groundmass: 35%

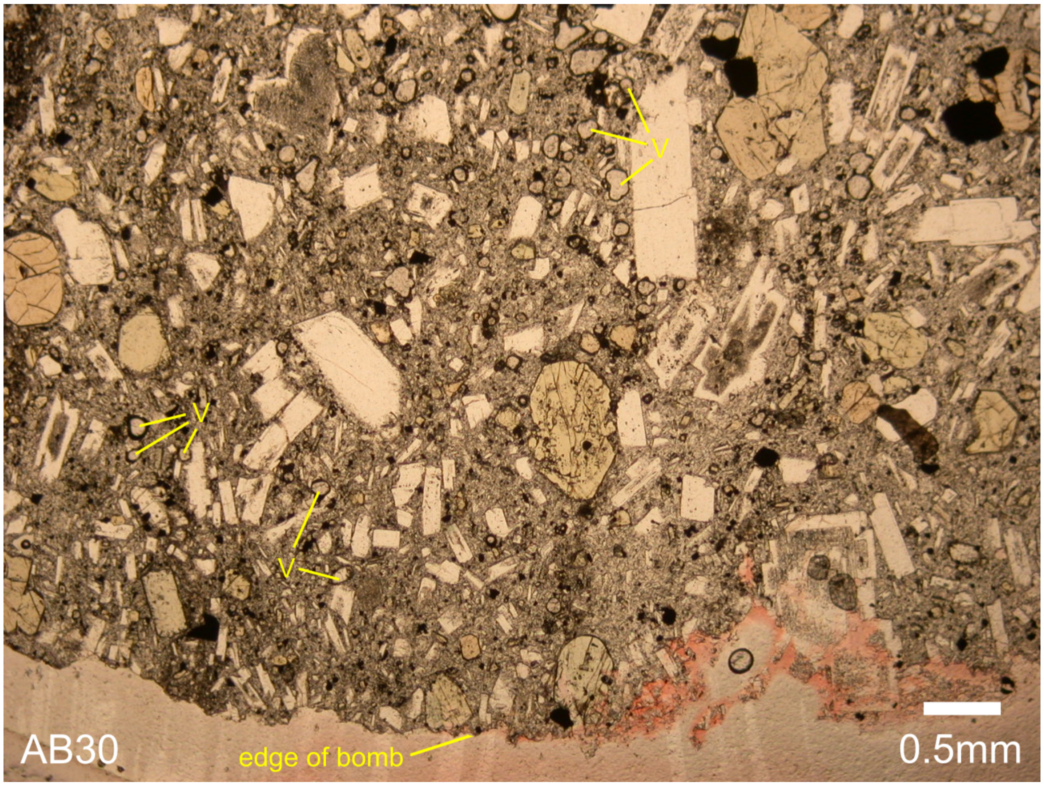

AB30 Rind
Vesicles: **0%**
Phenocrysts: 30%
Plagioclase 20%
Pyroxenes 7%
Fe-Ti Oxides 3%
Groundmass: 70%

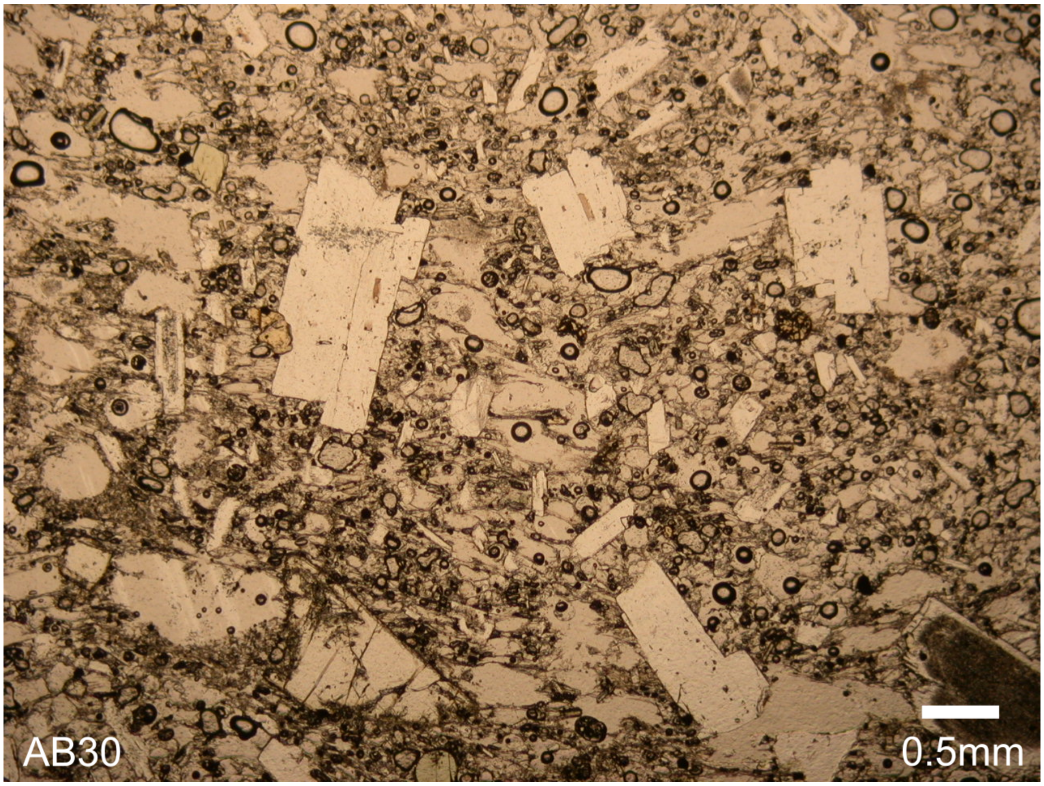

AB30 Interior
Vesicles: **80%**
Phenocrysts: 10.5%
Plagioclase 7%
Pyroxenes 3%
Fe-Ti Oxides 0.5%
Groundmass: 9.5%

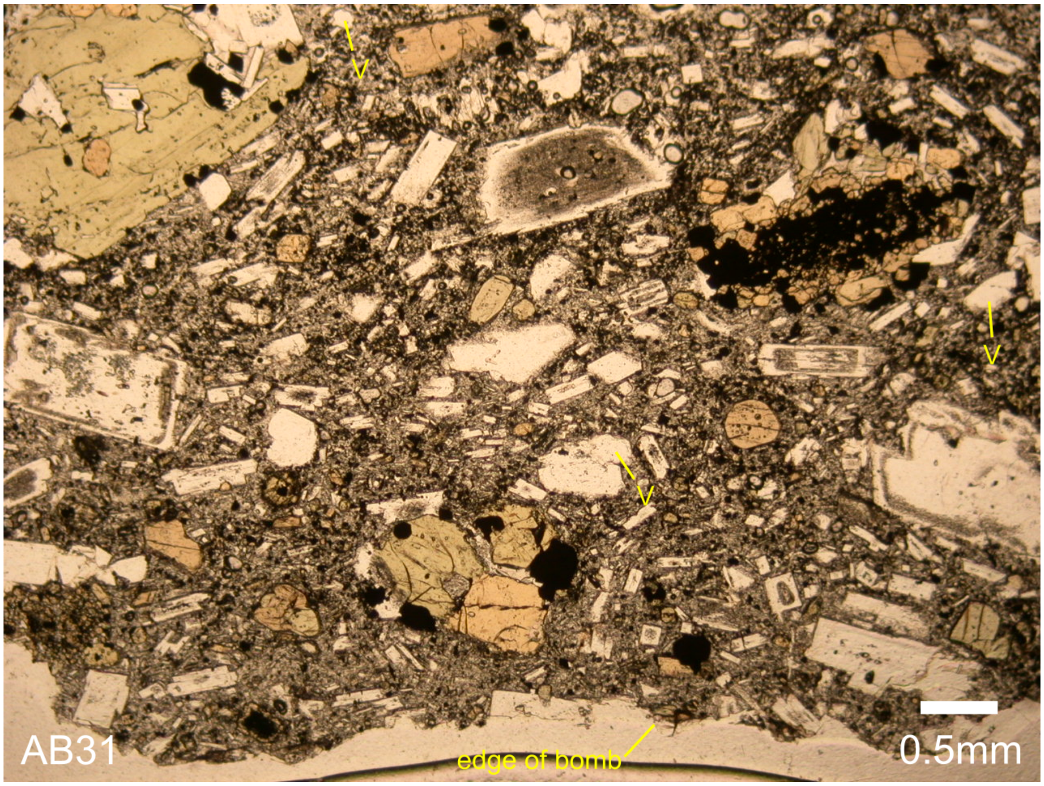

AB31 Rind
Vesicles: **0%**
Phenocrysts: 30%
Plagioclase 20%
Pyroxenes 7%
Fe-Ti Oxides 3%
Groundmass: 70%

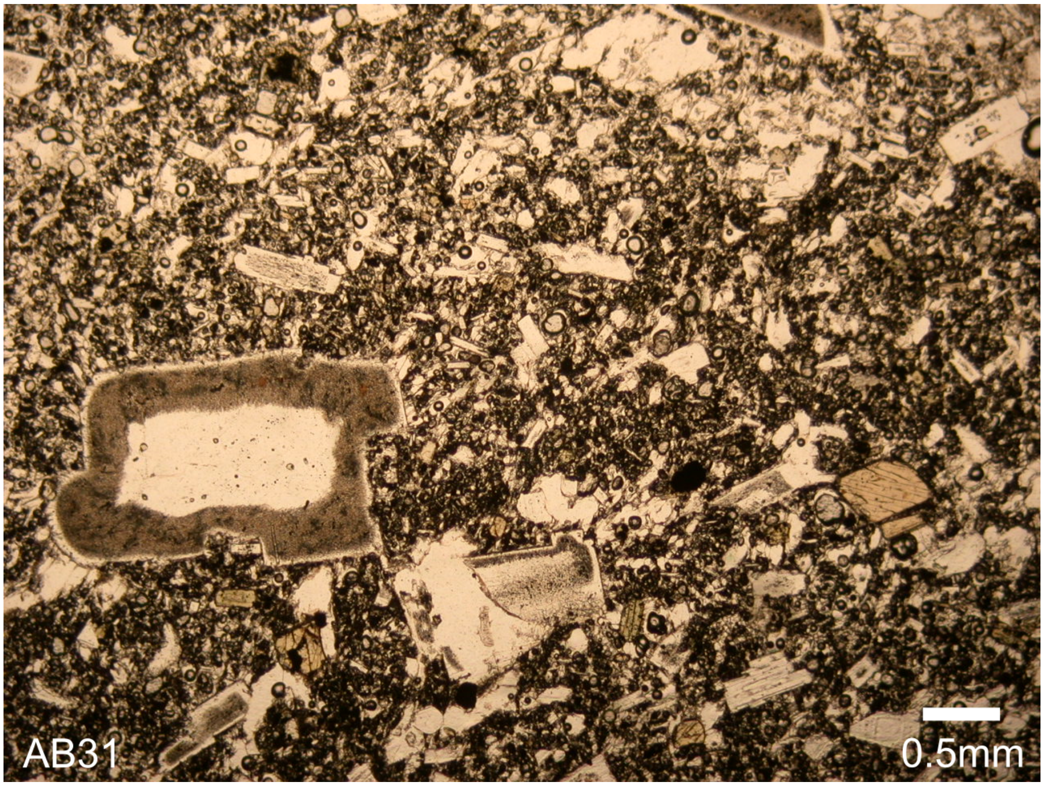

AB31 Interior
Vesicles: **80%**
Phenocrysts: 11%
Plagioclase 7%
Pyroxenes 3%
Fe-Ti Oxides 1%
Groundmass: 9%

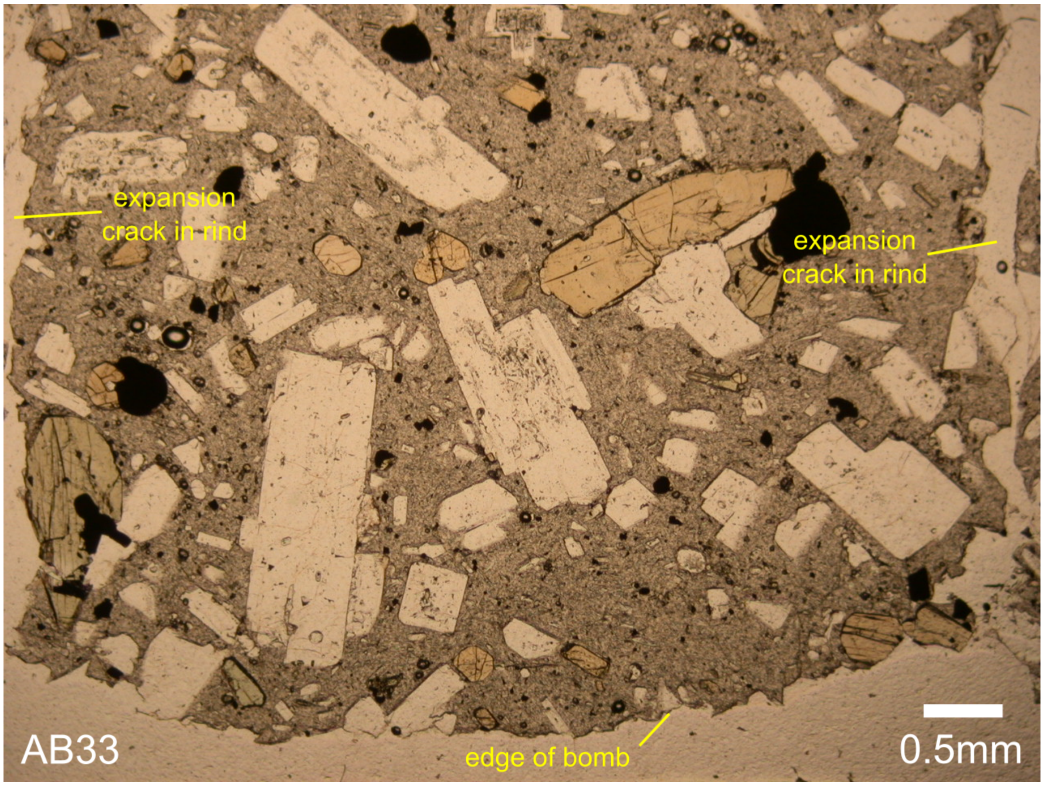

AB33 Rind
Vesicles: **1%**
Phenocrysts: 28%
Plagioclase 20%
Pyroxenes 5%
Fe-Ti Oxides 3%
Groundmass: 71%

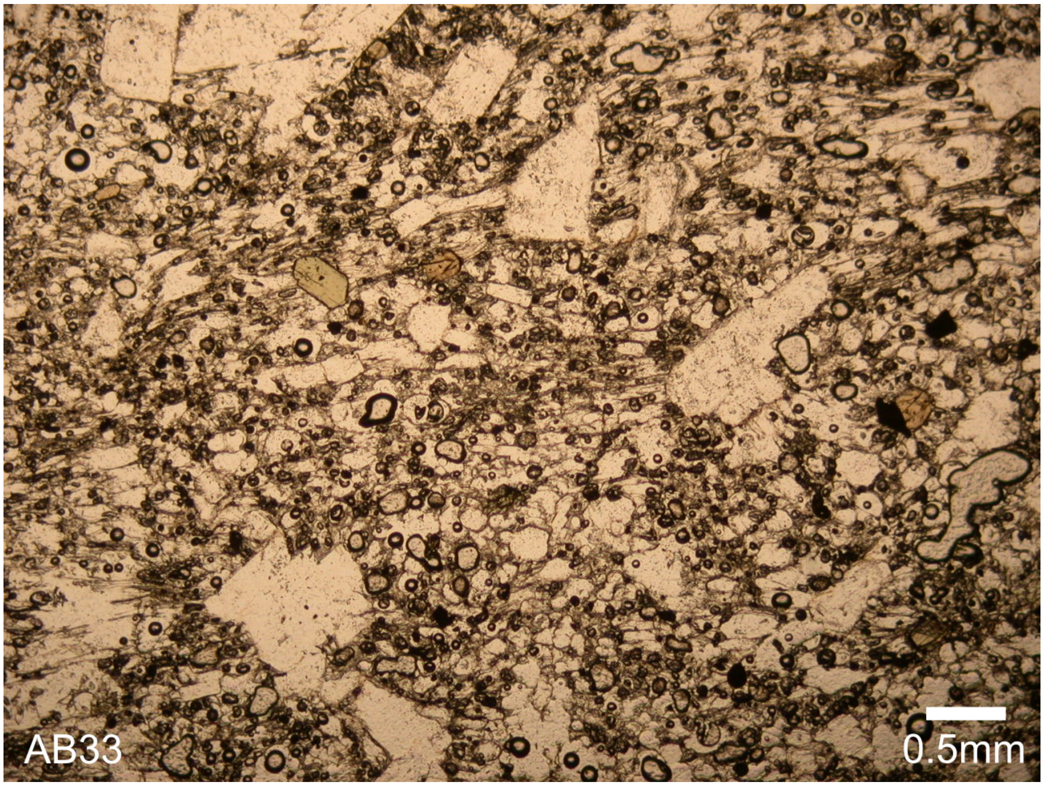

AB33 Interior
Vesicles: **80%**
Phenocrysts: 13%
Plagioclase 7%
Pyroxenes 5%
Fe-Ti Oxides 1%
Groundmass: 7%

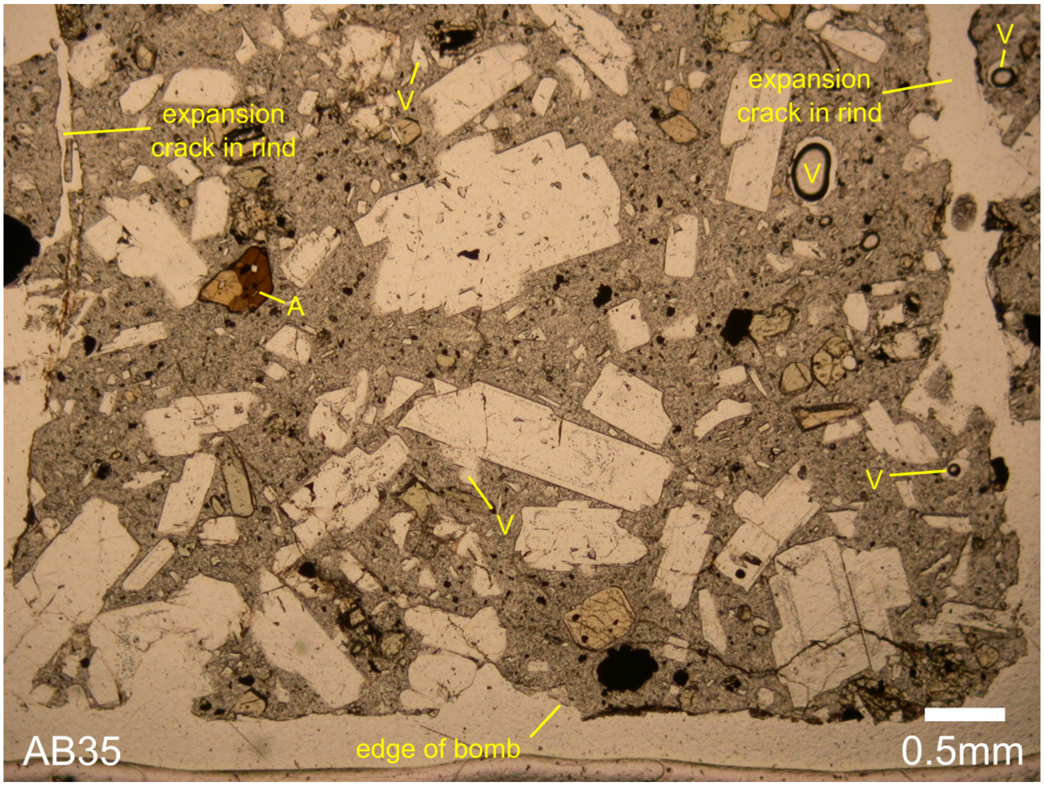

AB35 Rind
Vesicles: **0.5%**
Phenocrysts: 30%
Plagioclase 20%
Pyroxenes 7%
Fe-Ti Oxides 3%
Groundmass: 69.5%

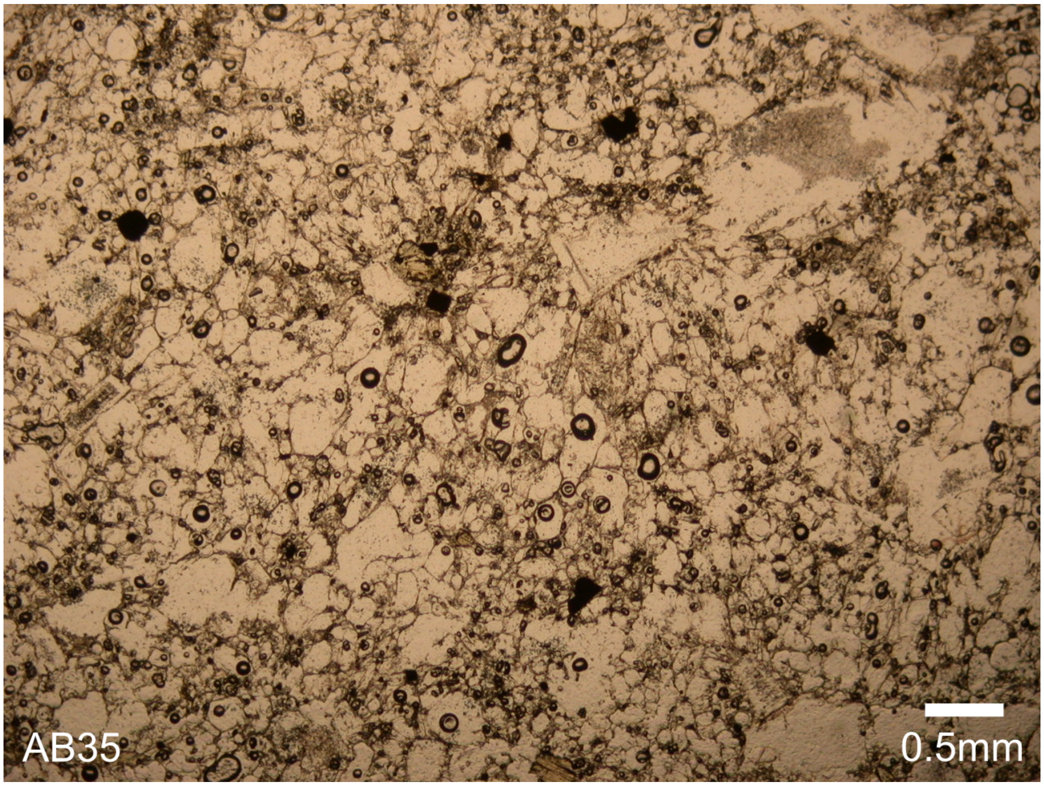

AB35 Interior
Vesicles: **85%**
Phenocrysts: 7%
Plagioclase 5%
Pyroxenes 1%
Fe-Ti Oxides 1%
Groundmass: 8%
