# Supplementary material for: Textural and geochemical constraints on andesitic plug emplacement prior to the 2004–2010 vulcanian explosions at Galeras volcano, Colombia
Source: Bull Volcanol. 2018 Dec 7;81(1):1. doi: 10.1007/s00445-018-1260-y (PMC6383983; doi:10.1007/s00445-018-1260-y)
Supplement: Supplementary file 5 — Supplemental figures (PDF 4.62 MB) [file 445_2018_1260_MOESM5_ESM.pdf]

Online Resource 5

Textural and geochemical constraints on andesitic plug emplacement prior to the 2004-2010 vulcanian explosions at Galeras volcano, Colombia

Bulletin of Volcanology

Amelia A. Bain, Eliza S. Calder, Joaquín A. Cortés, Gloria Patricia Cortés J., Susan C. Loughlin

This online resource provides supplemental figures in the order mentioned in the text.

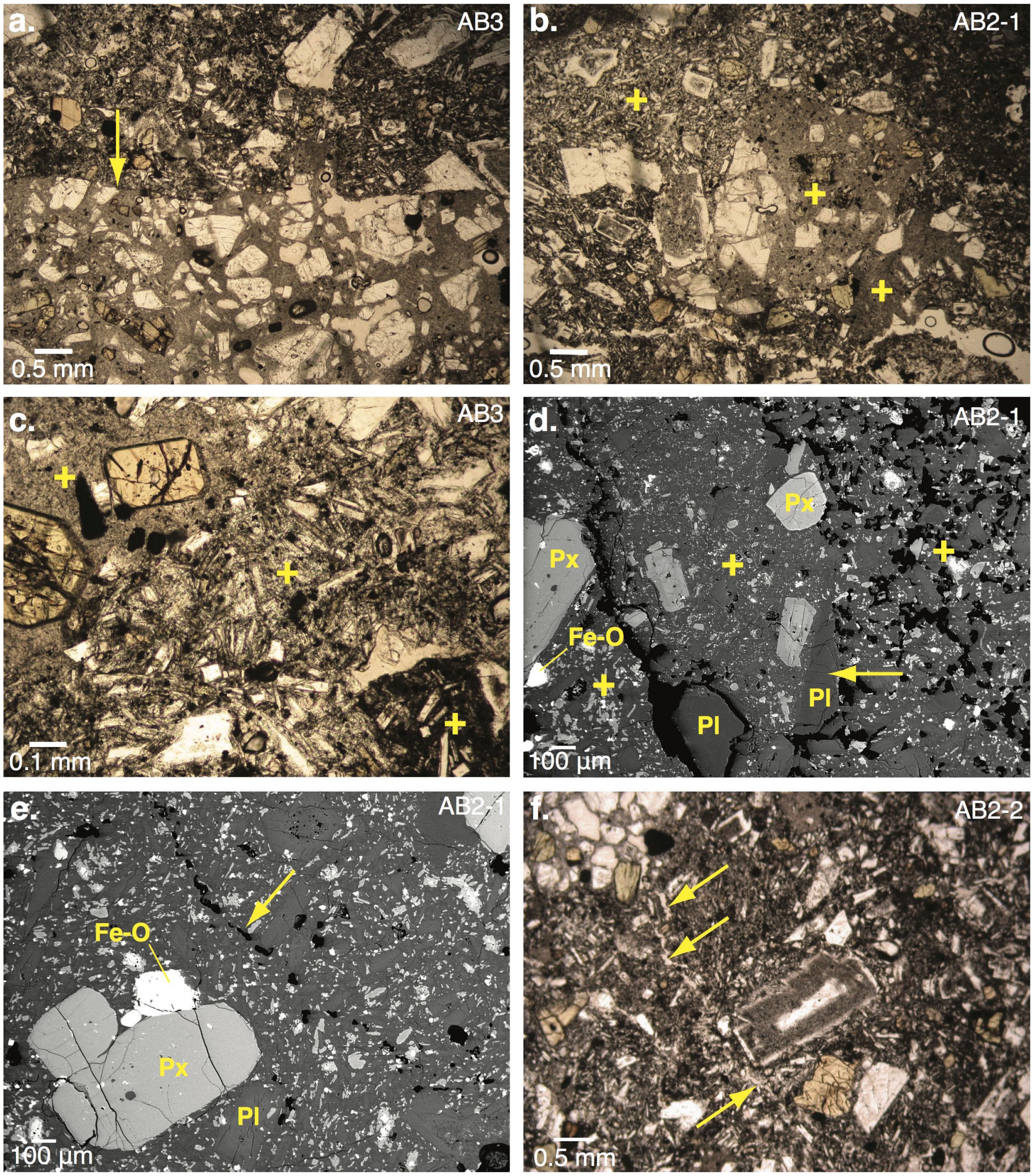

S 1: Photomicrographs and BSE images of features in tuffisite veins observed in dense bombs. Void space appears black and solid and glass phases appear light grey-white in BSE images. **a.** Sharp boundaries between andesitic clasts (shown by yellow arrow) **b.** Annealed andesitic clasts with contrasting textures (clasts marked by yellow crosses) **c.** Cryptic boundary between annealed andesitic clasts (clasts marked by yellow crosses) **d.** Linear voids between clasts with contrasting vesicularity **e.** Linear train of voids marking the annealed boundary between clasts (indicated by the yellow arrow) **f.** Folded void marking the boundary between clasts (indicated by the yellow arrow). Px = pyroxene crystal, Pl = plagioclase crystal, Fe-O = Fe-Ti oxide crystal.

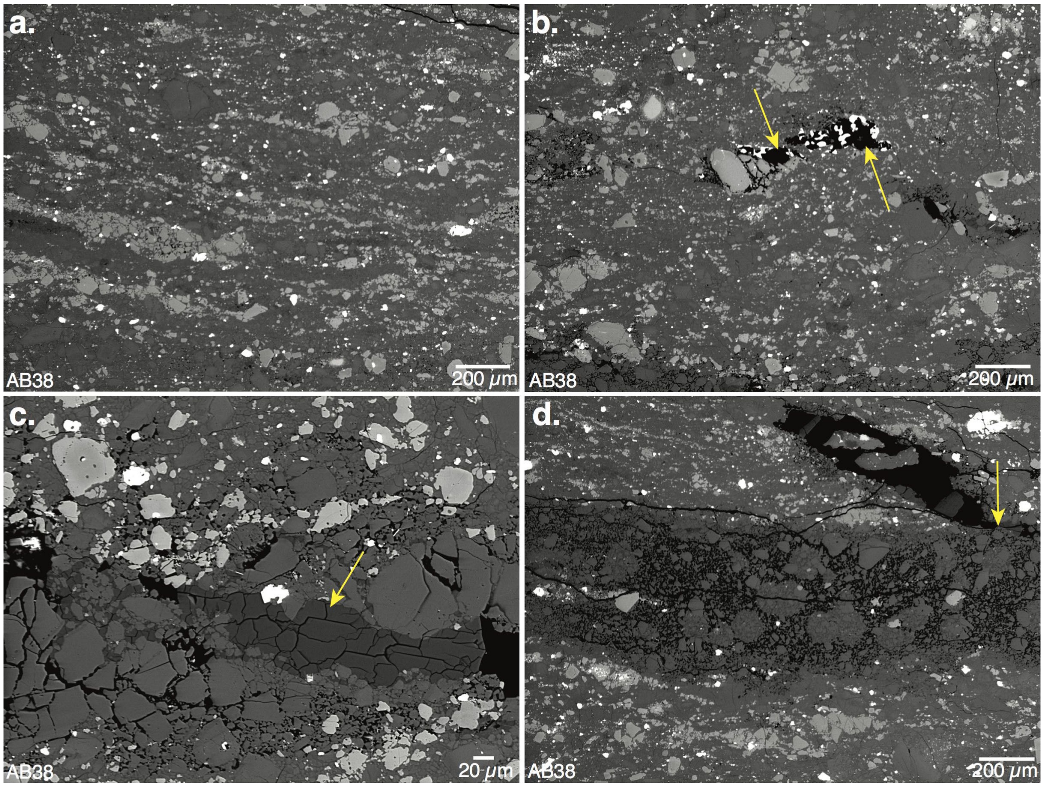

S2: **a.** BSE image of a cataclasite band cutting through sample AB38. The main phenocryst phases (plagioclase, pyroxenes and Fe-Ti oxides) are shattered and sheared. **b.** Voids exist in the pressure shadows surrounding phenocrysts within the cataclasite band. **c.** Higher porosity zones within the cataclasite band are often filled with cristobalite (dark grey phase indicated by the yellow arrow) **d.** Cross-cutting tuffisite veins have higher porosity than the surrounding cataclasite (cross-cutting relationship indicated by the yellow arrow).

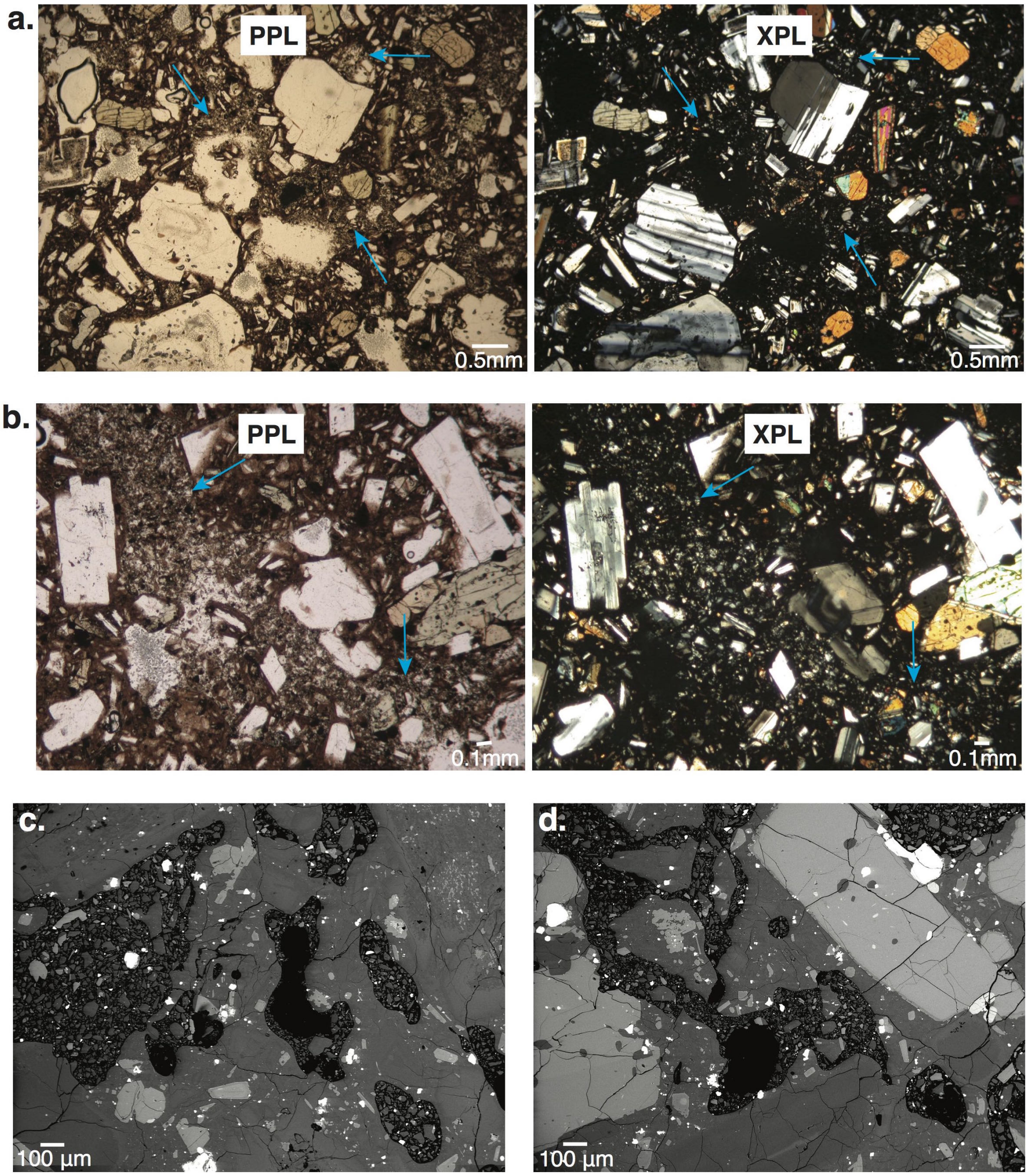

S 3: **a-b.** Plane polarised light (PPL) and cross-polarised light (XPL) images of thin sections showing tuffisite vein material infilling vesicles in scoriaceous bombs as well as fractures between vesicles (indicated by the blue arrows). **c.** BSE image of tuffisite material partially and completely infilling vesicles in scoriaceous bombs. **d.** BSE image of tuffisite material infilling a fracture between vesicles in a scoriaceous bomb.

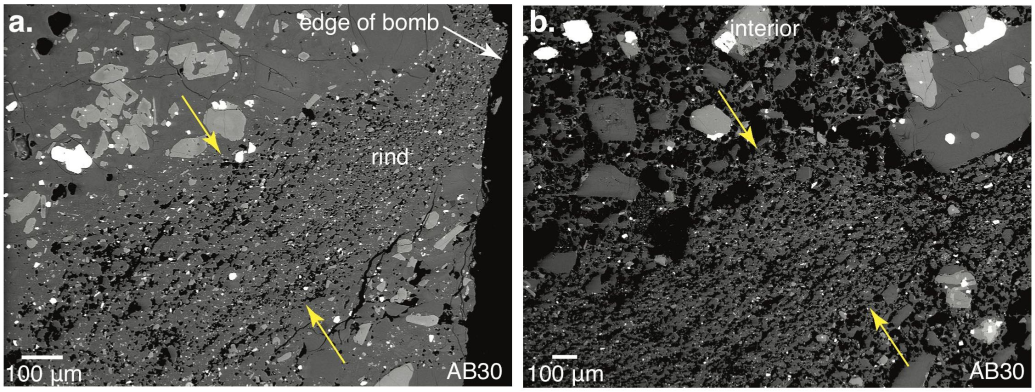

S 4: **a.** BSE (back-scattered electron) image of a tuffisite vein cutting through the rind of an inflated bomb, bracketted between the yellow arrows. The porosity of the vein is higher than that of the rind. **b.** BSE image of the same tuffisite vein cutting through the interior of an inflated bomb. The porosity of the vein is lower than that of the vesicular interior.

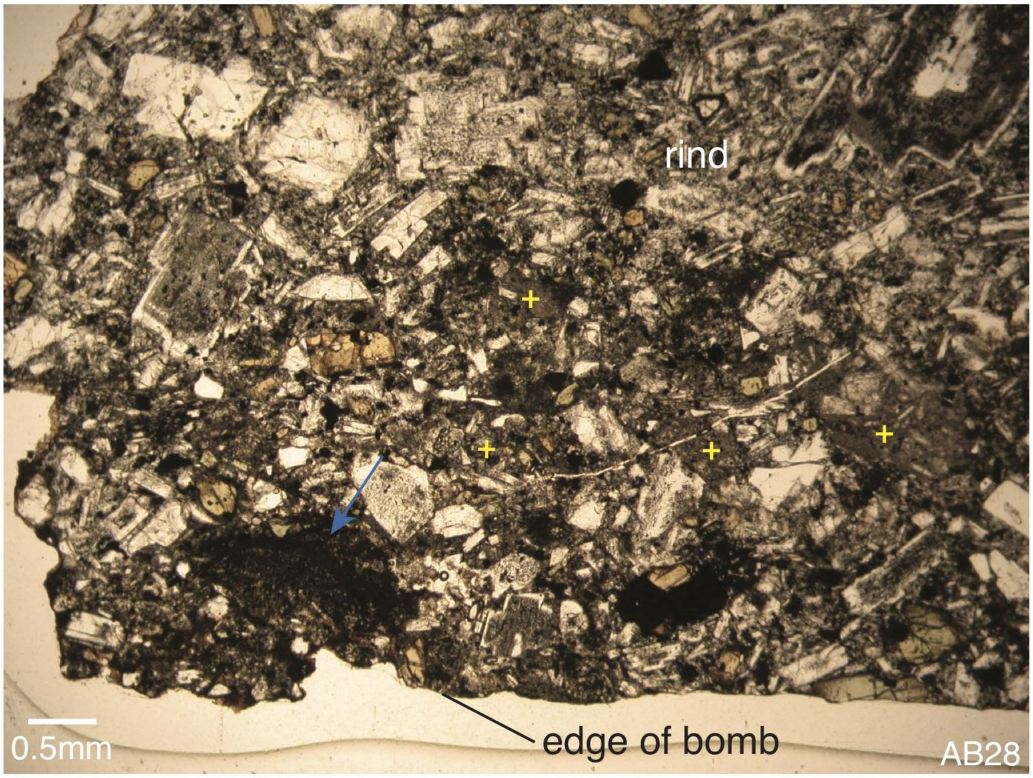

S 5: Plane polarised light image of a cm-scale tuffisite vein in the rind of inflated bomb AB28. Tuffisite material is identifiable by the contrasting textural domains corresponding to different annealed clasts (marked by the yellow crosses) and features zones where the clasts are much finer grained (indicated by the blue arrow). This bomb is the only inflated bomb that records a glass water content <0.4 wt % in the rind.

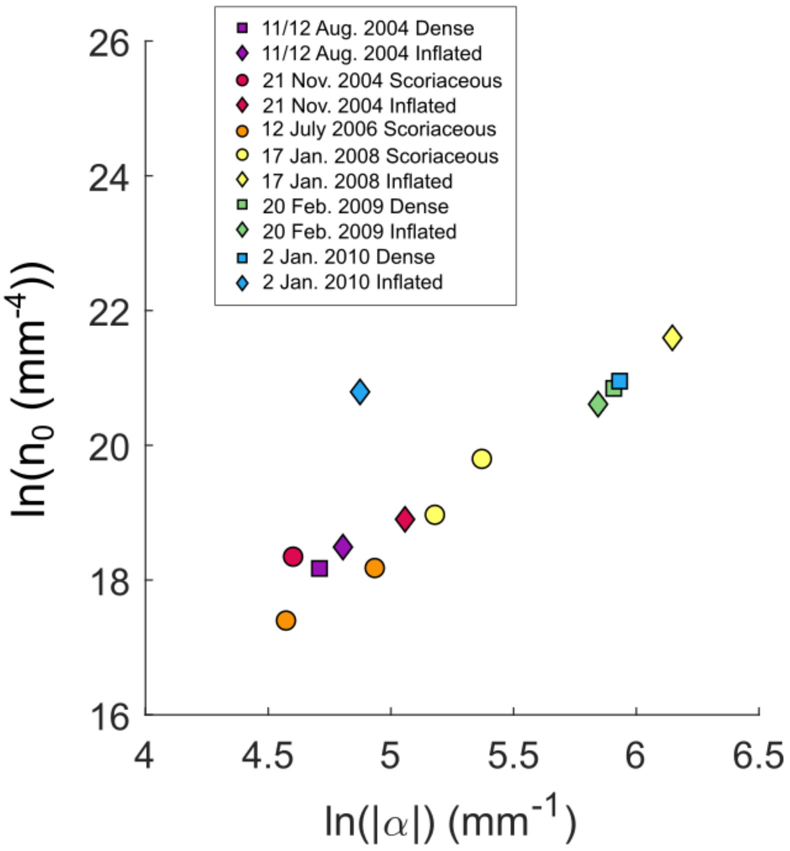

S 6: Final nuclei population density n_0 versus the absolute value of the CSD slope α. n_0 is the y intercept of a line obtained by linear regression through the three points on the steepest part of the CSD for each sample, and α is the slope of that line. Higher n_0 and $|\alpha|$ indicate higher degrees of effective undercooling ΔT, as observed in samples erupted during 2008-2010.

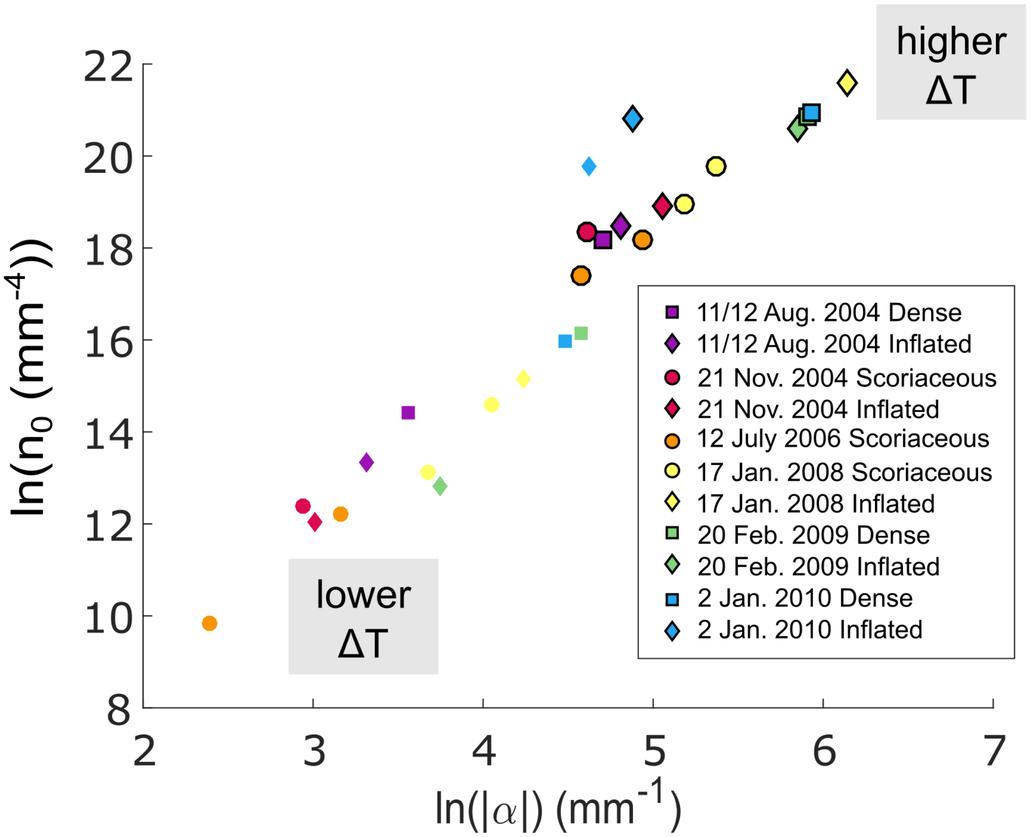

S 7: n_0 and $|\alpha|$ shown for a linear regression performed on the steepest part of the CSD of each sample (black-rimmed symbols) and on the shallowest part of the CSD for each sample (white-rimmed symbols). The patterns of variation in effective undercooling (ΔT) over the course of the eruption period noted for the steepest part of the CSDs are maintained in the shallowest size bins, i.e. ΔT was higher during the crystallisation of the youngest and the oldest groundmass crystals in samples erupted during 2008-2010 compared to the youngest and oldest groundmass crystals in samples erupted during 2004-2006.

References:

Zieg MJ and Marsh BD (2002) Crystal Size Distributions and Scaling Laws in the Quantification of Igneous Textures. J Petrol 43(1):85–101
